# Supplementary material for: Melanin-like nanoparticles slow cyst growth in ADPKD by dual inhibition of oxidative stress and CREB
Source: EMBO Mol Med. 2024 Nov 20;17(1):169–92. doi: 10.1038/s44321-024-00167-2 (PMC11730739; doi:10.1038/s44321-024-00167-2)
Supplement: Supplementary file 1 — Appendix [file 44321_2024_167_MOESM1_ESM.pdf]

Appendix Figures..2

Appendix Figure S1. Characterization of MMPP *in vitro*..2

Appendix Figure S2. MMPP does not affect embryonic kidney growth. 3

Appendix Figure S3. Assessment of kidney function and kidney injury in mice treated with MMPP. 4

Appendix Figure S4. Analysis of oxidative stress in ADPKD kidneys. 5

Appendix Figure S5. MMPP inhibits the expression of CREB target genes in ADPKD cells..6

Appendix Figure S6. Intracellular distribution of MMPP in renal proximal tubular cells..7

Appendix Figure S7. MMPP inhibits CREB transcriptional activity independent of ROS. 8

Appendix Tables..9

Appendix Table S1. Primers used for RT-PCR. 9

Appendix Table S2. Primers used for ChIP-qPCR. 11

Appendix Figures

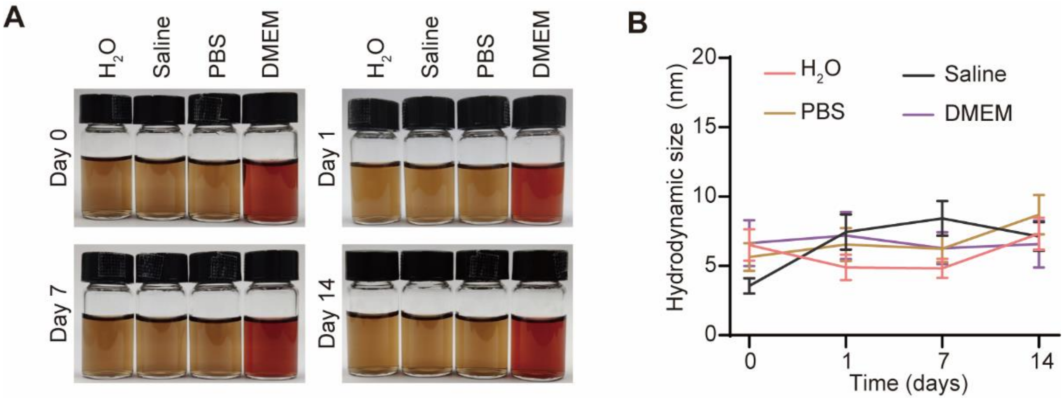

Appendix Figure S1. Characterization of MMPP *in vitro*.

(A) Photographic representation of MMPP nanoparticles dispersed in various media at different days. (B) Hydrodynamic size assessment in different media over a 14-day period. Data presented as means ± SD.

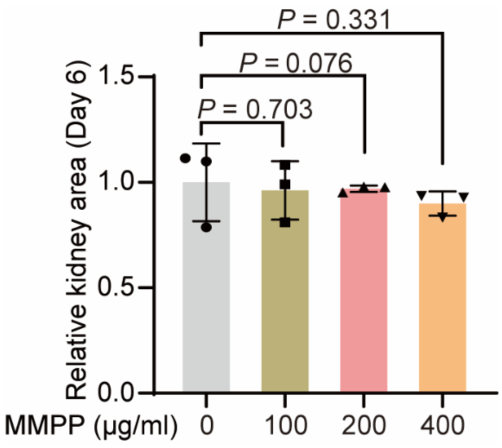

Appendix Figure S2. MMPP does not affect embryonic kidney growth.

Total area of embryonic kidneys treated with indicated concentration of MMPP for six days (representative of three independent experiments; n = 3 biological replicates). Data presented as means ± SD. One-way ANOVA with Dunnett's T3 test was used for statistical analysis.

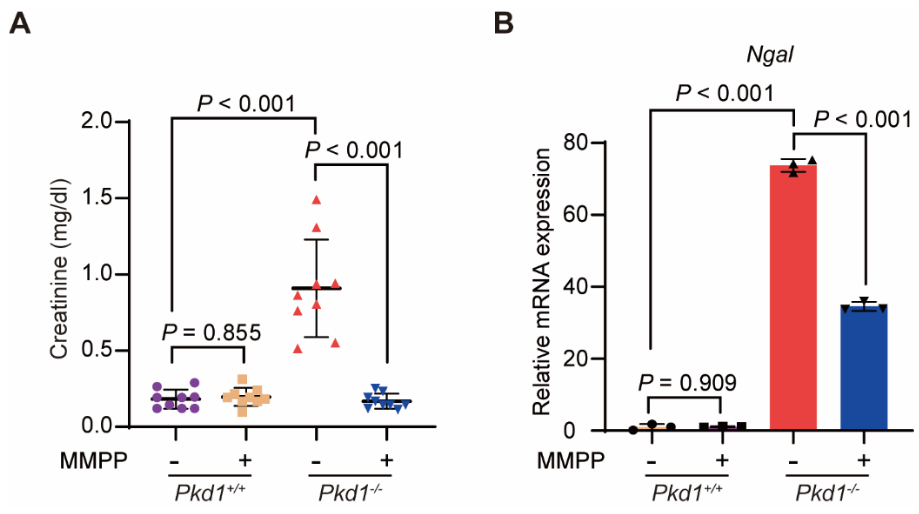

Appendix Figure S3. Assessment of kidney function and kidney injury in mice treated with MMPP.

(A) Serum creatinine levels in MMPP-treated mice from the indicated groups (n = 9). (B) Quantitative RT-PCR analysis of relative mRNA levels of injury marker from the indicated groups (representative of three independent experiments; n = 3 biological replicates). Data presented as means ± SD. Two-way ANOVA with LSD test was used for statistical analysis.

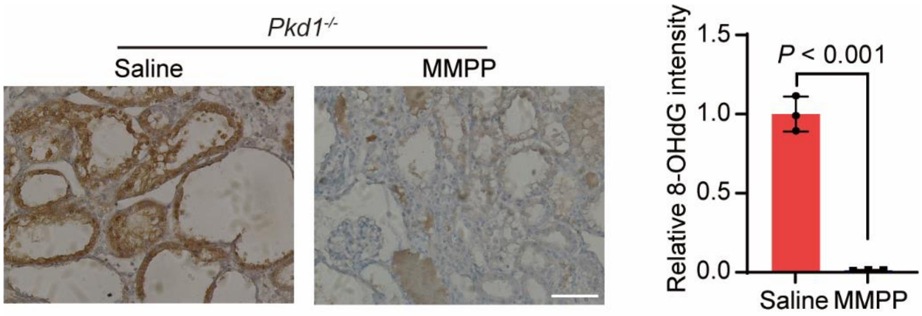

Appendix Figure S4. Analysis of oxidative stress in ADPKD kidneys.

Immunostaining of 8-OHdG (left) and quantification (right) in ADPKD kidneys with or without MMPP treatment (representative of three independent experiments; n = 3 biological replicates). Scale bar, 50 μm. Data presented as means ± SD. A two-tailed unpaired Student's t test was used for statistical analysis.

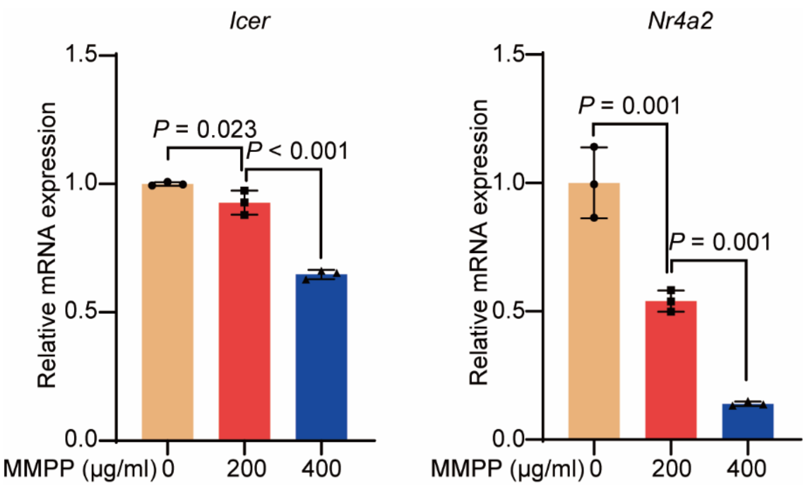

Appendix Figure S5. MMPP inhibits the expression of CREB target genes in ADPKD cells.

RT-PCR analysis of representative CREB target genes (*Icer* and *Nr4a2*) in PN cells with or without MMPP treatment (representative of three independent experiments; n = 3 biological replicates). Data presented as means ± SD. One-way ANOVA with LSD test was used for statistical analysis.

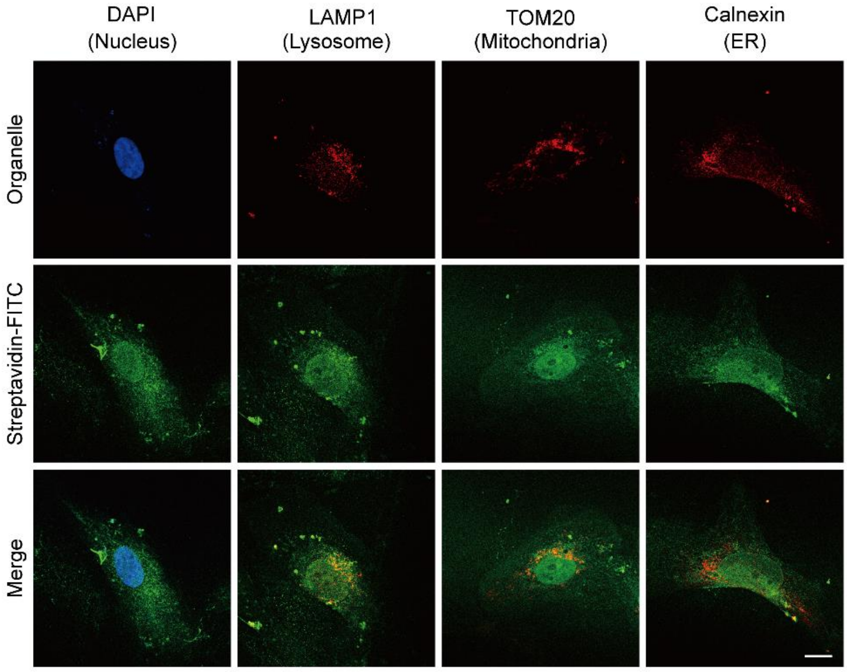

Appendix Figure S6. Intracellular distribution of MMPP in renal proximal tubular cells.

Co-staining of Bio-MMPP with the indicated organelle markers in HK-2 cells. Scale bar, 10 μm.

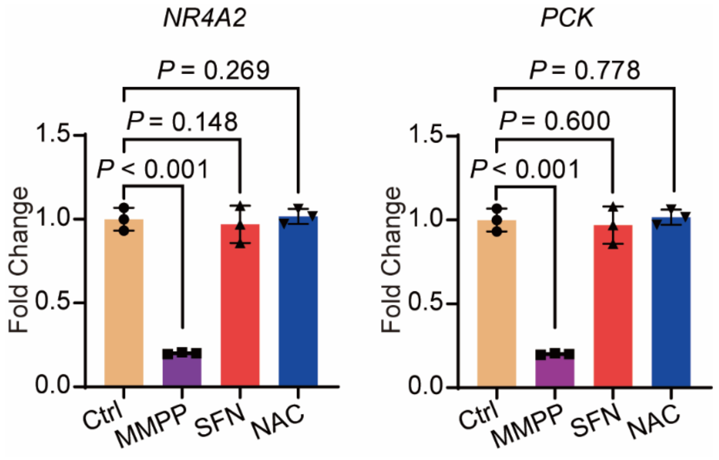

Appendix Figure S7. MMPP inhibits CREB transcriptional activity independent of ROS.

ChIP-qPCR analysis of p-CREB occupancy on NR4A2 (left) and PCK (right) genes in WT 9-12 cells treated with MMPP or antioxidants (representative of three independent experiments; n = 3 biological replicates). Data presented as means ± SD. One-way ANOVA with LSD test was used for statistical analysis.

Appendix Tables

Appendix Table S1. Primers used for RT-PCR.

Co3 F	CAAGGCCACCACACTCCTAT
Co3 R	GCAGCCTCCTAGATCATGTGT
Cytb F	TGCATACGCCATTCTACGCT
Cytb R	AGGCTTCGTTGCTTTGAGGT
Atp6 F	GCAGTCCGGCTTACAGCTAA
Atp6 R	GGTAGCTGTTGGTGGGCTAA
Myc F	ATGCCCCTCAACGTGAACTTC
Myc R	CGCAACATAGGATGGAGAGCA
Syk F	CTACCTGCTACGCCAGAGC
Syk R	GCCATTAAGTTCCCTCTCGATG
Abcg1 F	CTTTCCTACTCTGTACCCGAGG
Abcg1 R	CGGGGCATTCCATTGATAAGG
Clcf1 F	GACTCGTGGGGGATGTTAGC
Clcf1 R	CTAAGCTGCGGAGTTGATGCT
Icre F	GGTGACATGCCAACTTACCAGA
Icer R	TTGCGACTTGCTTCTTCTGC
Nr4a2 F	GTGTTCAGGCGCAGTATGG
Nr4a2 R	TGGCAGTAATTTCAGTGTTGGT
ICER F	ATGGCTGTAACTGGAGATGACACAG
ICER R	CTCCCTGTGGCAAAGCAGCA
NR4A2 F	ACCTGTGAGGGCTGCAAAG
NR4A2 R	AGGCACTTCTGAAATCGGCA
GAPDH F	TTGCCCTCAACGACCACTTT
GAPDH R	TGGTCCAGGGGTCTTACTCC
Ngal F	GACTTCCGGAGCGATCAGTT

Ngal R	CTGATCCAGTAGCGACAGCC
Actin F	TTGCTGACAGGATGCAGAAG
Actin R	ATCCACATCTGCTGGAAGGT

Appendix Table S2. Primers used for ChIP-qPCR.

NR4A2 F	CTGAGCTGGAGTTGCTGCTA
NR4A2 R	GGAGAGCTGGGGAGAAGGAA
PCK F	GAGGTTCTGCCACCAAGCA
PCK R	TCGAAGGGAGATCCACAGGT
Abcg1 F	GCCGTATAGCTTTTGCCCG
Abcg1 R	TTGTCTCTAGGCTGTTGCCA
Clcf1 F	AACAAAGTCTGTCCCACTCCAG
Clcf1 R	CAGTCATGGCCAGTGCTAGTT
