# Supplementary figures and images for: Melanin-like nanoparticles slow cyst growth in ADPKD by dual inhibition of oxidative stress and CREB
Source: EMBO Mol Med. 2024 Nov 20;17(1):169–92. doi: 10.1038/s44321-024-00167-2 (PMC11730739; doi:10.1038/s44321-024-00167-2)

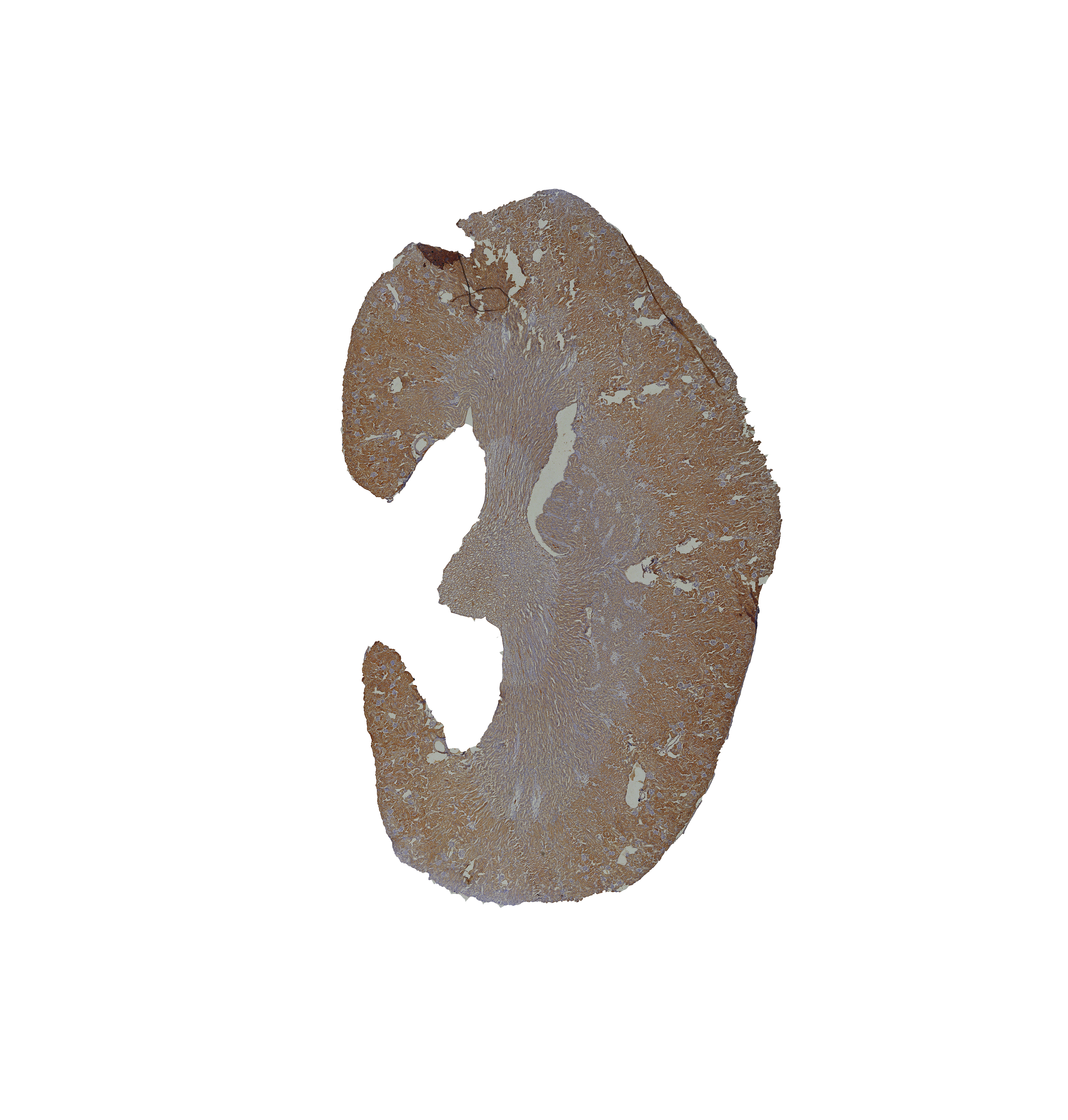

Supplement: Supplementary file 3 — Source data Fig. 1 [file 44321_2024_167_MOESM3_ESM.zip › EMM-2024-20280_Source data for Figure 1/1E/MMPP.jpg]

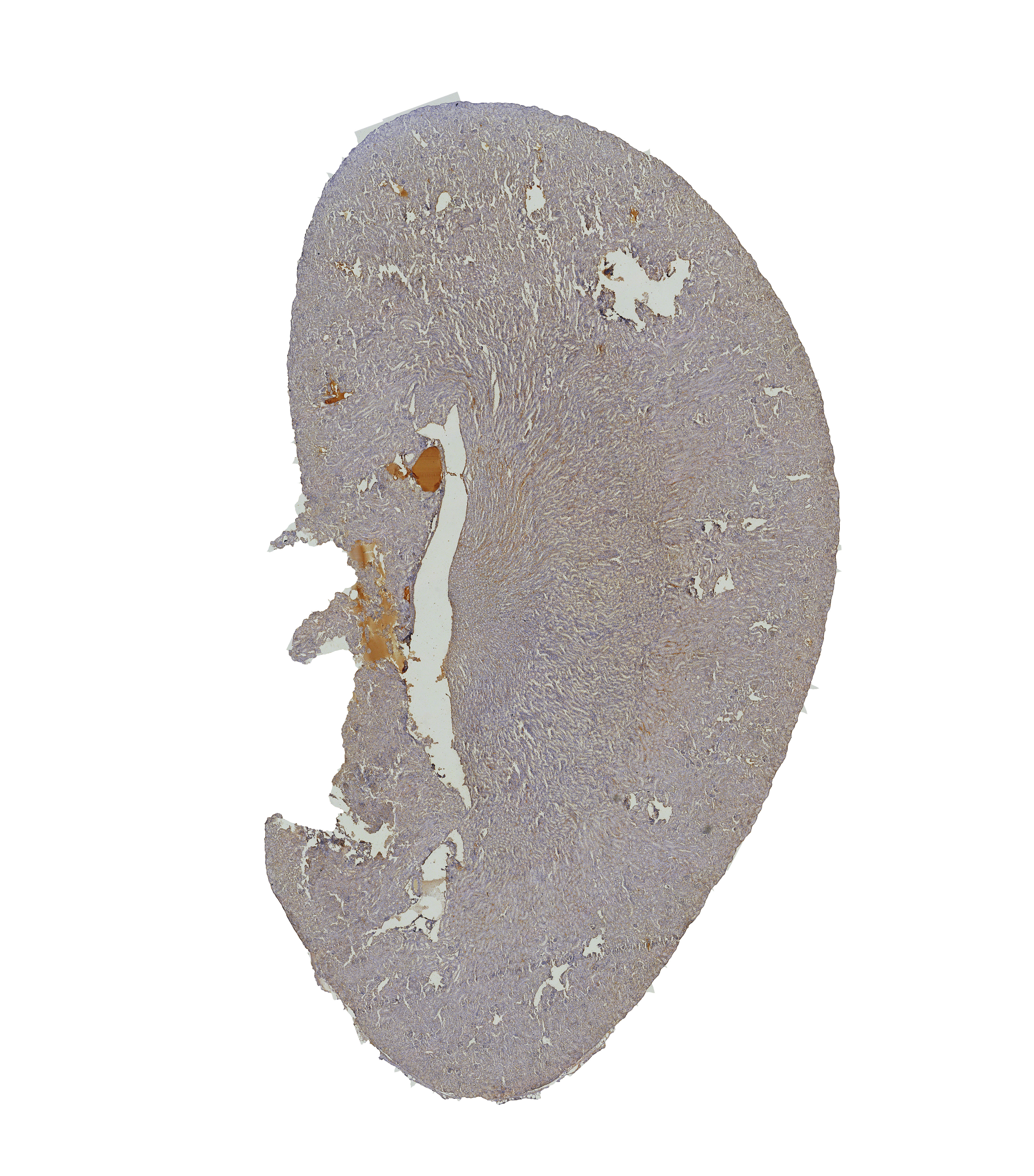

Supplement: Supplementary file 3 — Source data Fig. 1 [file 44321_2024_167_MOESM3_ESM.zip › EMM-2024-20280_Source data for Figure 1/1E/Saline.jpg]

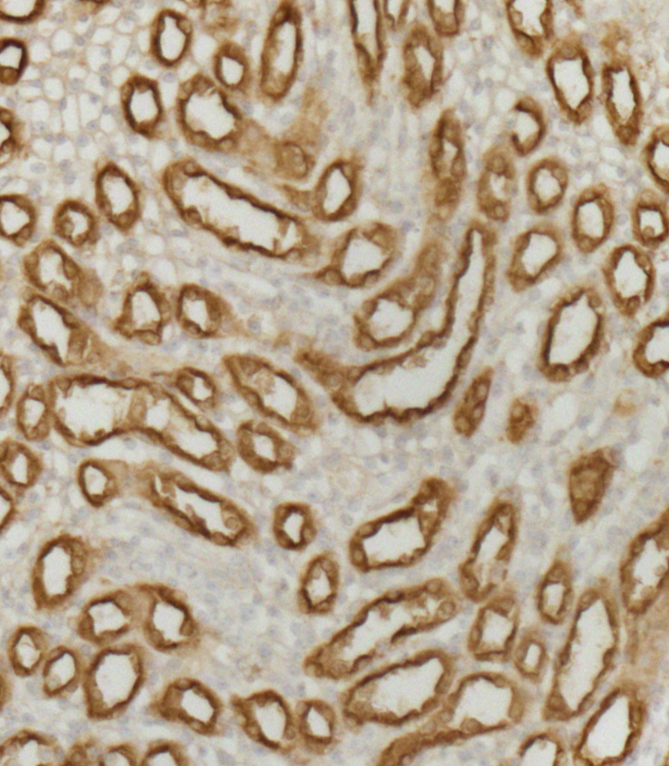

Supplement: Supplementary file 3 — Source data Fig. 1 [file 44321_2024_167_MOESM3_ESM.zip › EMM-2024-20280_Source data for Figure 1/1F/AQP2.tif]

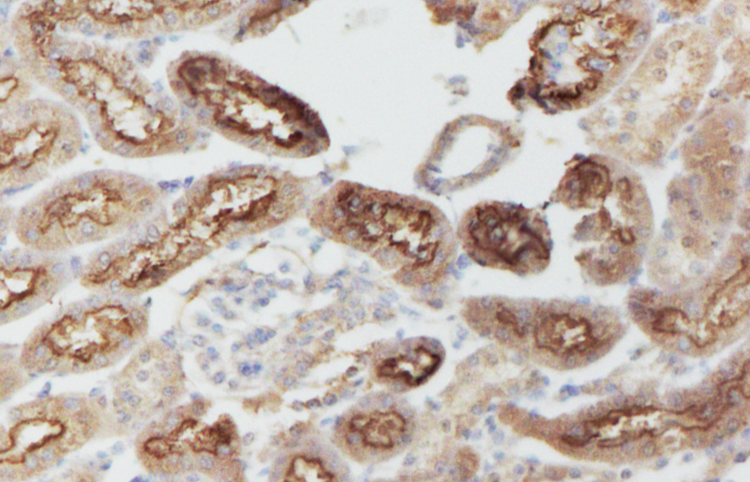

Supplement: Supplementary file 3 — Source data Fig. 1 [file 44321_2024_167_MOESM3_ESM.zip › EMM-2024-20280_Source data for Figure 1/1F/LRP2.tif]

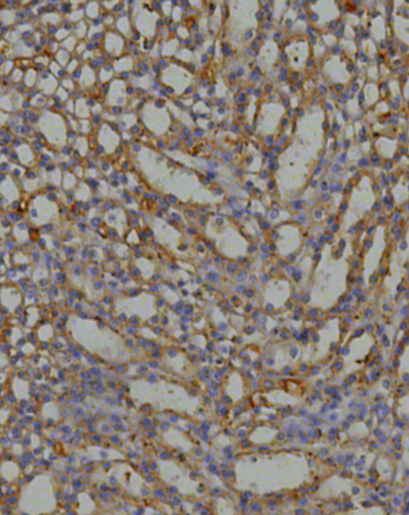

Supplement: Supplementary file 3 — Source data Fig. 1 [file 44321_2024_167_MOESM3_ESM.zip › EMM-2024-20280_Source data for Figure 1/1F/PEG(AQP2).tif]

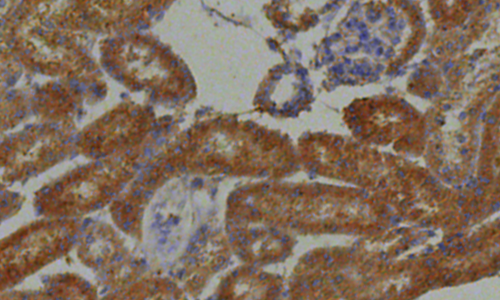

Supplement: Supplementary file 3 — Source data Fig. 1 [file 44321_2024_167_MOESM3_ESM.zip › EMM-2024-20280_Source data for Figure 1/1F/PEG(LRP2).tif]

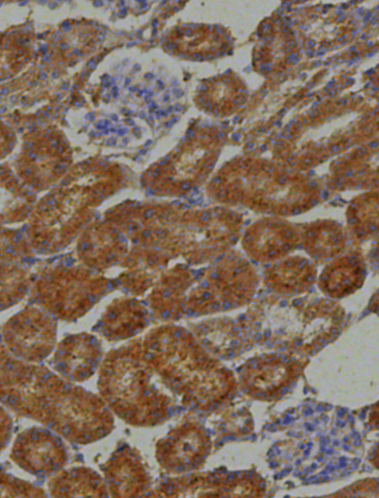

Supplement: Supplementary file 3 — Source data Fig. 1 [file 44321_2024_167_MOESM3_ESM.zip › EMM-2024-20280_Source data for Figure 1/1F/PEG(SYNPO).tif]

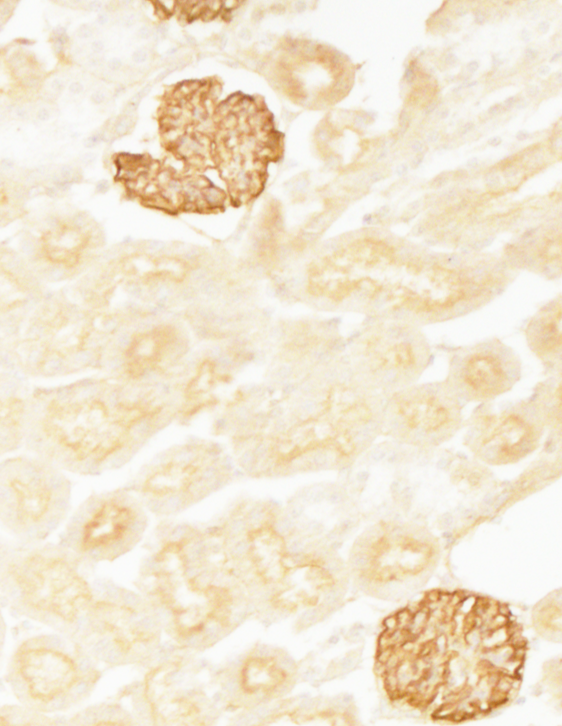

Supplement: Supplementary file 3 — Source data Fig. 1 [file 44321_2024_167_MOESM3_ESM.zip › EMM-2024-20280_Source data for Figure 1/1F/SYNPO.tif]

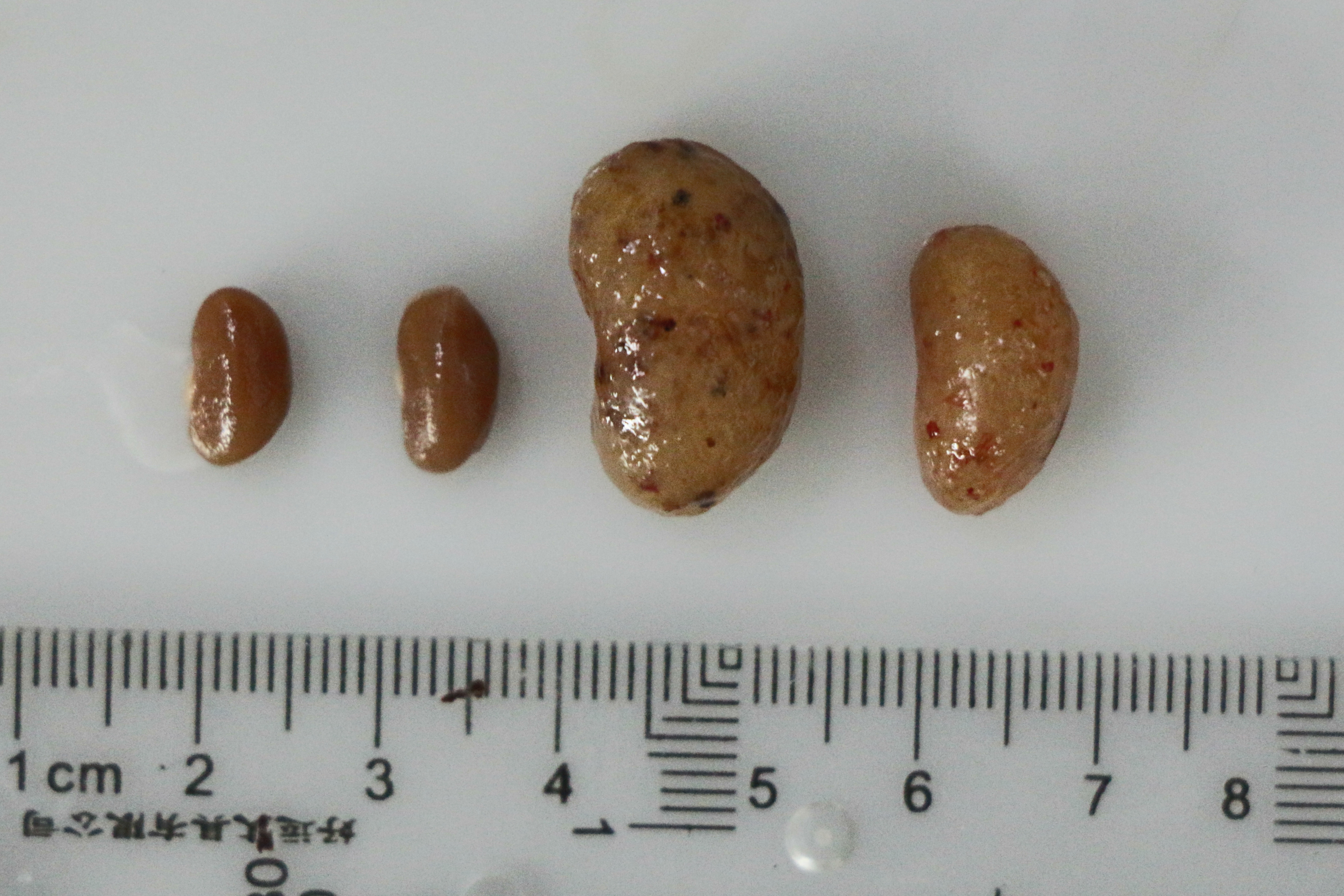

Supplement: Supplementary file 4 — Source data Fig. 2 [file 44321_2024_167_MOESM4_ESM.zip › EMM-2024-20280_Source data for Figure 2/2B/kidney.JPG]

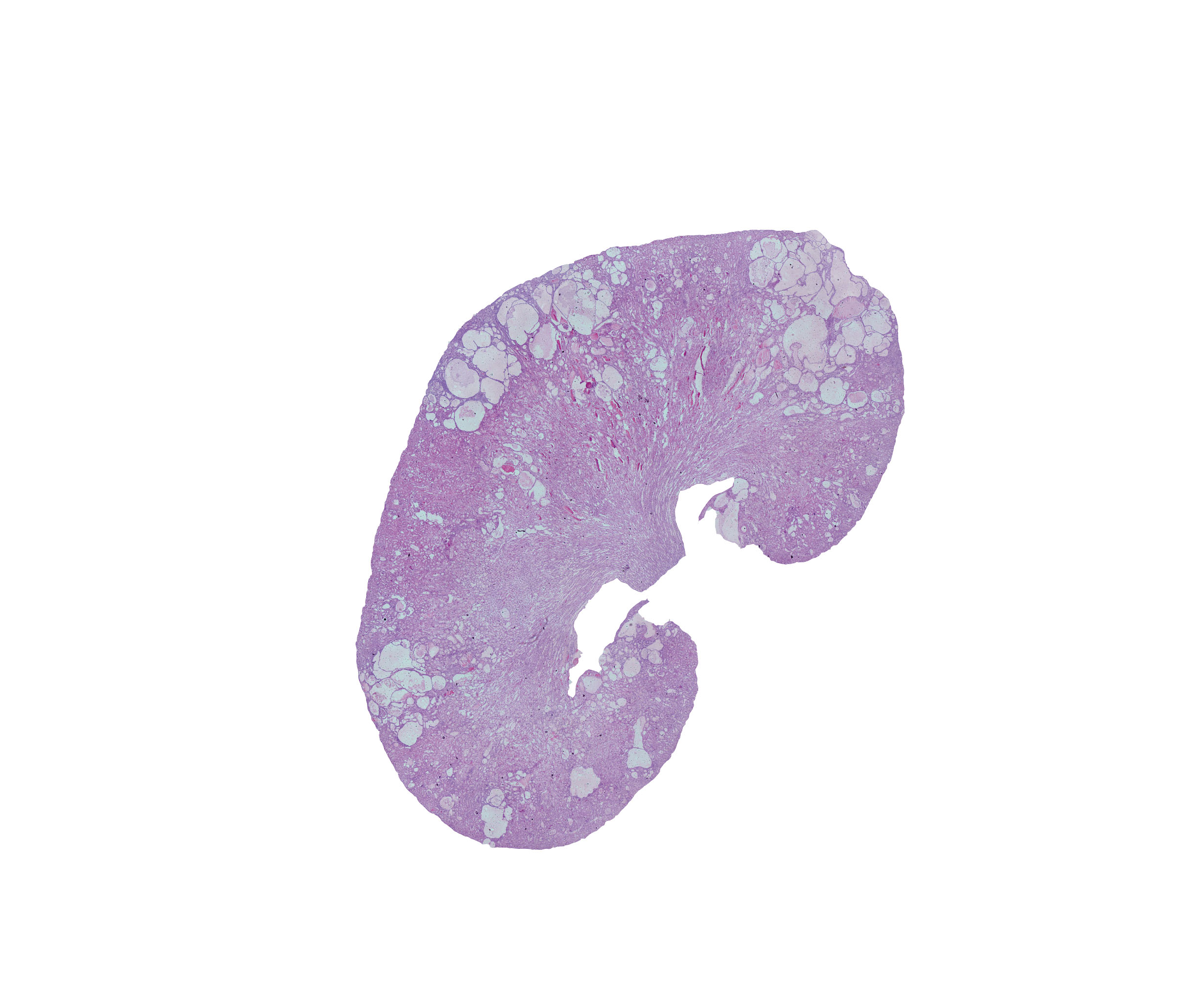

Supplement: Supplementary file 4 — Source data Fig. 2 [file 44321_2024_167_MOESM4_ESM.zip › EMM-2024-20280_Source data for Figure 2/2D/MMPP.jpg]

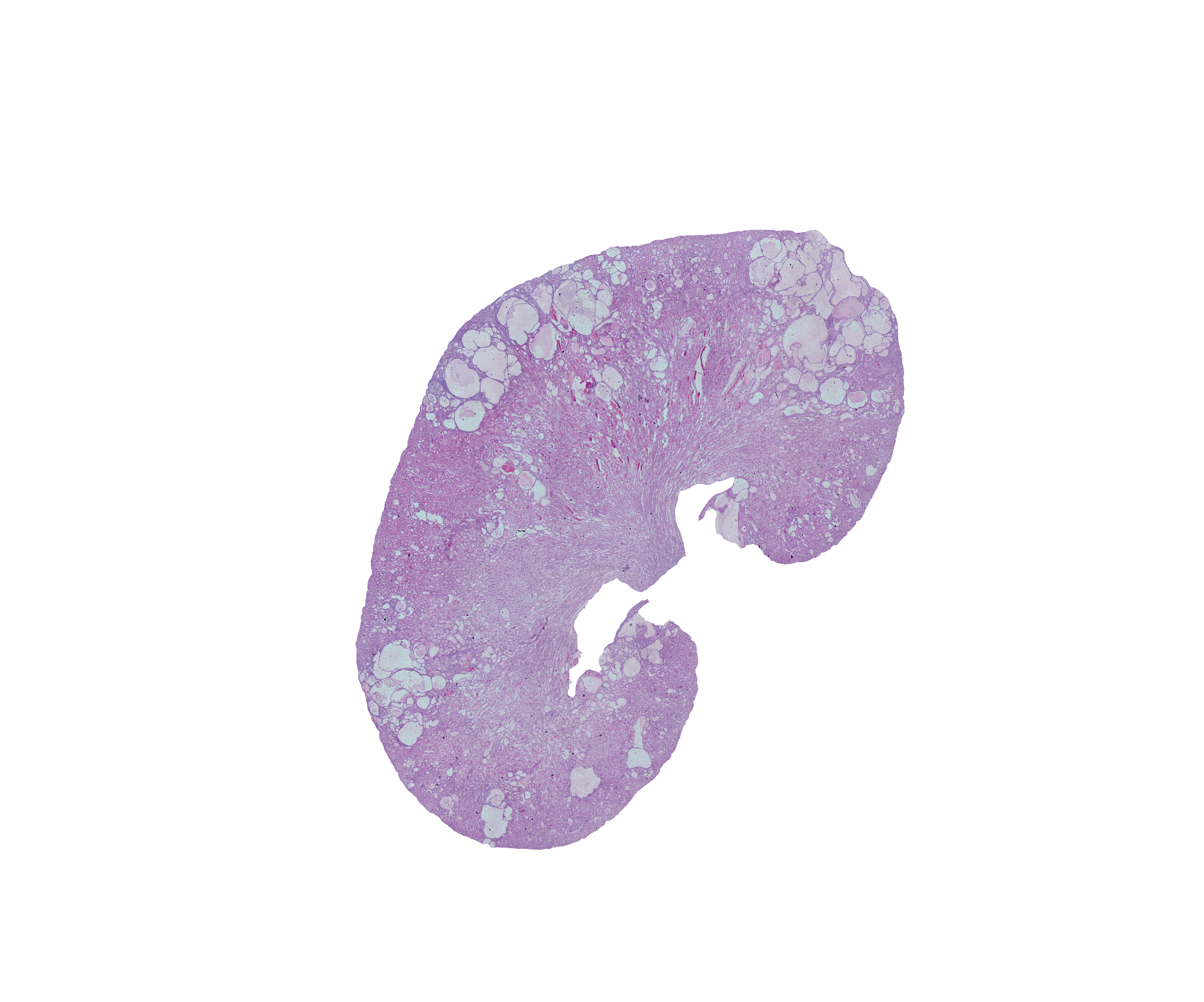

Supplement: Supplementary file 4 — Source data Fig. 2 [file 44321_2024_167_MOESM4_ESM.zip › EMM-2024-20280_Source data for Figure 2/2D/MMPP.tif]

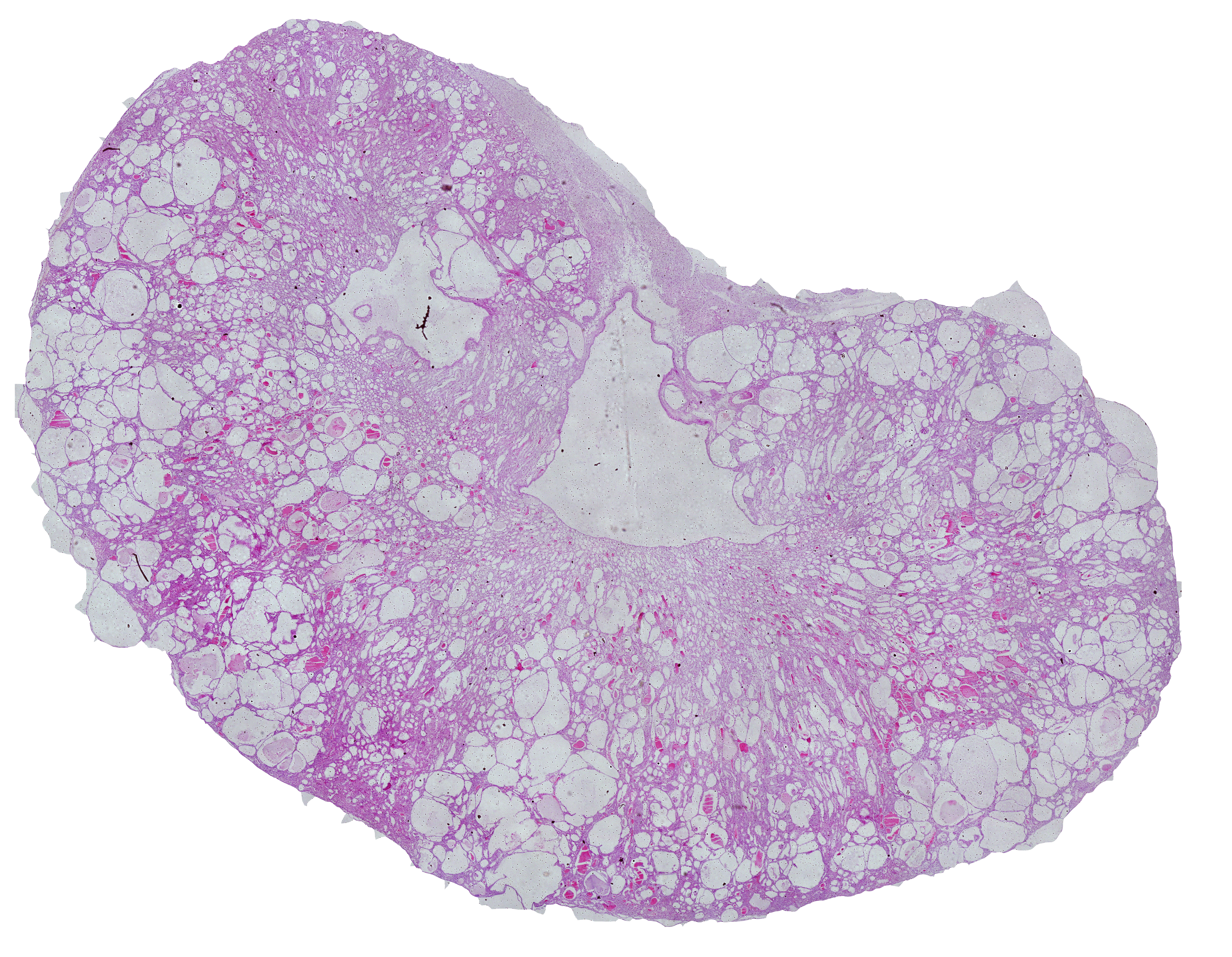

Supplement: Supplementary file 4 — Source data Fig. 2 [file 44321_2024_167_MOESM4_ESM.zip › EMM-2024-20280_Source data for Figure 2/2D/Saline.jpg]

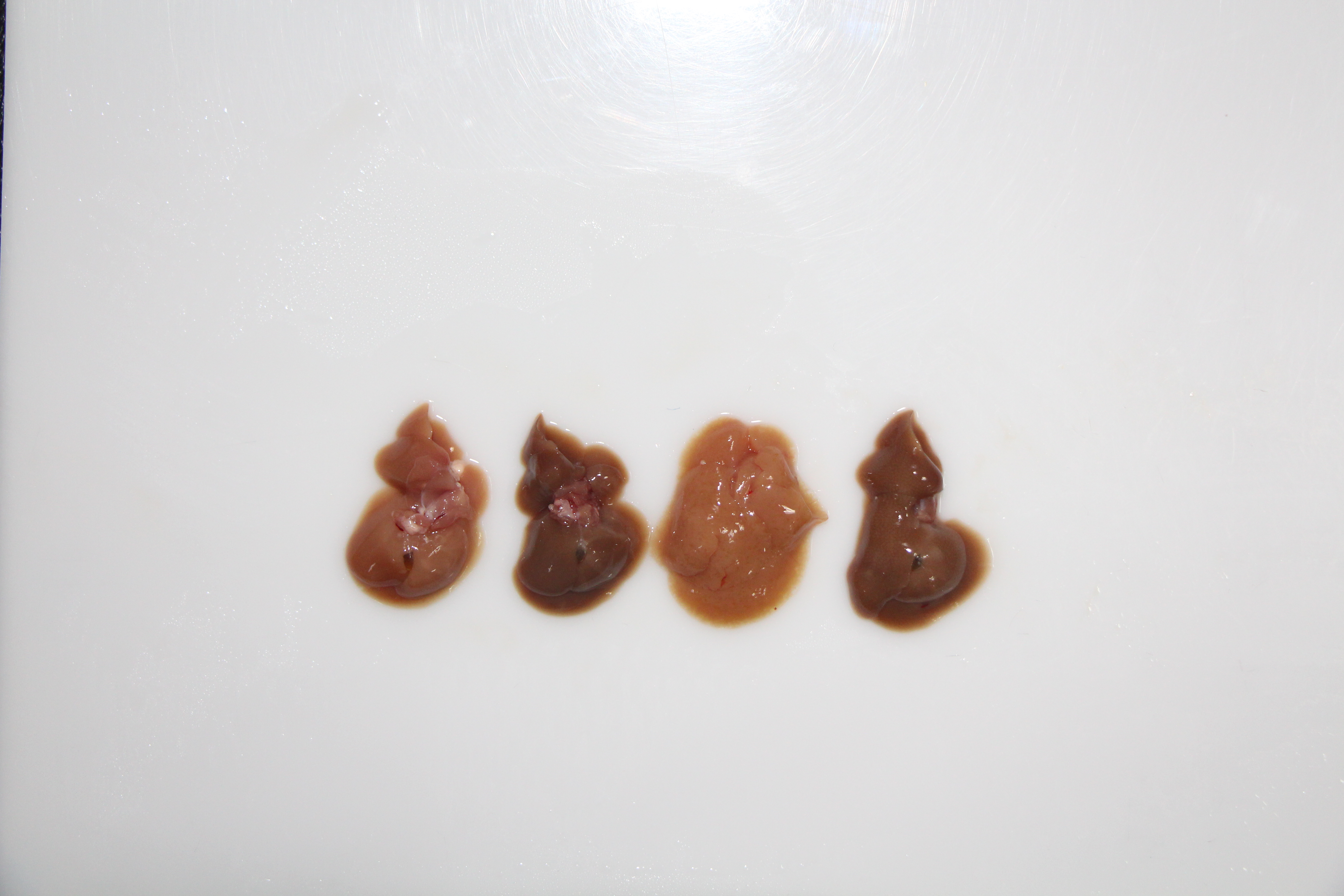

Supplement: Supplementary file 4 — Source data Fig. 2 [file 44321_2024_167_MOESM4_ESM.zip › EMM-2024-20280_Source data for Figure 2/2G/liver.JPG]

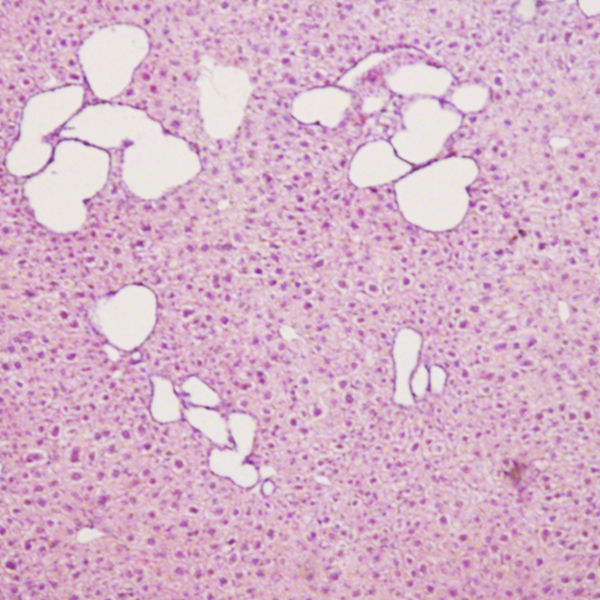

Supplement: Supplementary file 4 — Source data Fig. 2 [file 44321_2024_167_MOESM4_ESM.zip › EMM-2024-20280_Source data for Figure 2/2H/MMPP.tif]

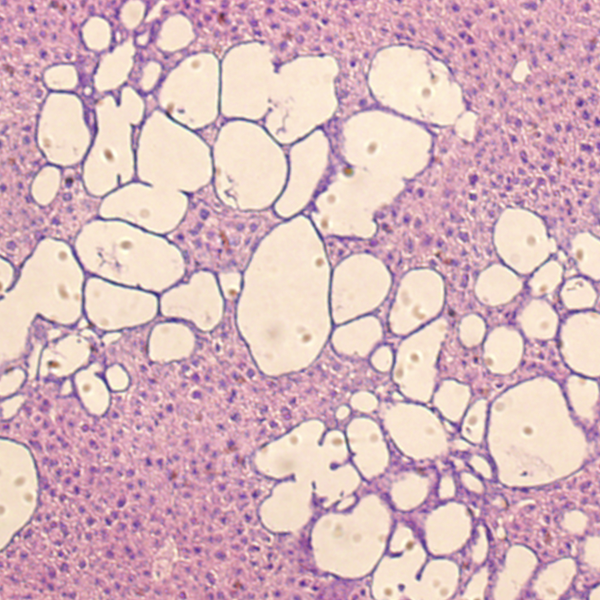

Supplement: Supplementary file 4 — Source data Fig. 2 [file 44321_2024_167_MOESM4_ESM.zip › EMM-2024-20280_Source data for Figure 2/2H/Saline.tif]

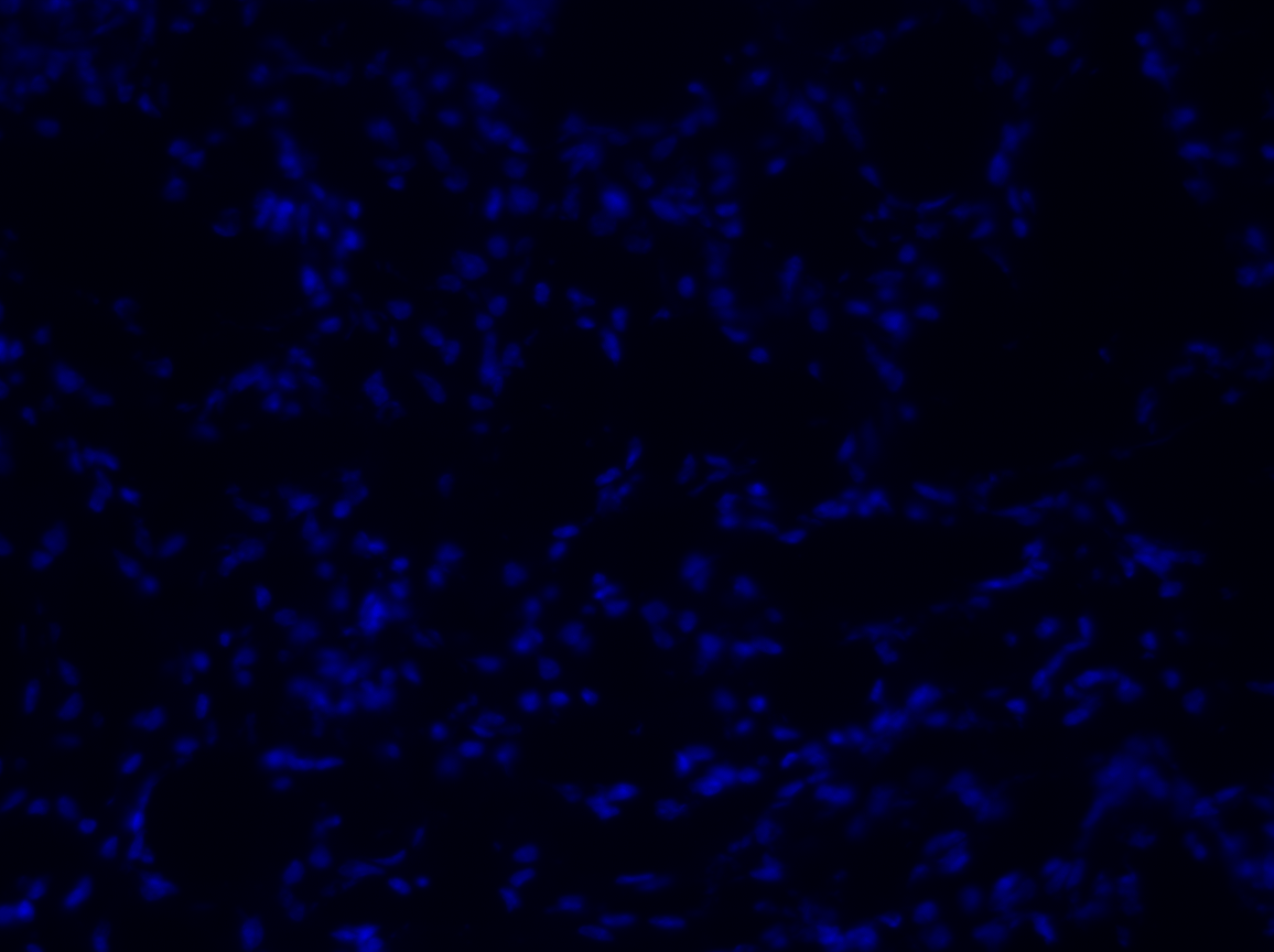

Supplement: Supplementary file 5 — Source data Fig. 3 [file 44321_2024_167_MOESM5_ESM.zip › EMM-2024-20280_Source data for Figure 3/3E/ADPKD+MMPP DAPI.tif]

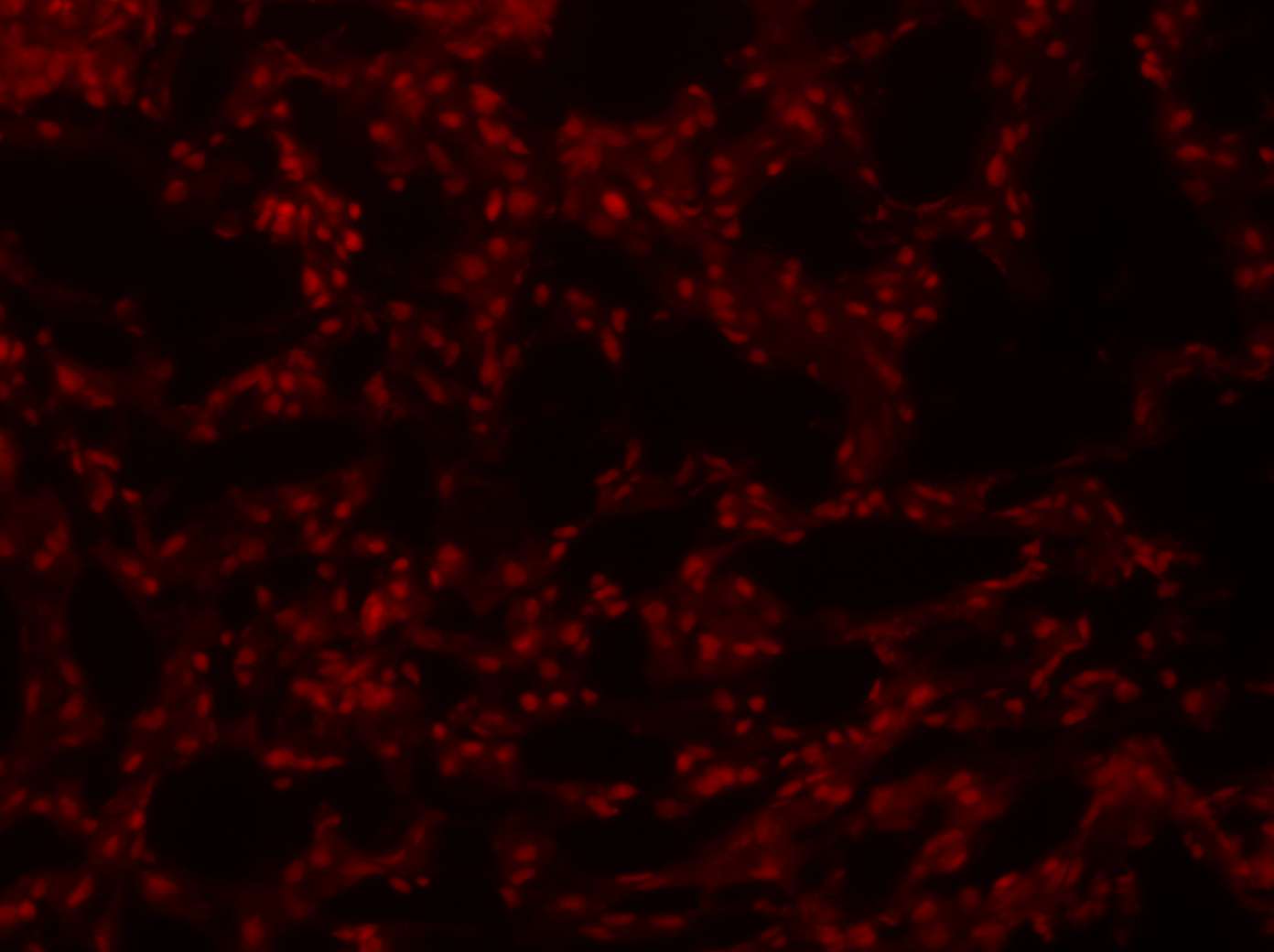

Supplement: Supplementary file 5 — Source data Fig. 3 [file 44321_2024_167_MOESM5_ESM.zip › EMM-2024-20280_Source data for Figure 3/3E/ADPKD+MMPP DHE.tif]

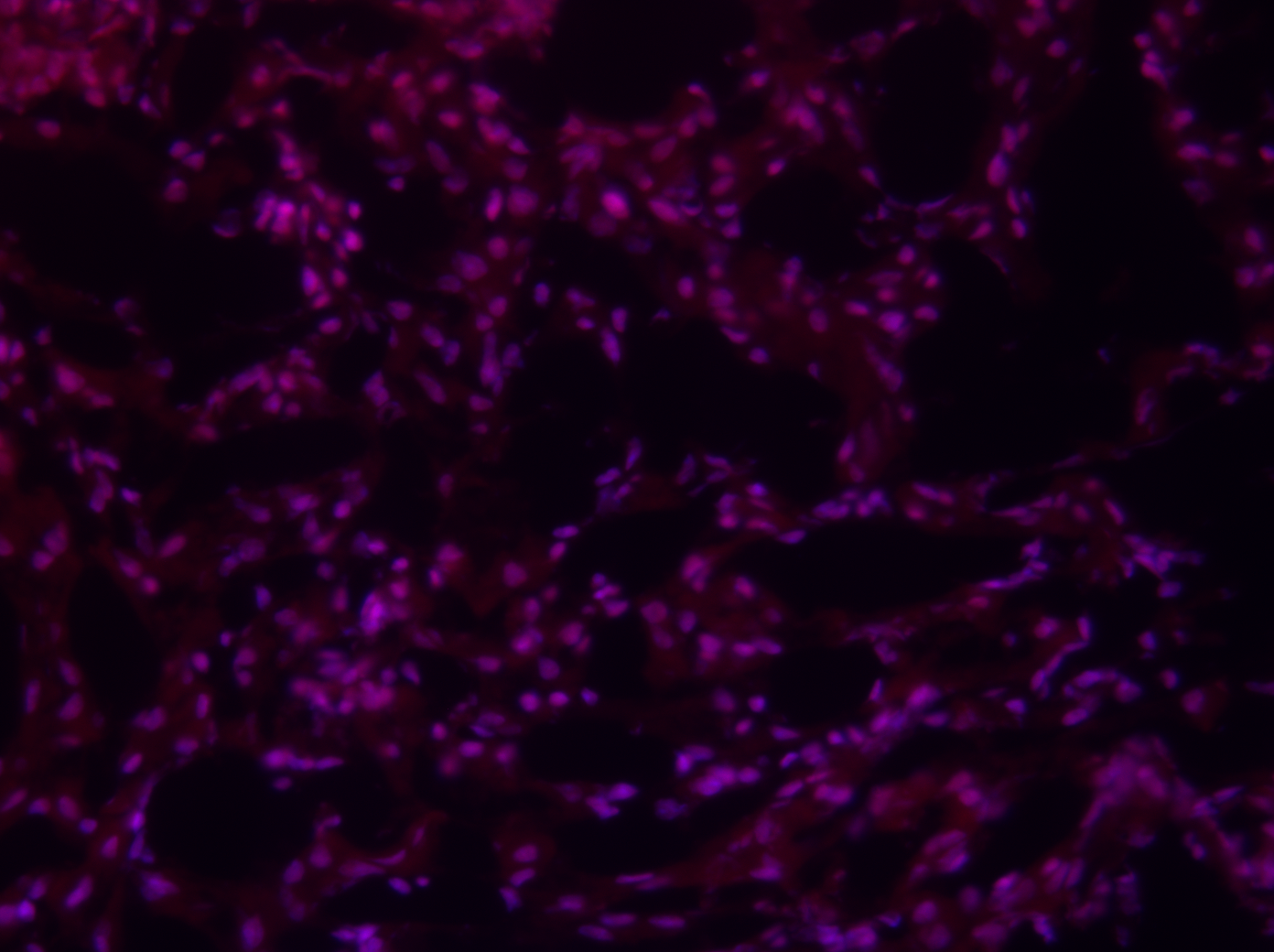

Supplement: Supplementary file 5 — Source data Fig. 3 [file 44321_2024_167_MOESM5_ESM.zip › EMM-2024-20280_Source data for Figure 3/3E/ADPKD+MMPP Merge.tif]

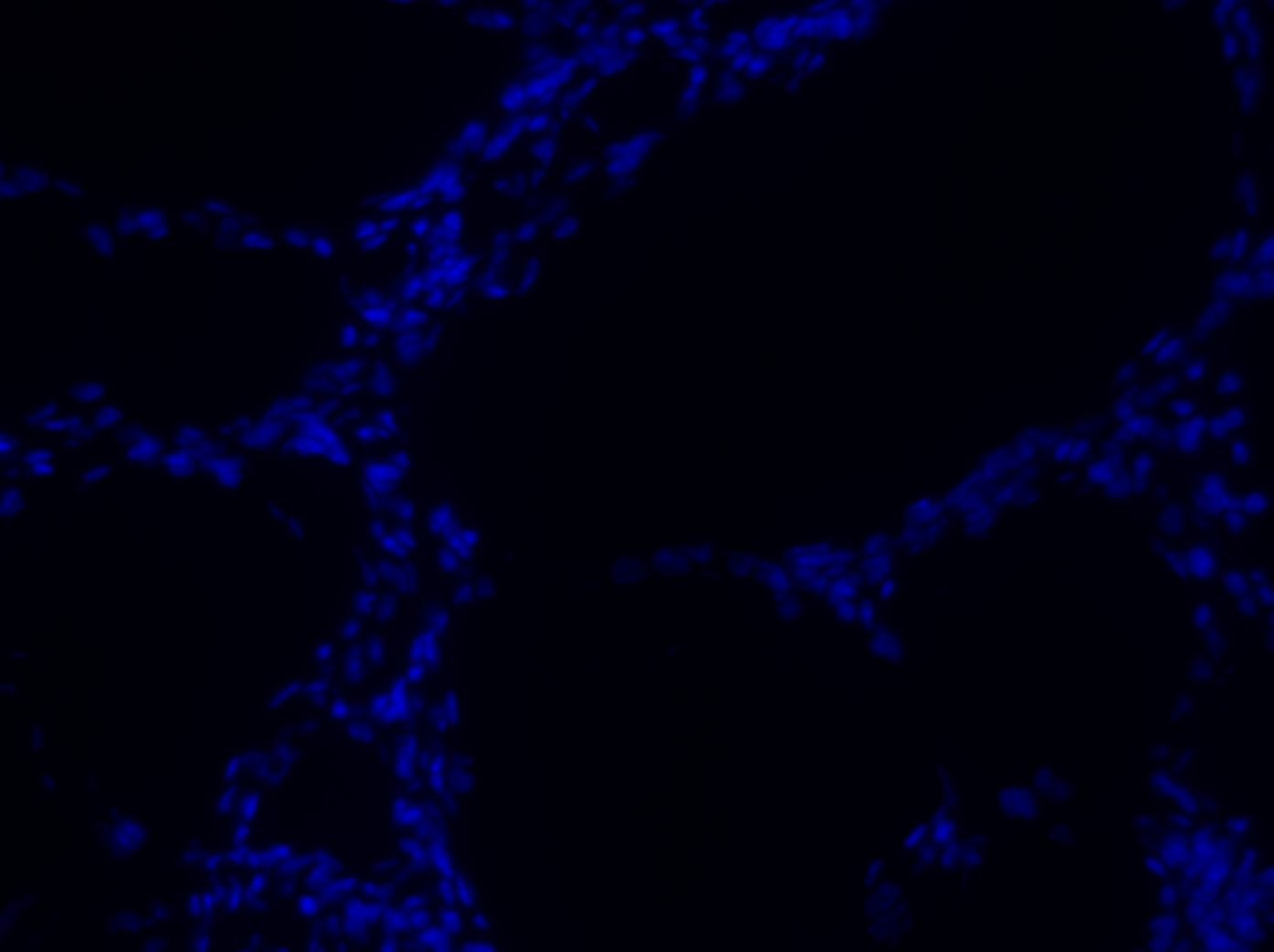

Supplement: Supplementary file 5 — Source data Fig. 3 [file 44321_2024_167_MOESM5_ESM.zip › EMM-2024-20280_Source data for Figure 3/3E/ADPKD+Saline DAPI.tif]

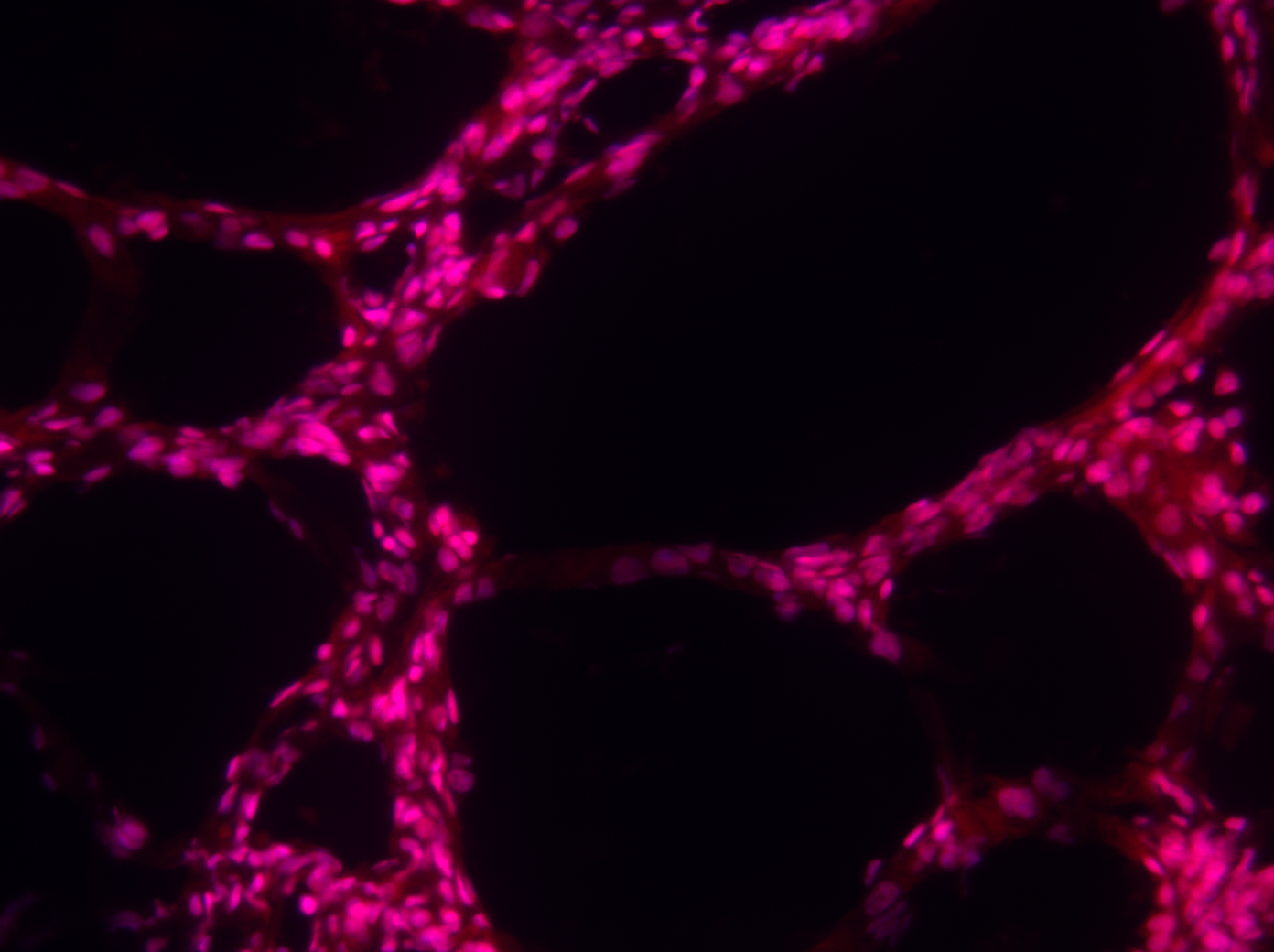

Supplement: Supplementary file 5 — Source data Fig. 3 [file 44321_2024_167_MOESM5_ESM.zip › EMM-2024-20280_Source data for Figure 3/3E/ADPKD+Saline Merge.tif]

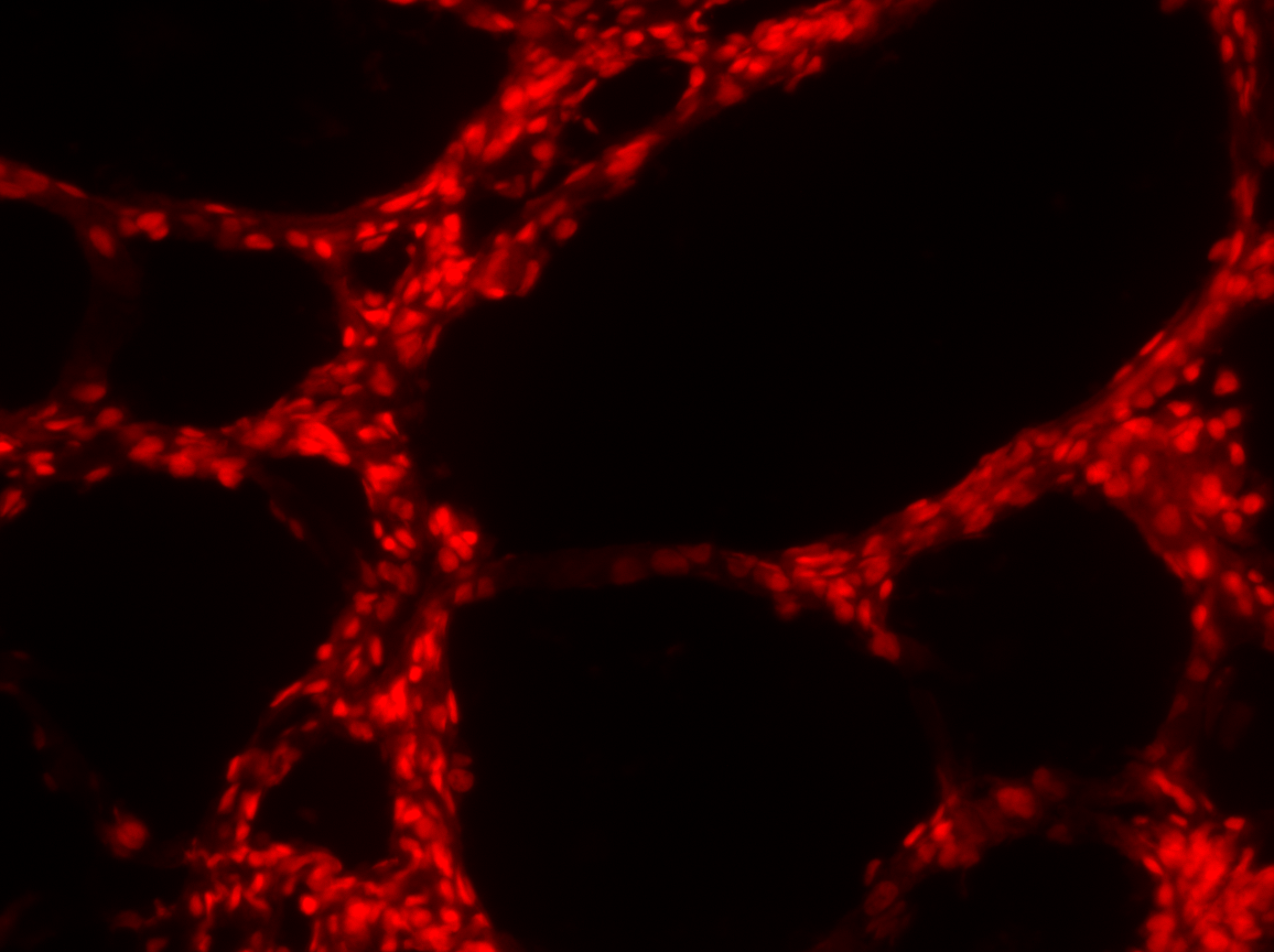

Supplement: Supplementary file 5 — Source data Fig. 3 [file 44321_2024_167_MOESM5_ESM.zip › EMM-2024-20280_Source data for Figure 3/3E/ADPKD+Salnie DHE.tif]

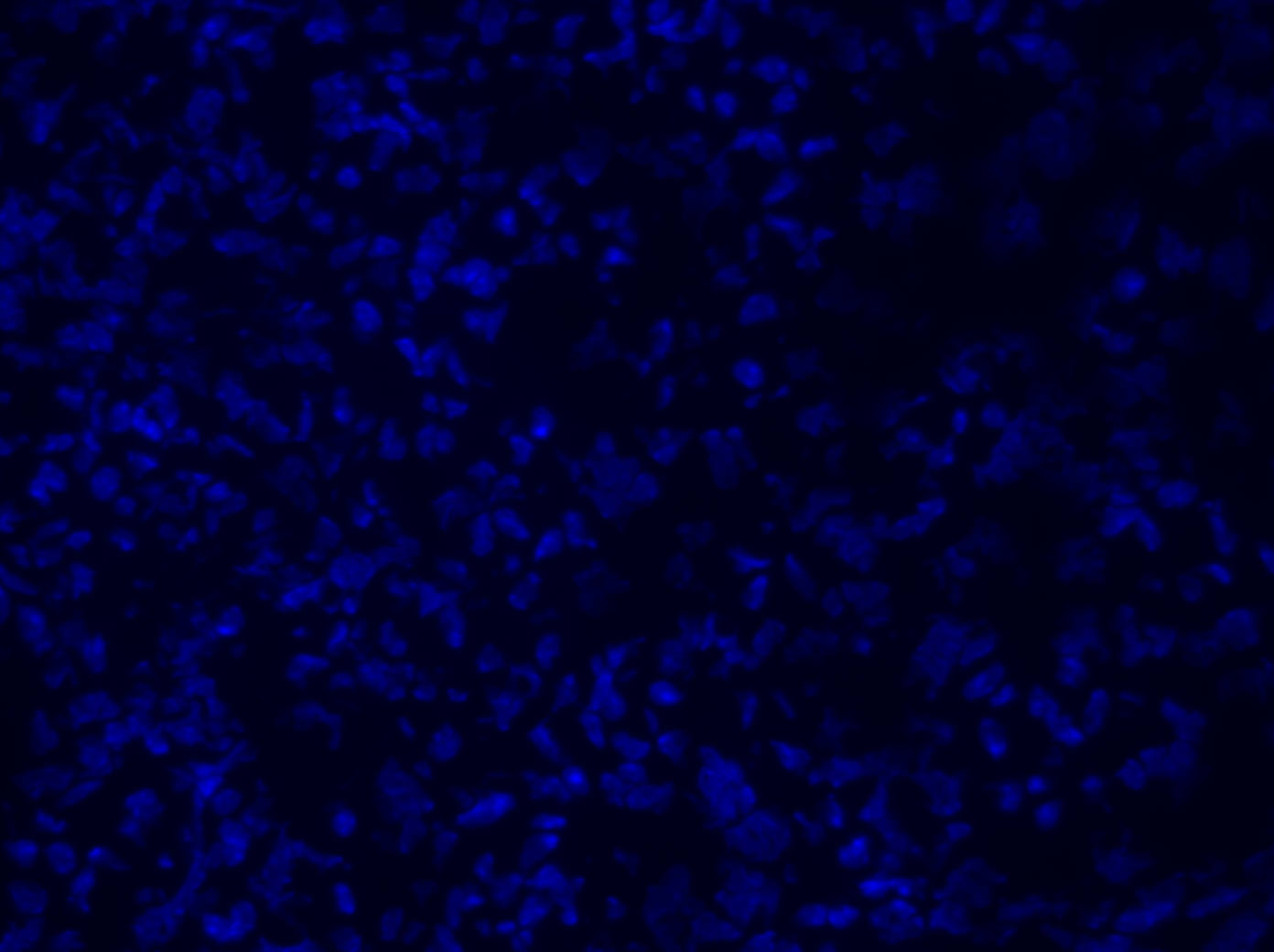

Supplement: Supplementary file 5 — Source data Fig. 3 [file 44321_2024_167_MOESM5_ESM.zip › EMM-2024-20280_Source data for Figure 3/3E/WT+Saline DAPI.tif]

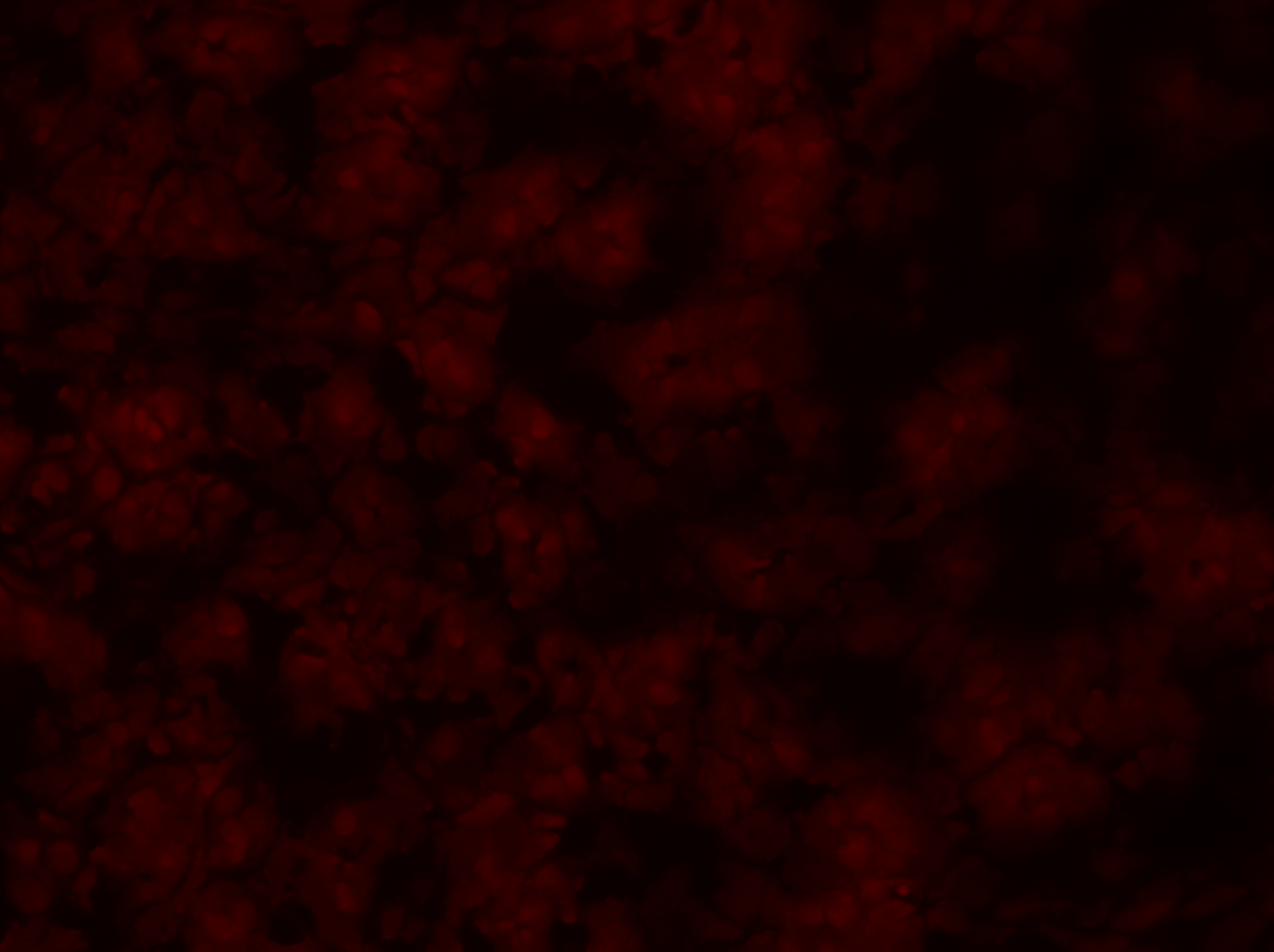

Supplement: Supplementary file 5 — Source data Fig. 3 [file 44321_2024_167_MOESM5_ESM.zip › EMM-2024-20280_Source data for Figure 3/3E/WT+Saline DHE.tif]

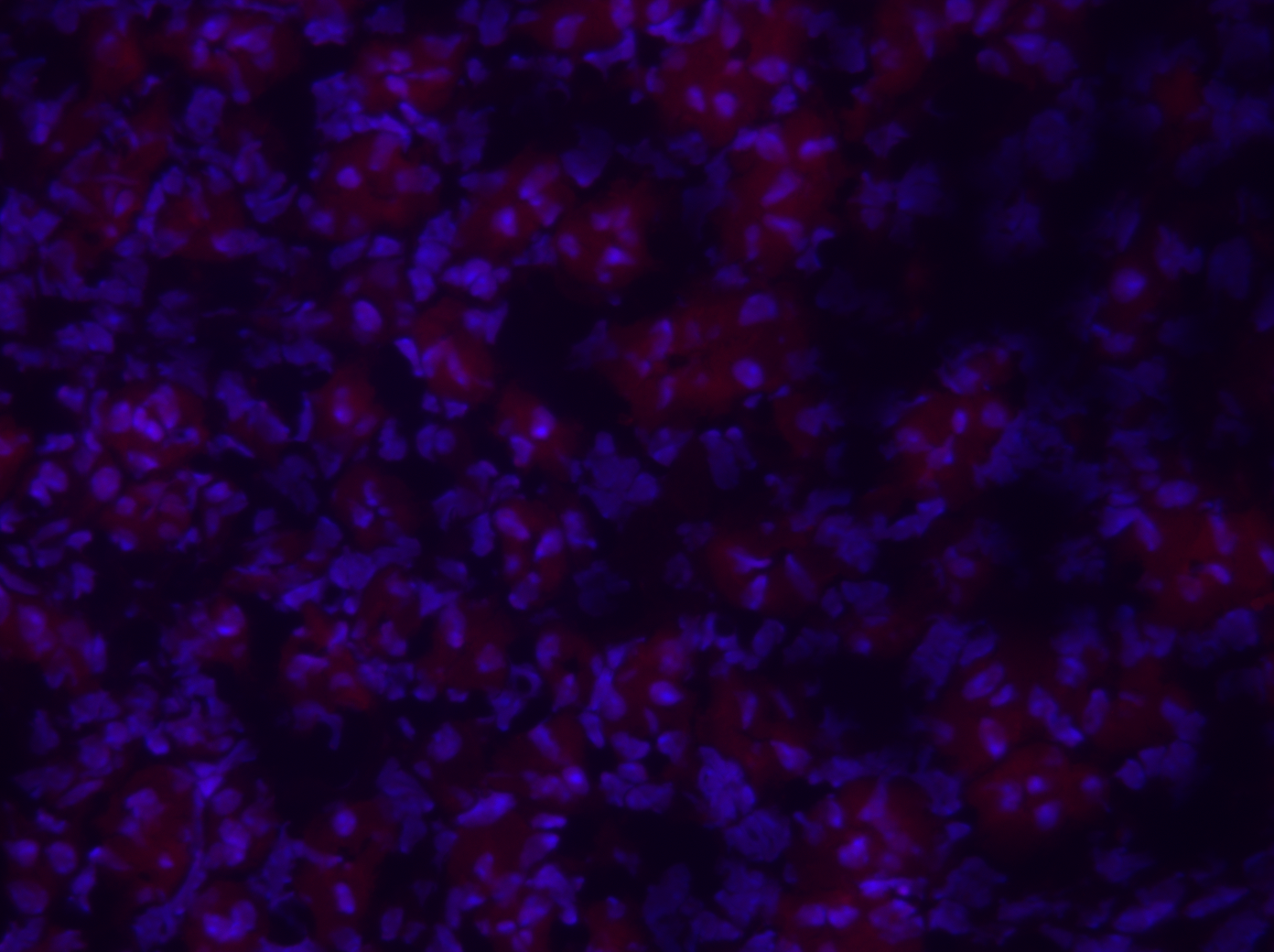

Supplement: Supplementary file 5 — Source data Fig. 3 [file 44321_2024_167_MOESM5_ESM.zip › EMM-2024-20280_Source data for Figure 3/3E/WT+Saline Merge.tif]

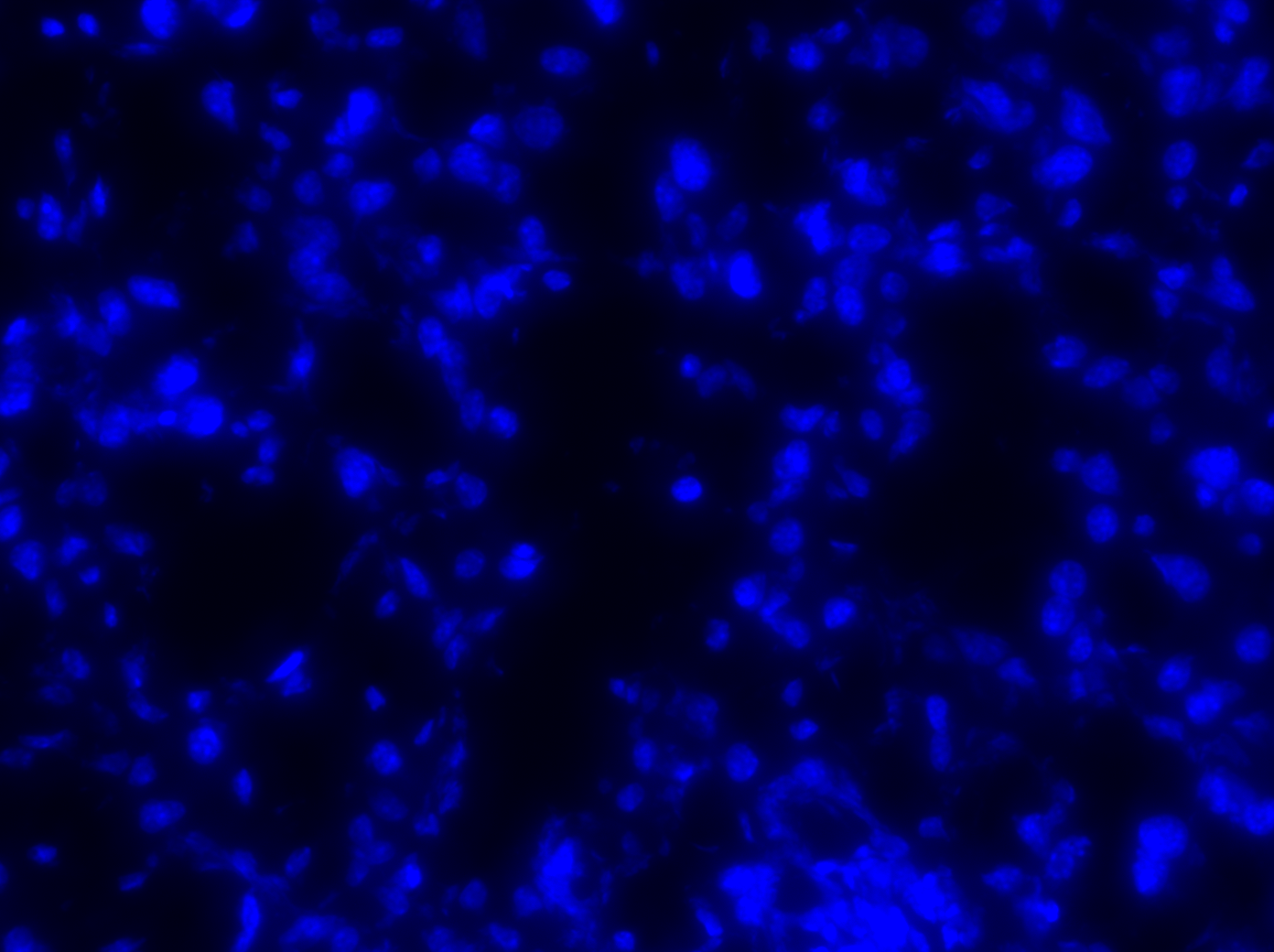

Supplement: Supplementary file 5 — Source data Fig. 3 [file 44321_2024_167_MOESM5_ESM.zip › EMM-2024-20280_Source data for Figure 3/3F/ADPKD+MMPP DAPI.tif]

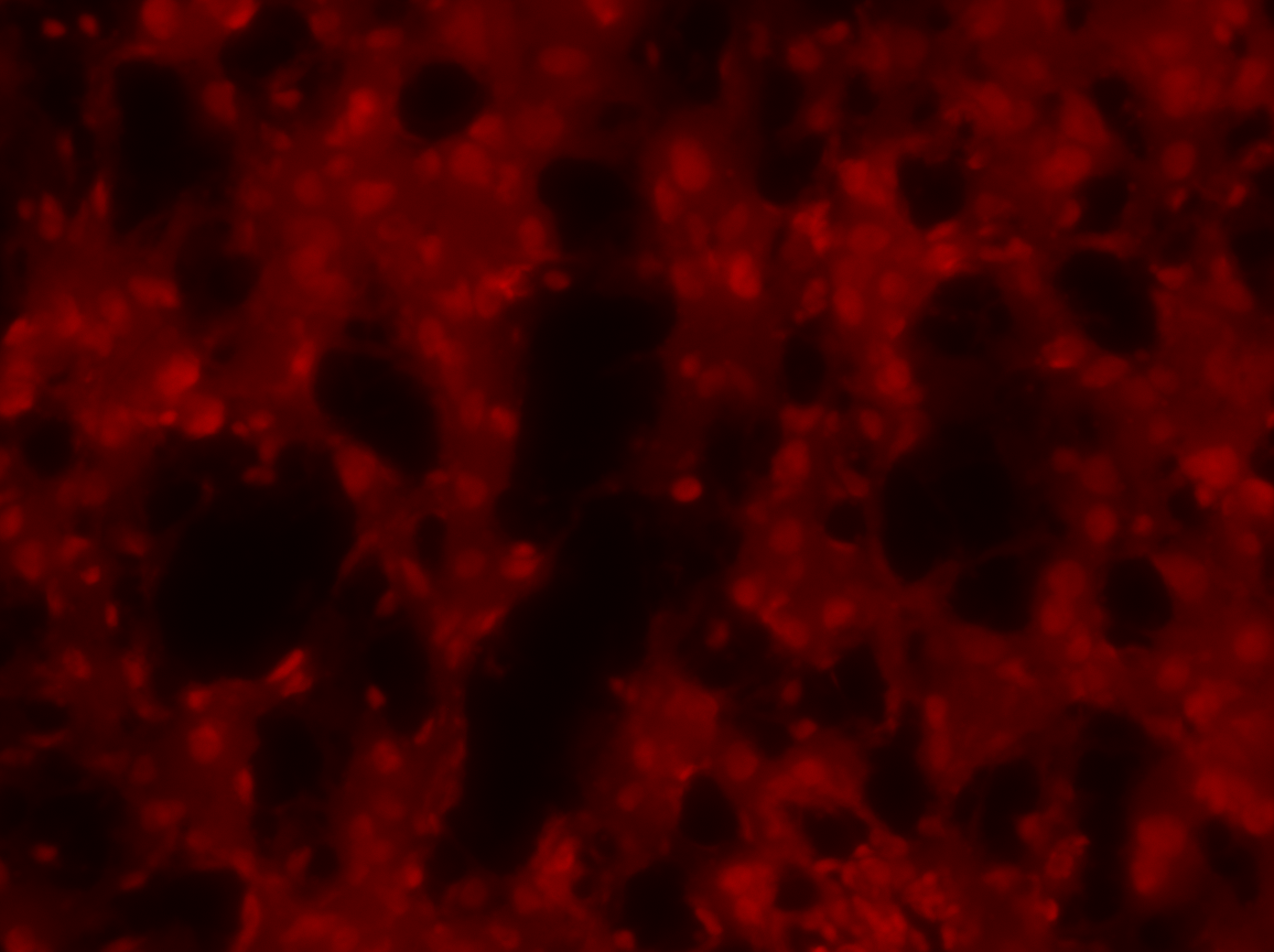

Supplement: Supplementary file 5 — Source data Fig. 3 [file 44321_2024_167_MOESM5_ESM.zip › EMM-2024-20280_Source data for Figure 3/3F/ADPKD+MMPP DHE.tif]

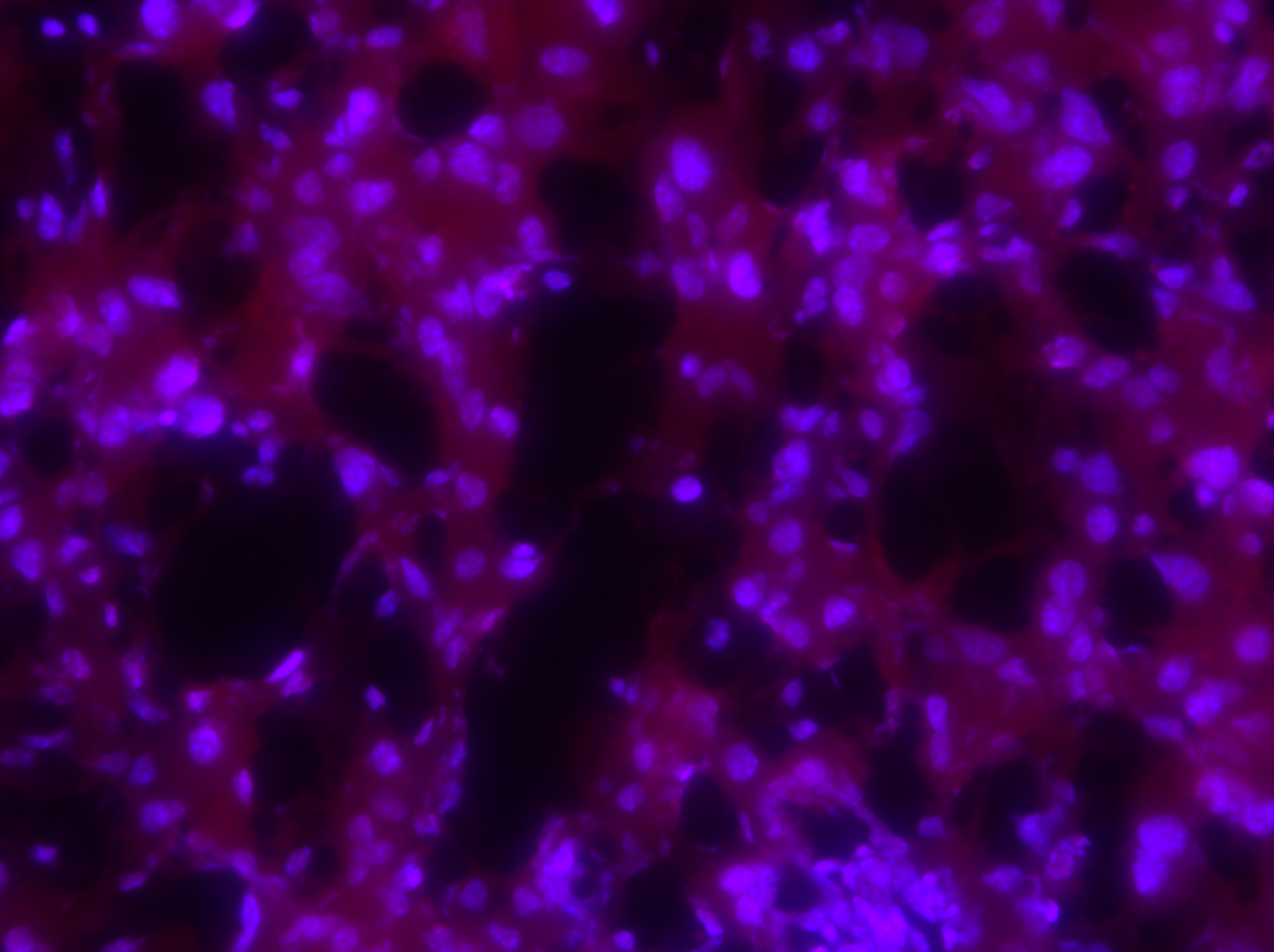

Supplement: Supplementary file 5 — Source data Fig. 3 [file 44321_2024_167_MOESM5_ESM.zip › EMM-2024-20280_Source data for Figure 3/3F/ADPKD+MMPP Merge.tif]

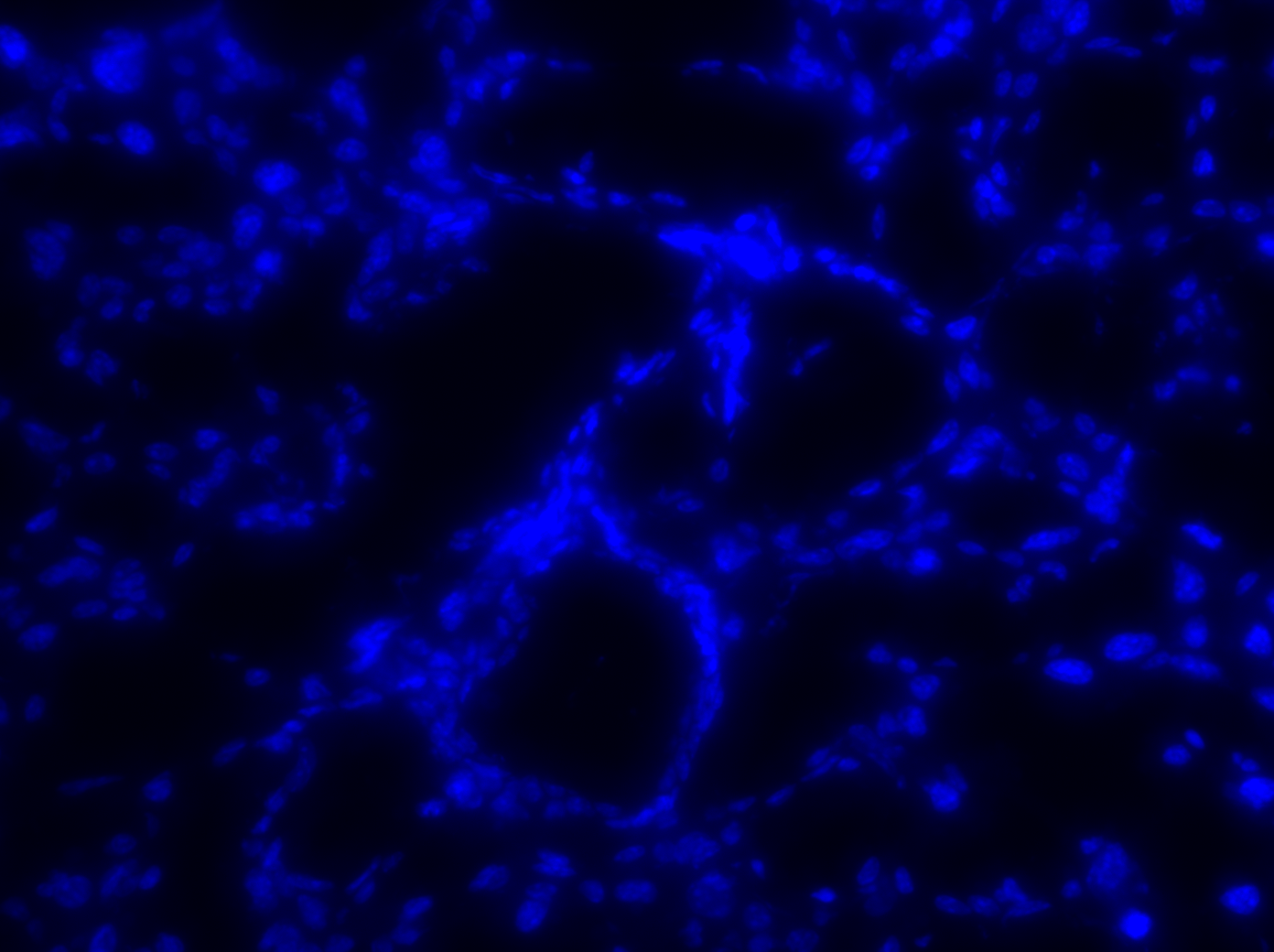

Supplement: Supplementary file 5 — Source data Fig. 3 [file 44321_2024_167_MOESM5_ESM.zip › EMM-2024-20280_Source data for Figure 3/3F/ADPKD+Saline DAPI.tif]

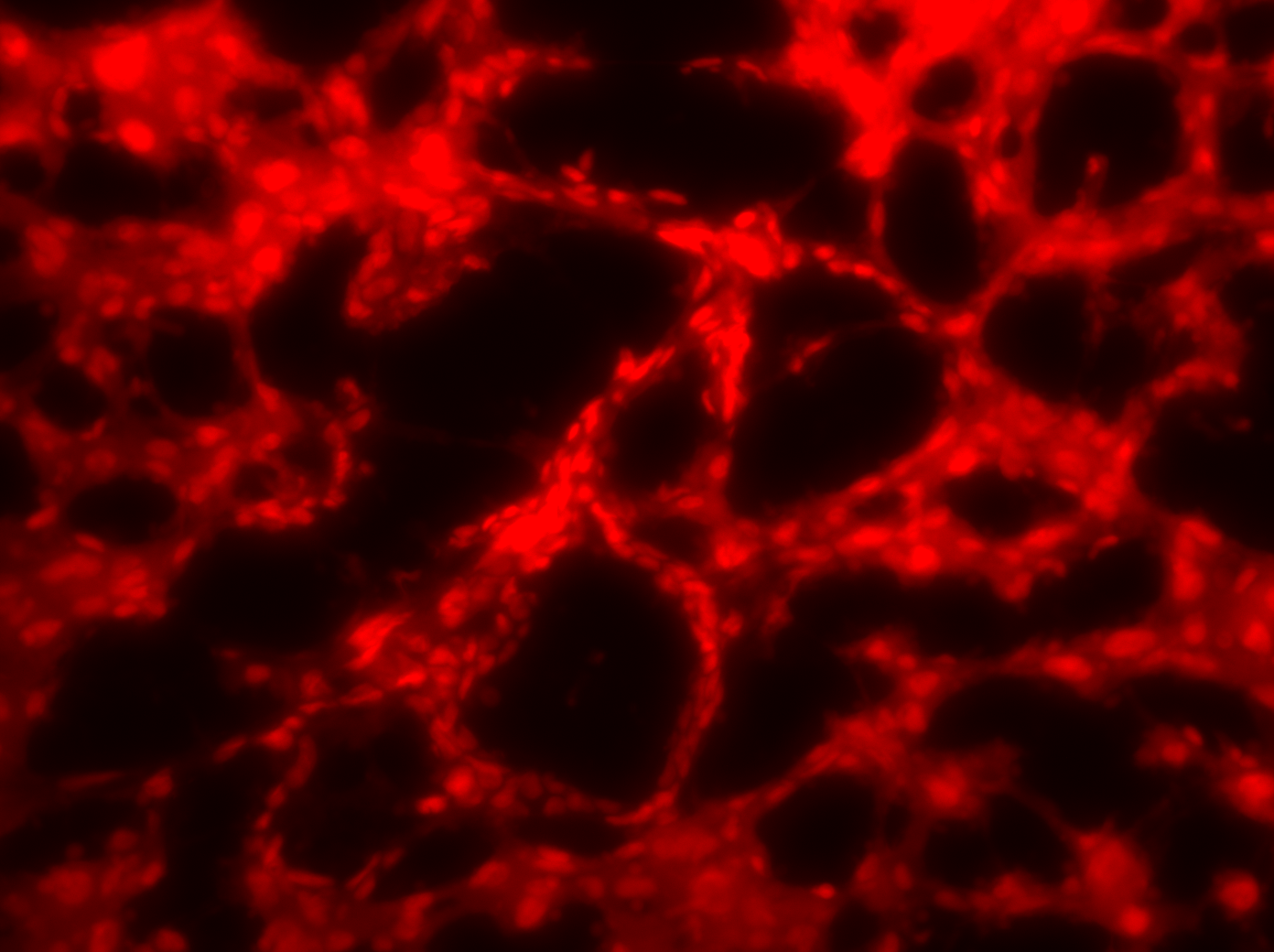

Supplement: Supplementary file 5 — Source data Fig. 3 [file 44321_2024_167_MOESM5_ESM.zip › EMM-2024-20280_Source data for Figure 3/3F/ADPKD+Saline DHE.tif]

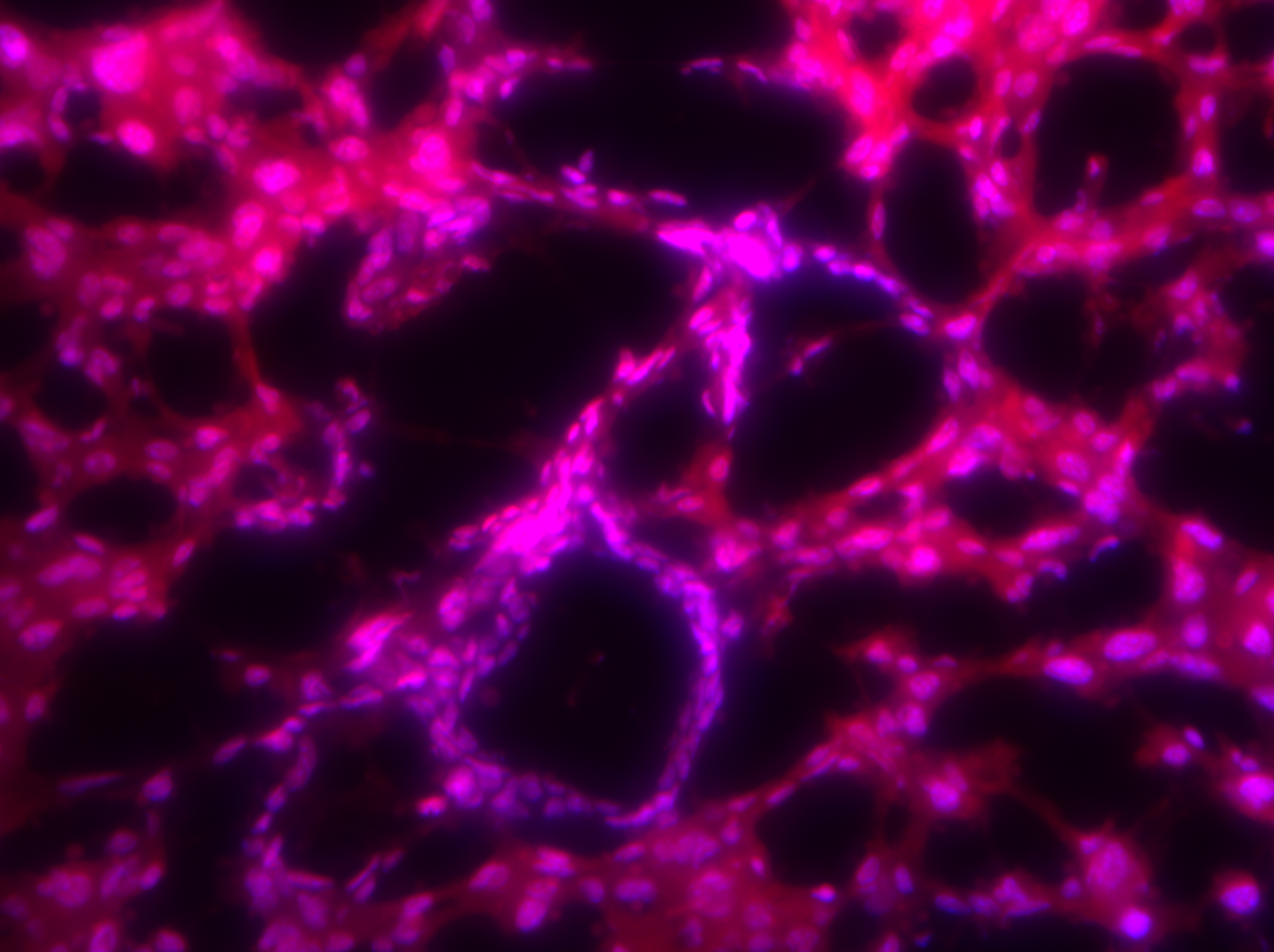

Supplement: Supplementary file 5 — Source data Fig. 3 [file 44321_2024_167_MOESM5_ESM.zip › EMM-2024-20280_Source data for Figure 3/3F/ADPKD+Saline Merge.tif]

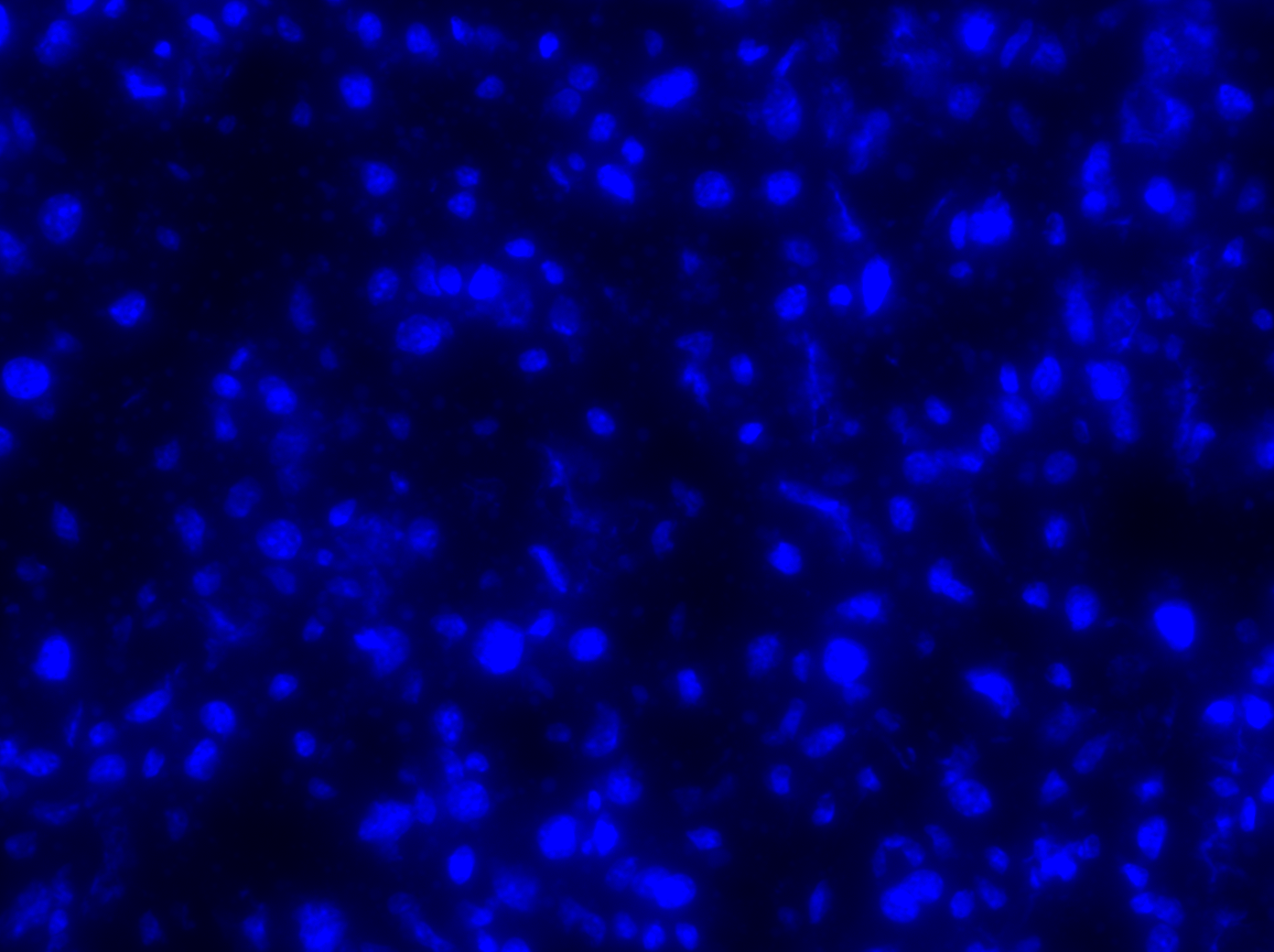

Supplement: Supplementary file 5 — Source data Fig. 3 [file 44321_2024_167_MOESM5_ESM.zip › EMM-2024-20280_Source data for Figure 3/3F/WT+Saline DAPI.tif]

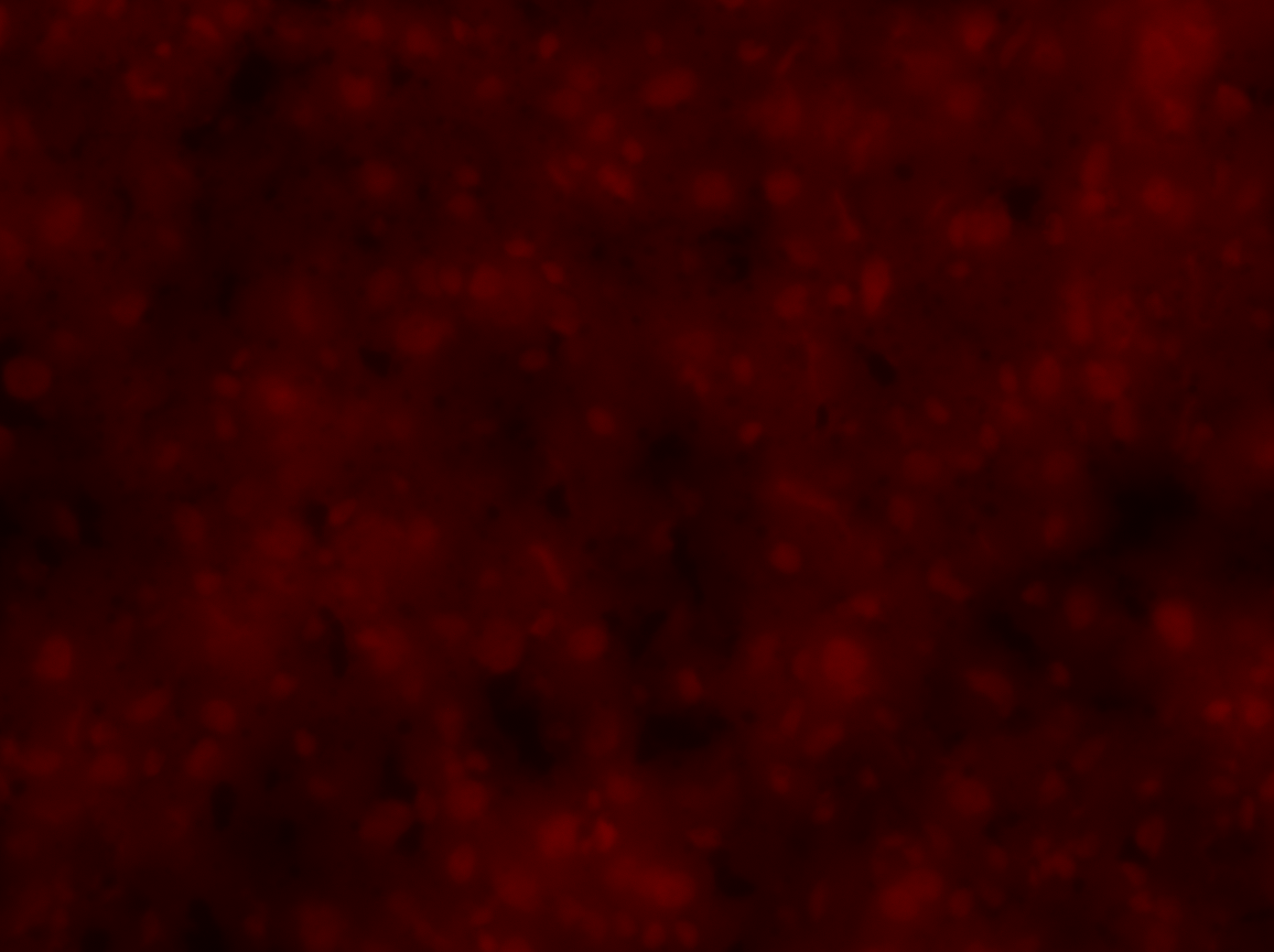

Supplement: Supplementary file 5 — Source data Fig. 3 [file 44321_2024_167_MOESM5_ESM.zip › EMM-2024-20280_Source data for Figure 3/3F/WT+Saline DHE.tif]

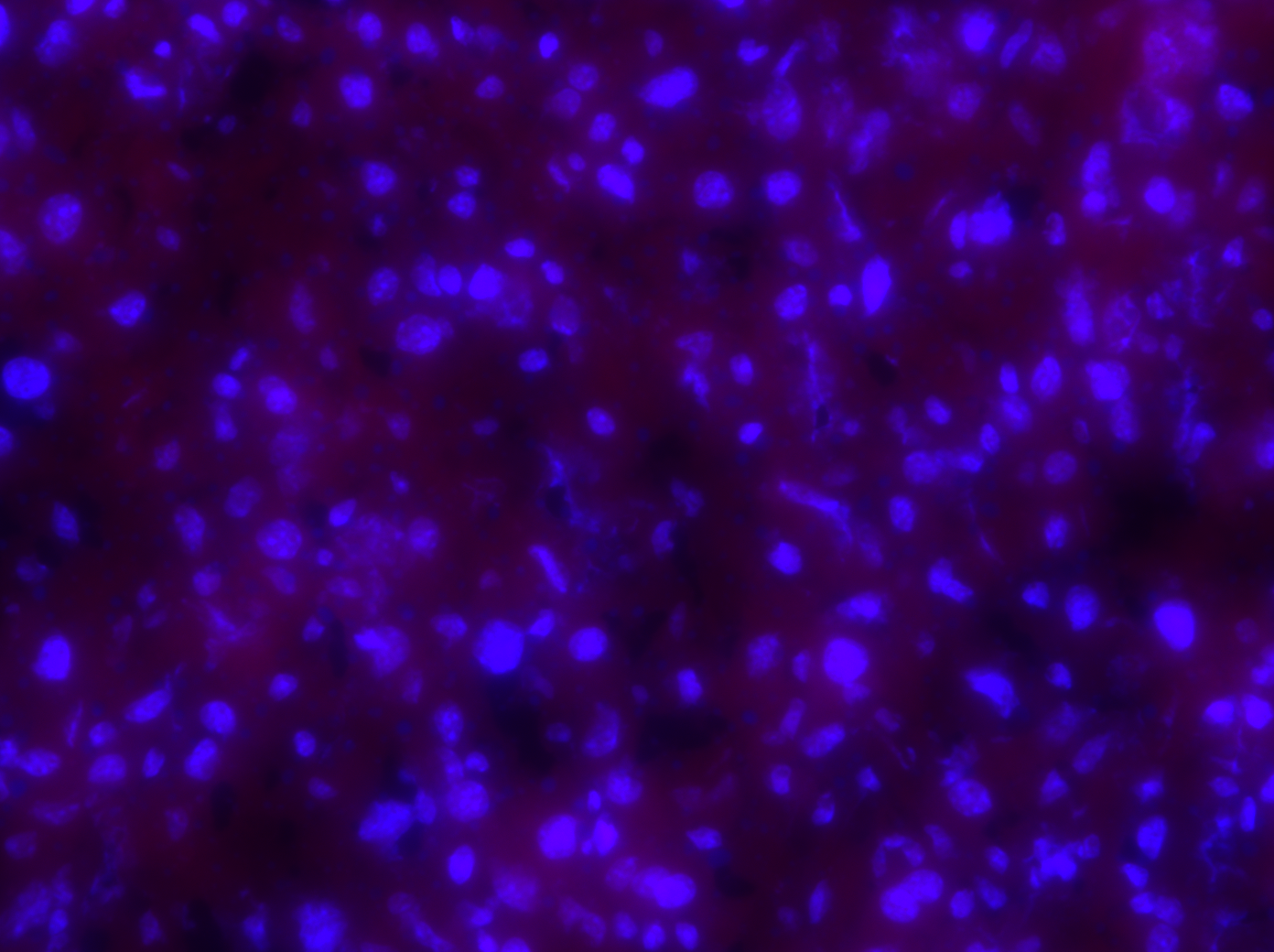

Supplement: Supplementary file 5 — Source data Fig. 3 [file 44321_2024_167_MOESM5_ESM.zip › EMM-2024-20280_Source data for Figure 3/3F/WT+Saline Merge.tif]

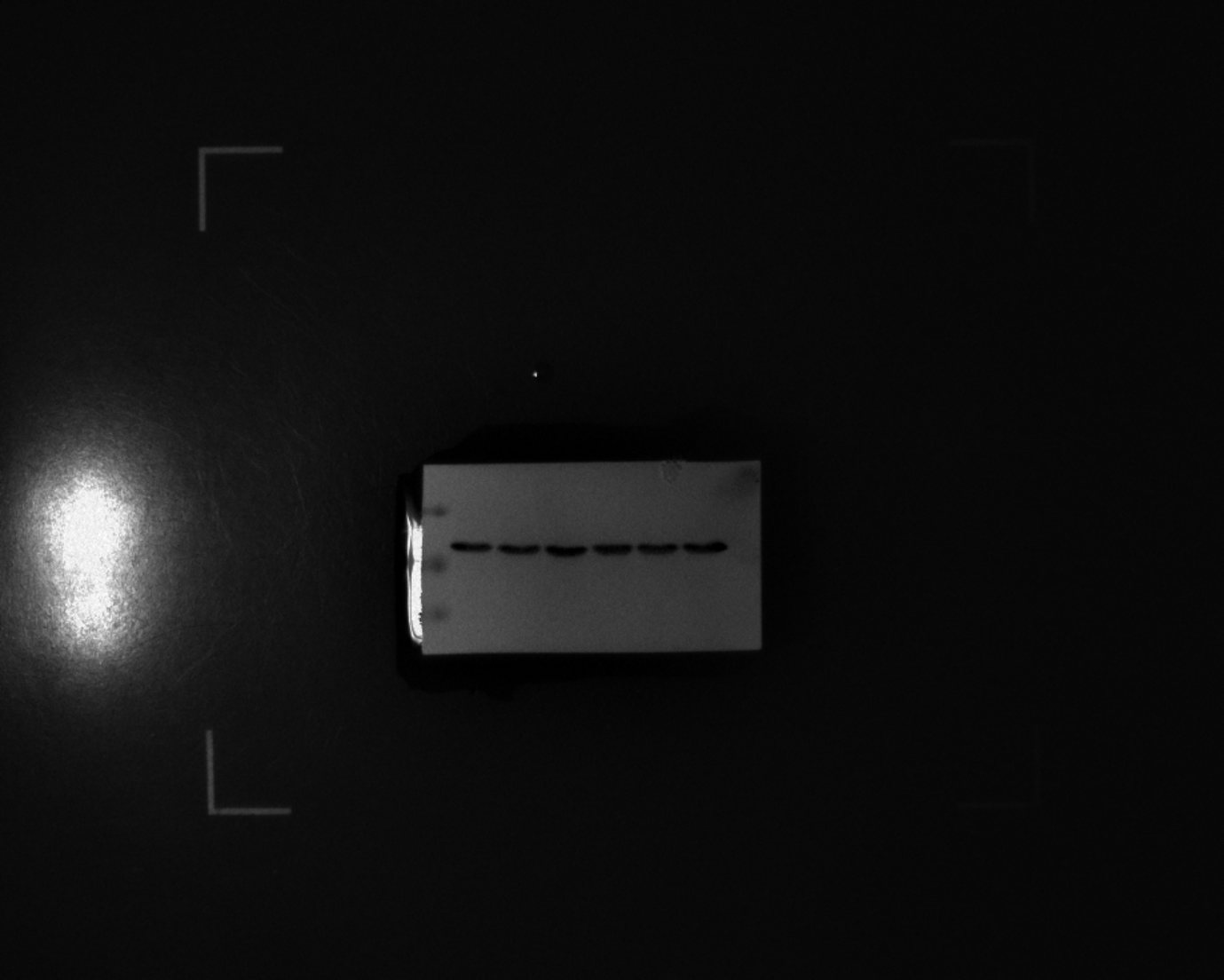

Supplement: Supplementary file 7 — Source data Fig. 5 [file 44321_2024_167_MOESM7_ESM.zip › EMM-2024-20280_Source data for Figure 5/5C/GAPDH.tif]

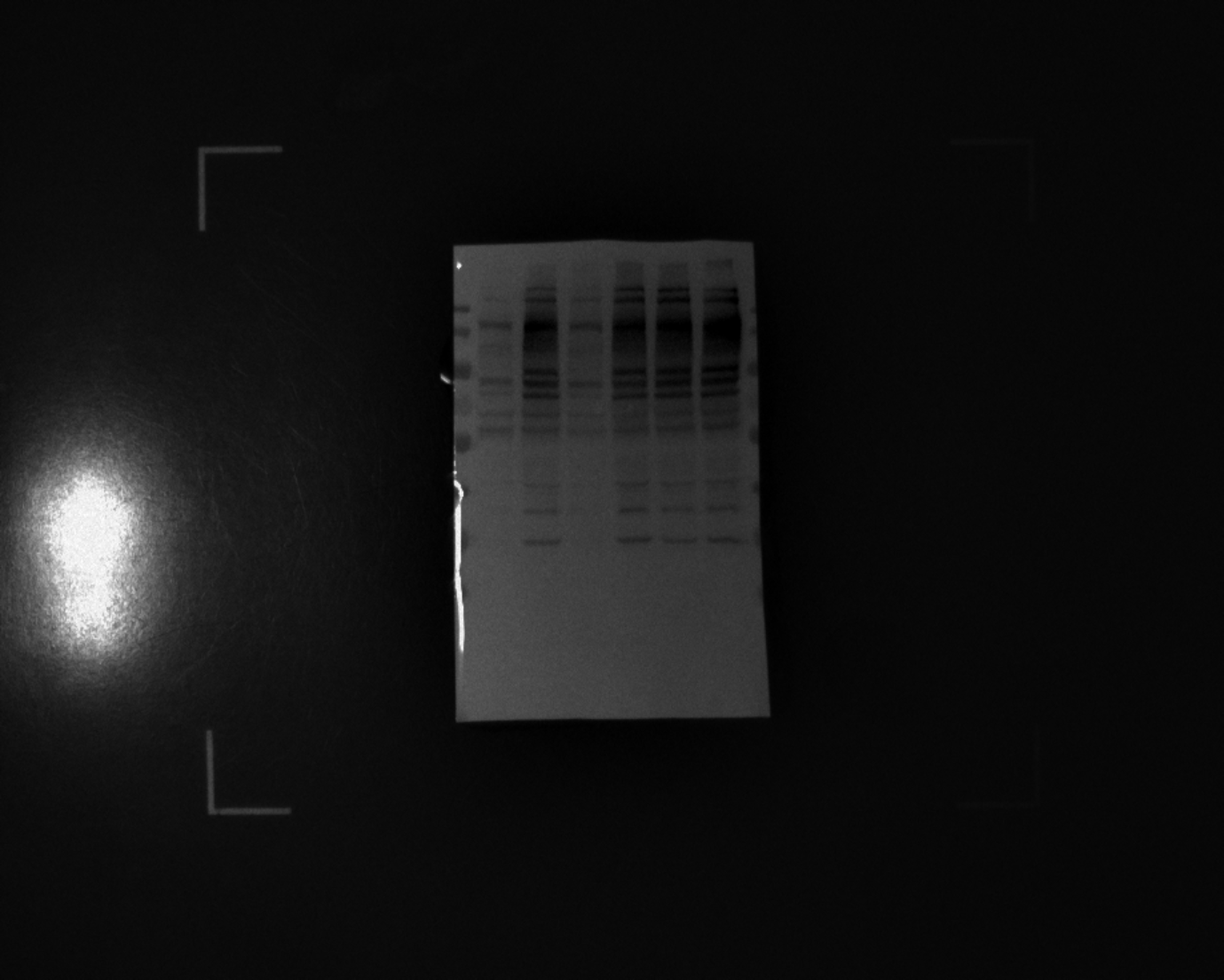

Supplement: Supplementary file 7 — Source data Fig. 5 [file 44321_2024_167_MOESM7_ESM.zip › EMM-2024-20280_Source data for Figure 5/5C/p-PKA.tif]

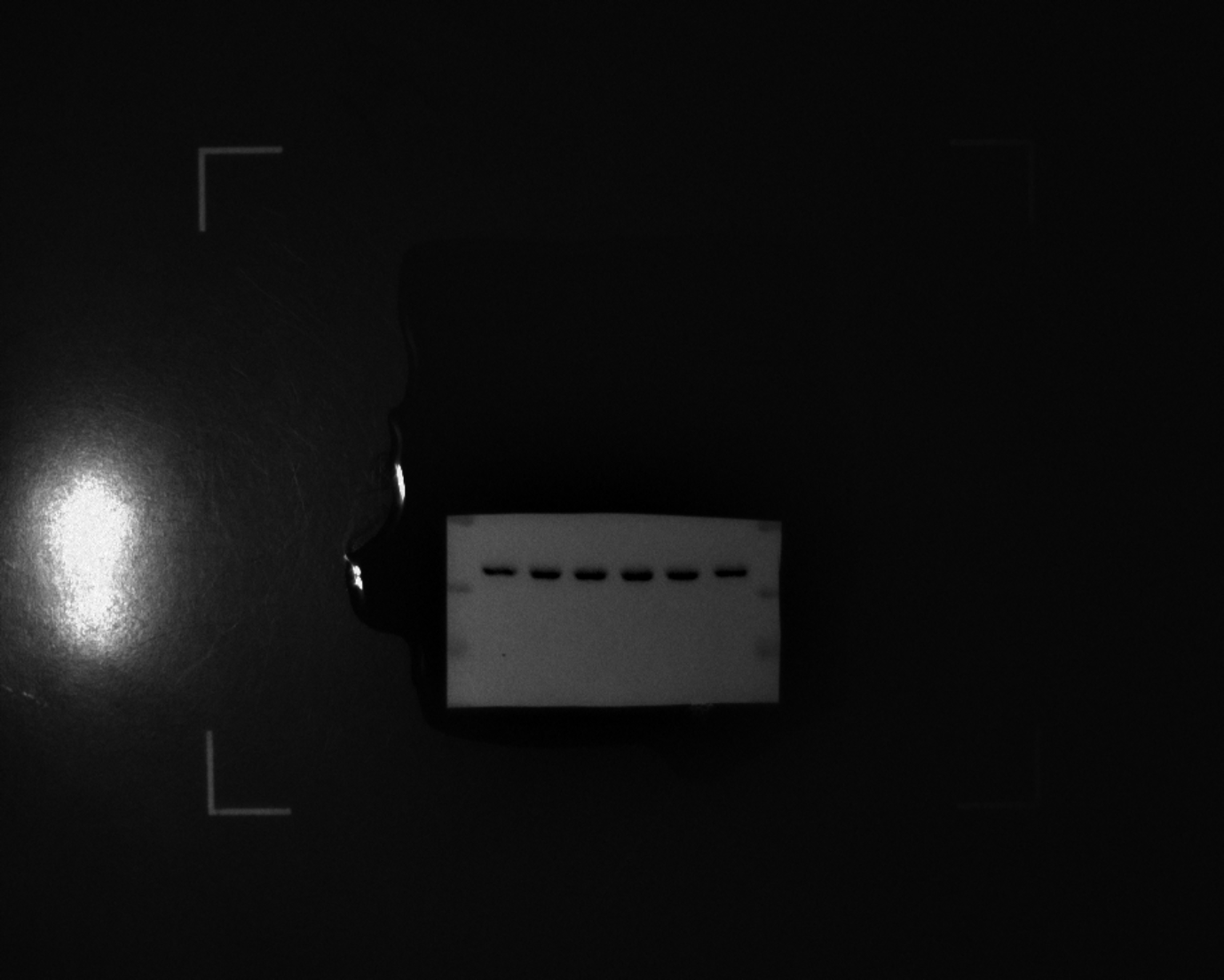

Supplement: Supplementary file 7 — Source data Fig. 5 [file 44321_2024_167_MOESM7_ESM.zip › EMM-2024-20280_Source data for Figure 5/5D/CREB.tif]

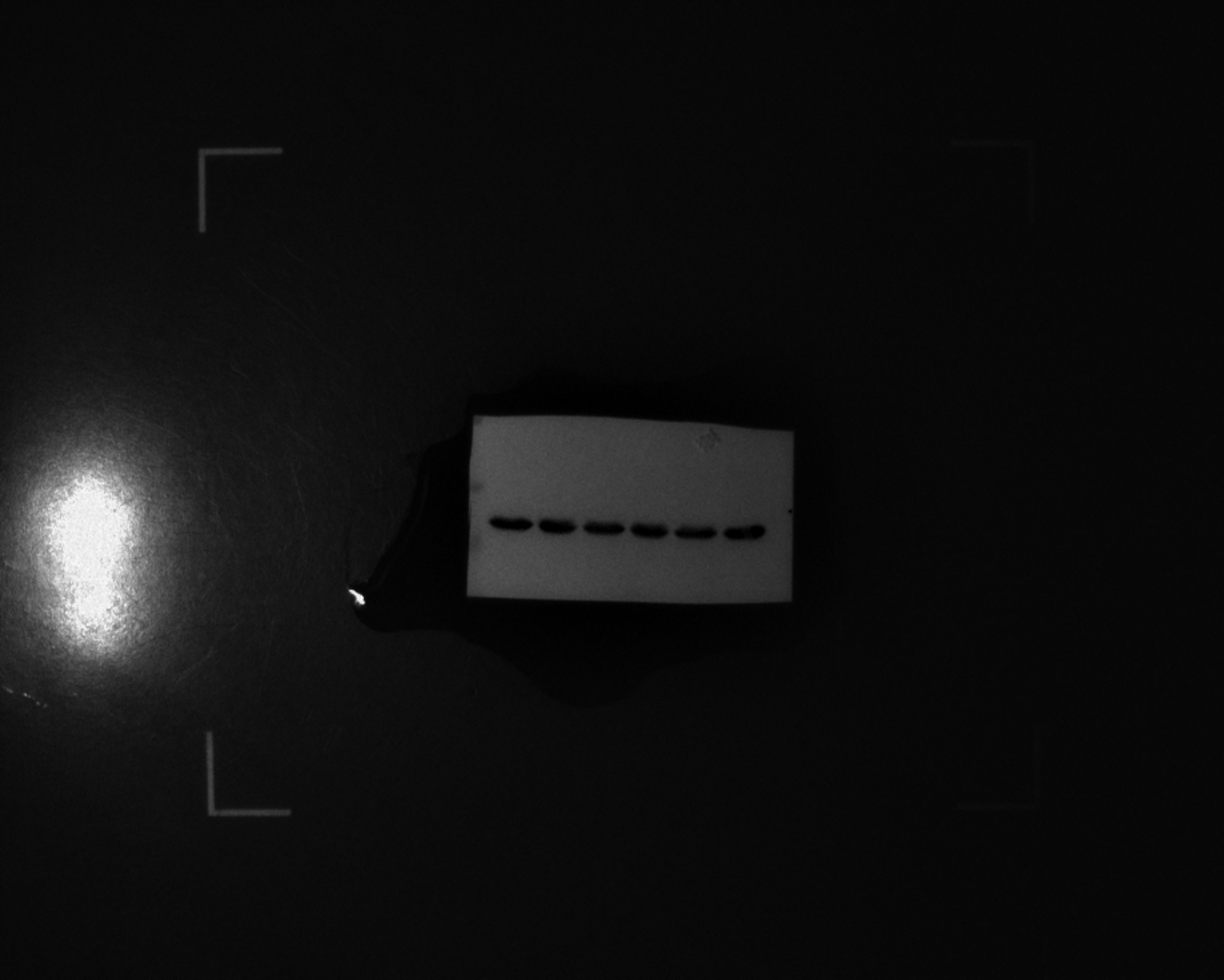

Supplement: Supplementary file 7 — Source data Fig. 5 [file 44321_2024_167_MOESM7_ESM.zip › EMM-2024-20280_Source data for Figure 5/5D/GAPDH.tif]

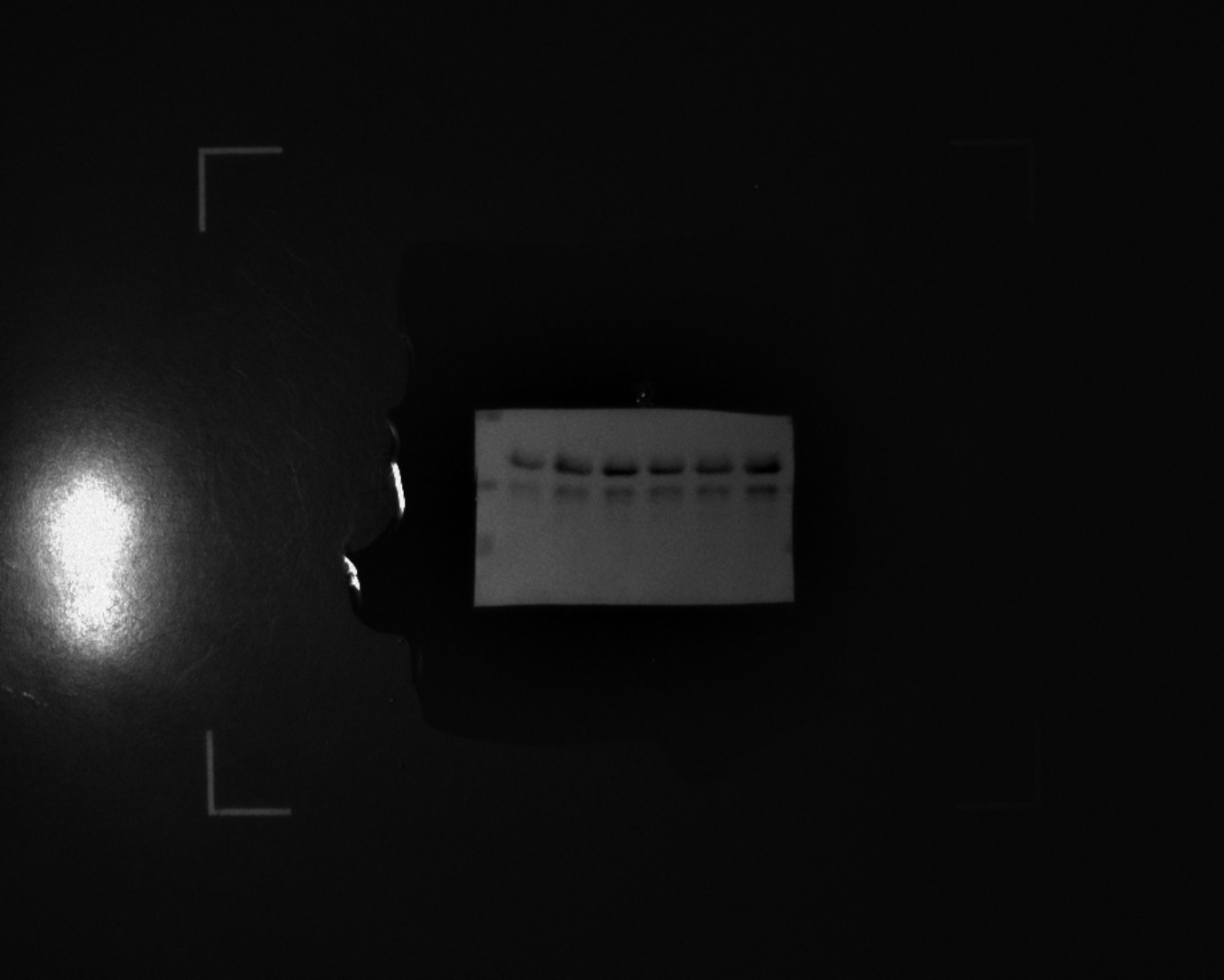

Supplement: Supplementary file 7 — Source data Fig. 5 [file 44321_2024_167_MOESM7_ESM.zip › EMM-2024-20280_Source data for Figure 5/5D/P-CREB.tif]

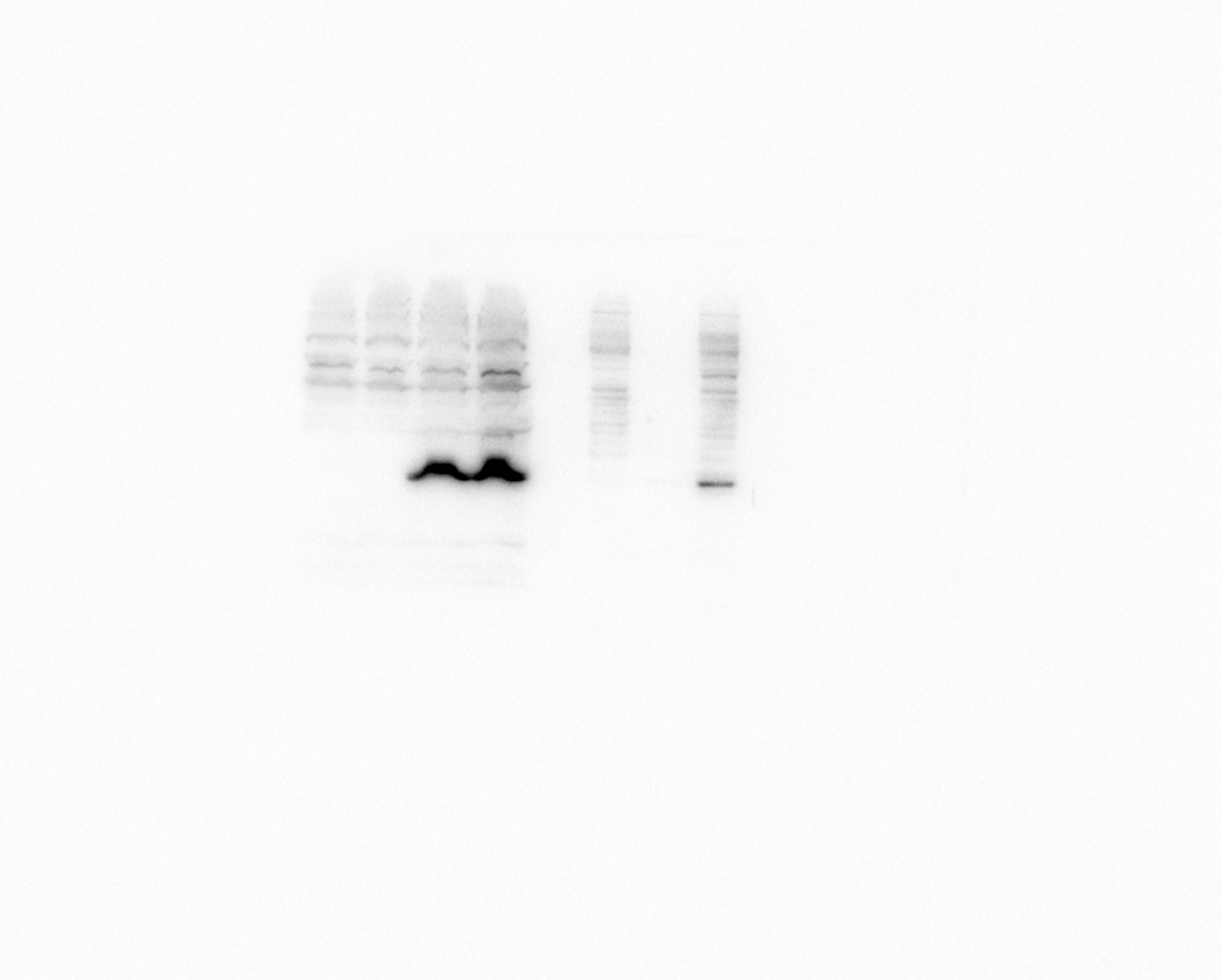

Supplement: Supplementary file 7 — Source data Fig. 5 [file 44321_2024_167_MOESM7_ESM.zip › EMM-2024-20280_Source data for Figure 5/5F/FLAG Input.Tif]

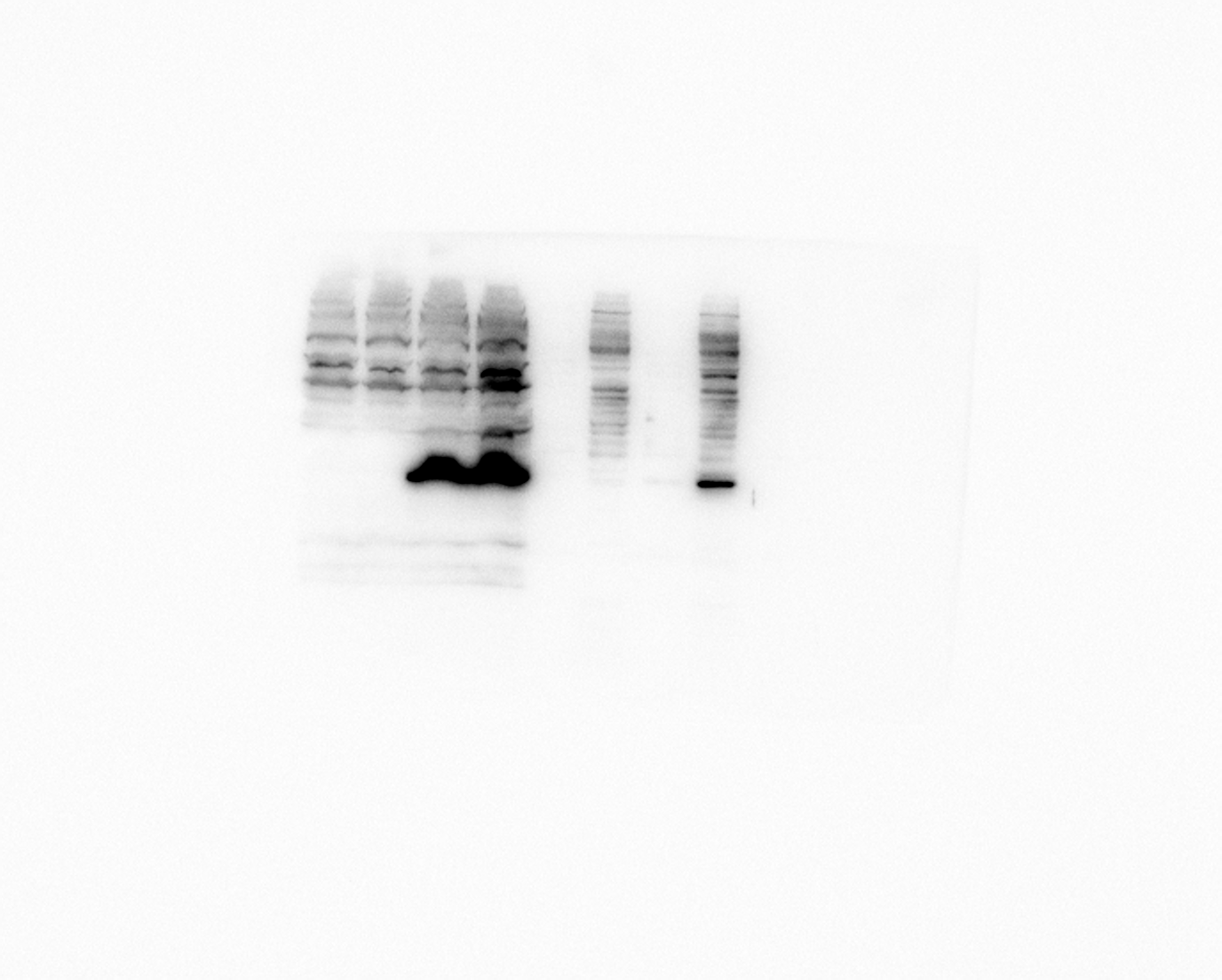

Supplement: Supplementary file 7 — Source data Fig. 5 [file 44321_2024_167_MOESM7_ESM.zip › EMM-2024-20280_Source data for Figure 5/5F/FLAG pull down.Tif]

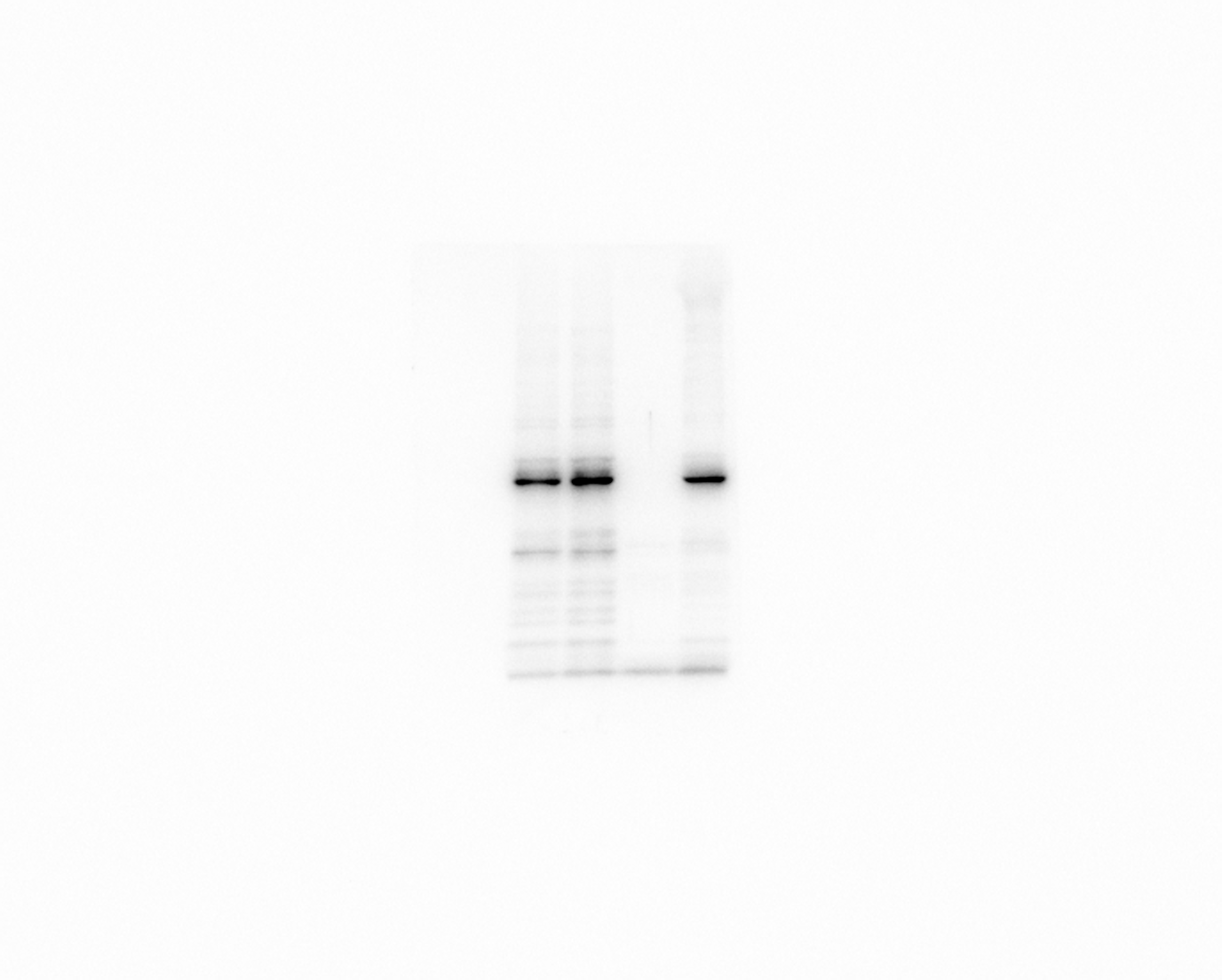

Supplement: Supplementary file 7 — Source data Fig. 5 [file 44321_2024_167_MOESM7_ESM.zip › EMM-2024-20280_Source data for Figure 5/5G/CREB line1-2 Input.Tif]

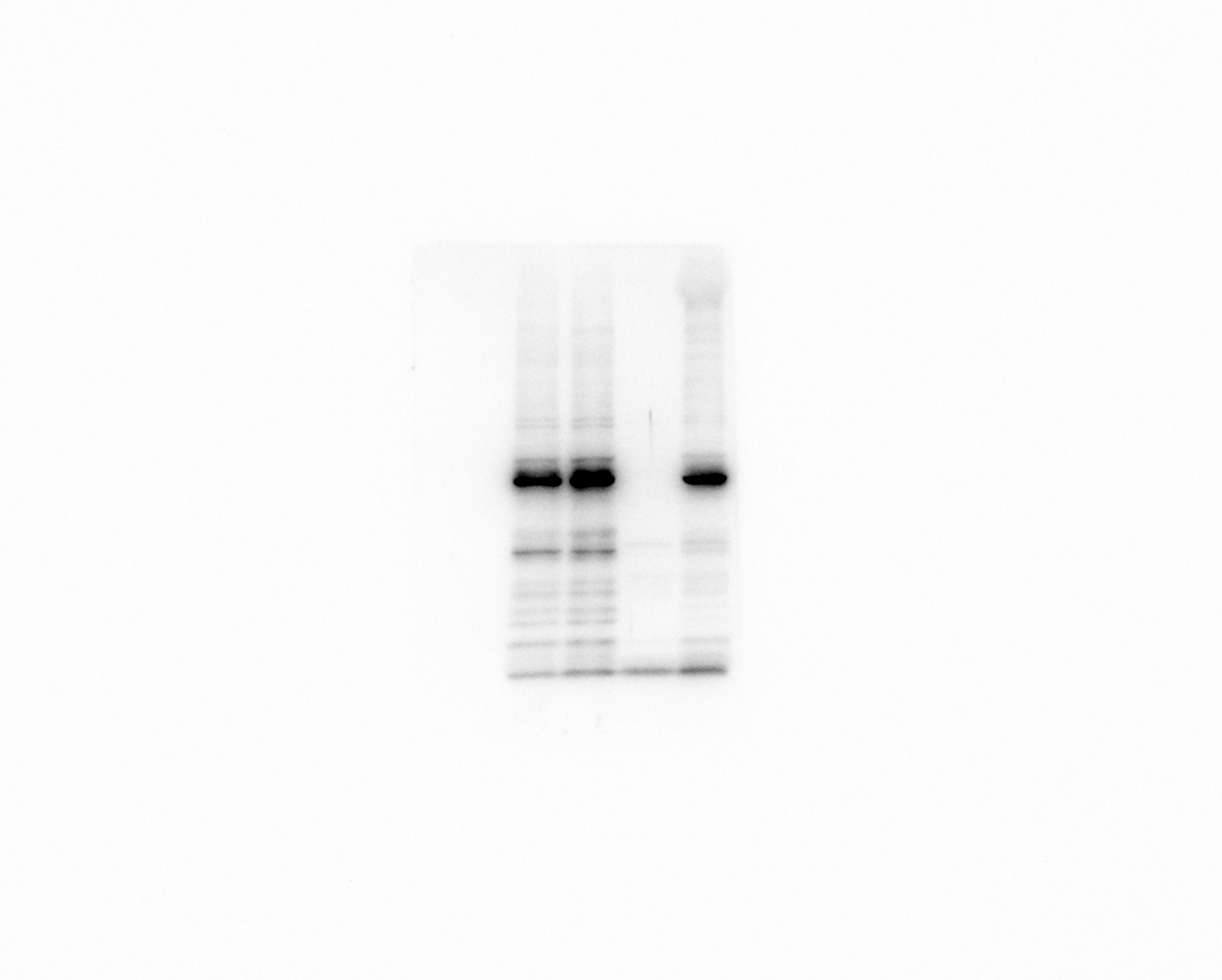

Supplement: Supplementary file 7 — Source data Fig. 5 [file 44321_2024_167_MOESM7_ESM.zip › EMM-2024-20280_Source data for Figure 5/5G/CREB line3-4 pull down.Tif]

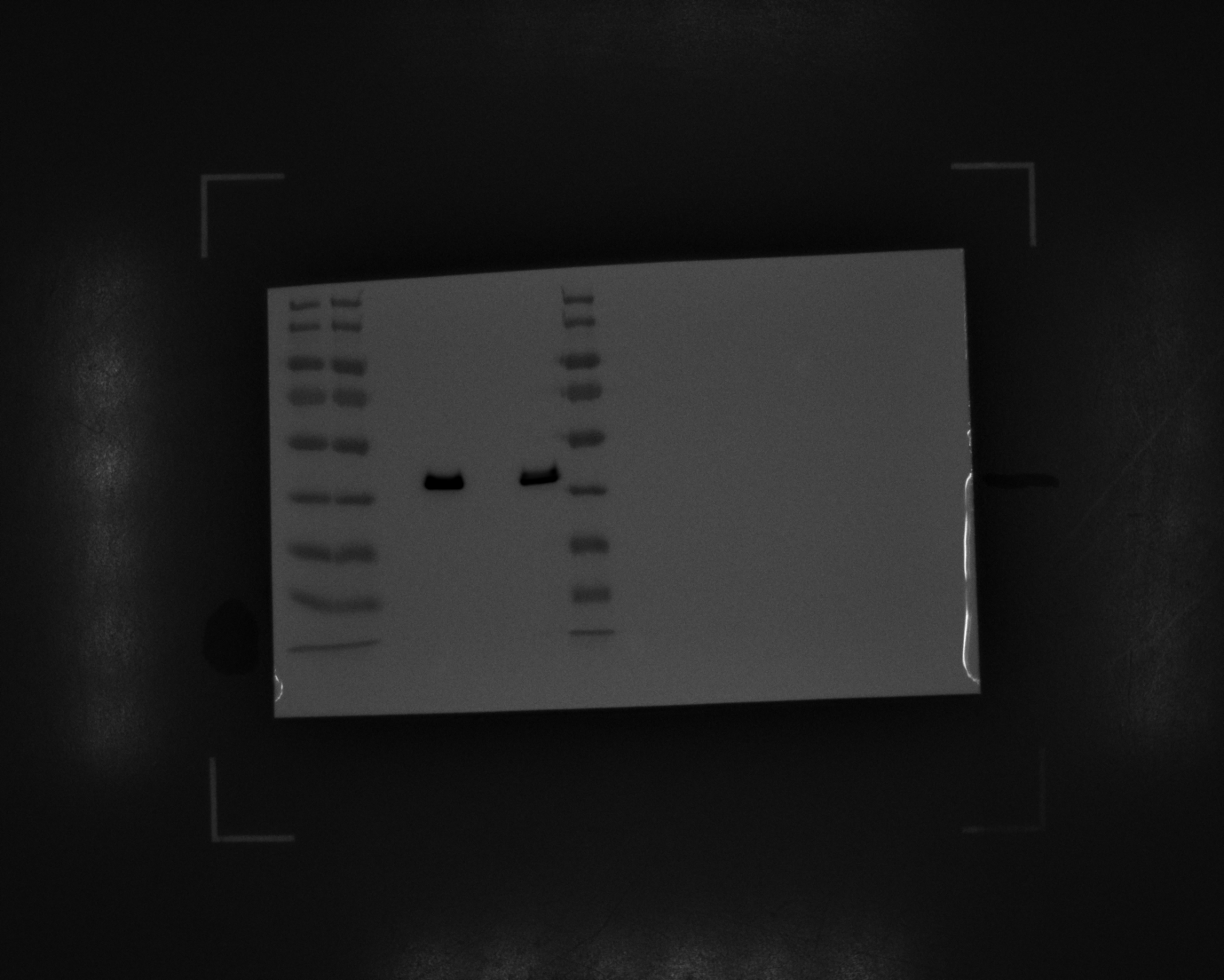

Supplement: Supplementary file 7 — Source data Fig. 5 [file 44321_2024_167_MOESM7_ESM.zip › EMM-2024-20280_Source data for Figure 5/5H/CREB Input.tif]

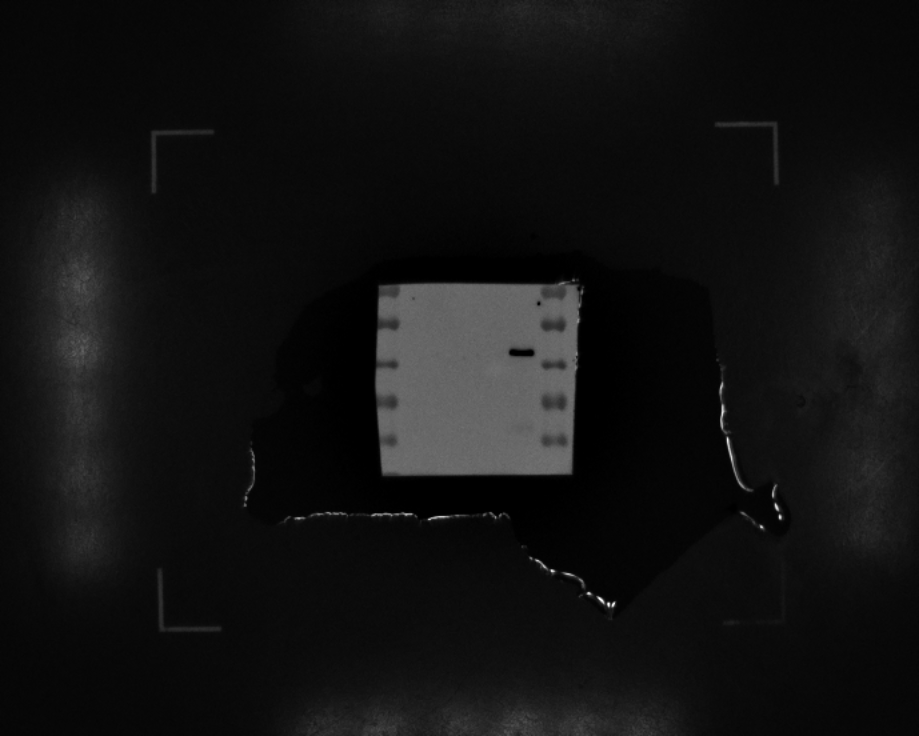

Supplement: Supplementary file 7 — Source data Fig. 5 [file 44321_2024_167_MOESM7_ESM.zip › EMM-2024-20280_Source data for Figure 5/5H/CREB pull down.tif]

Figure 5C

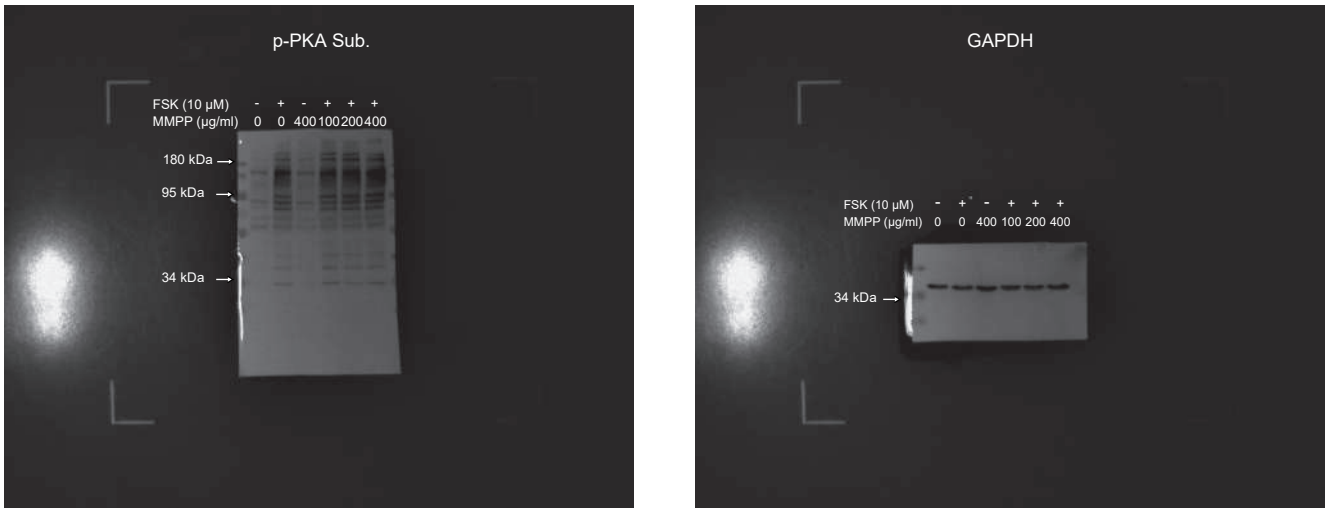

Figure 5D

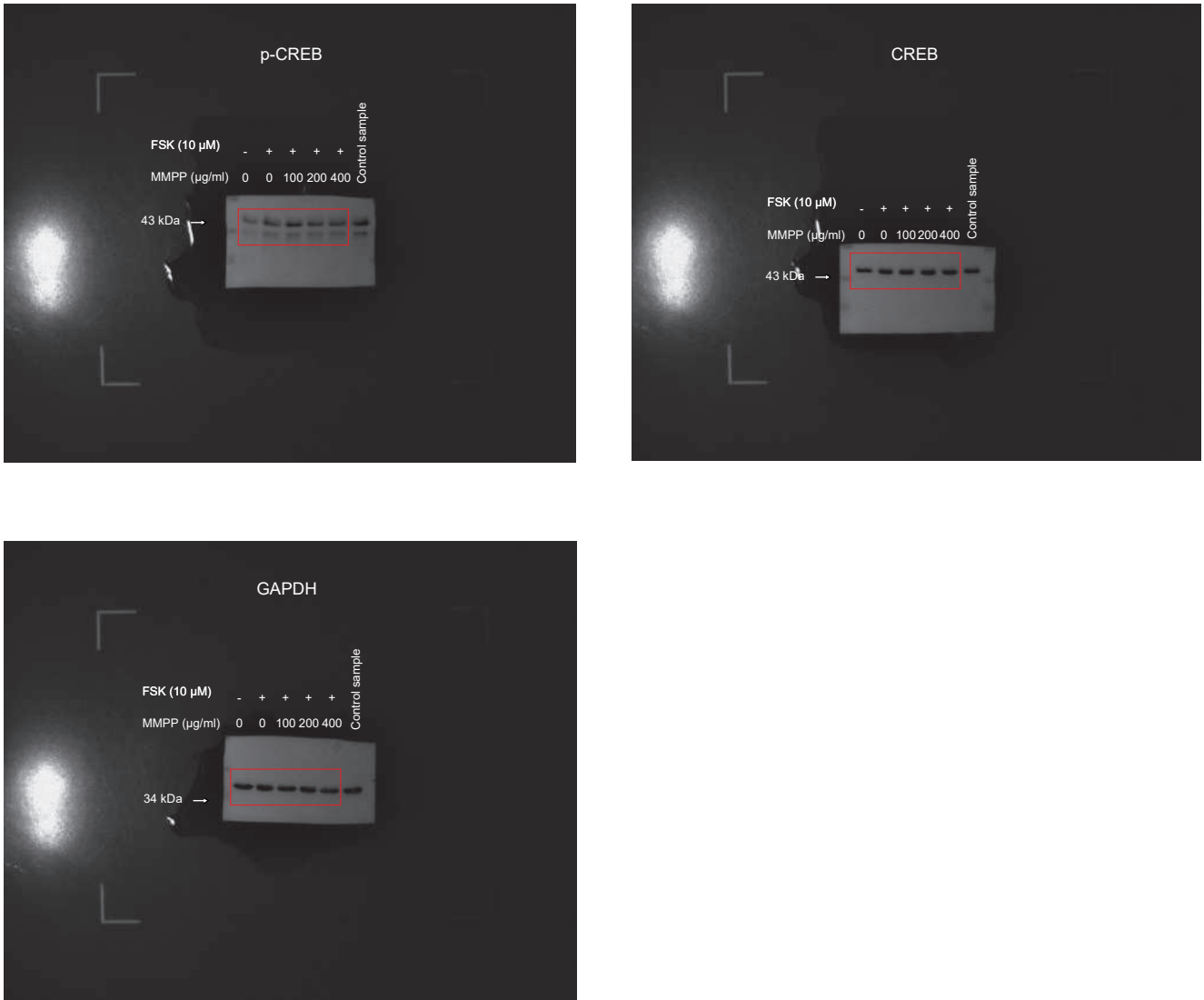

Figure 5F

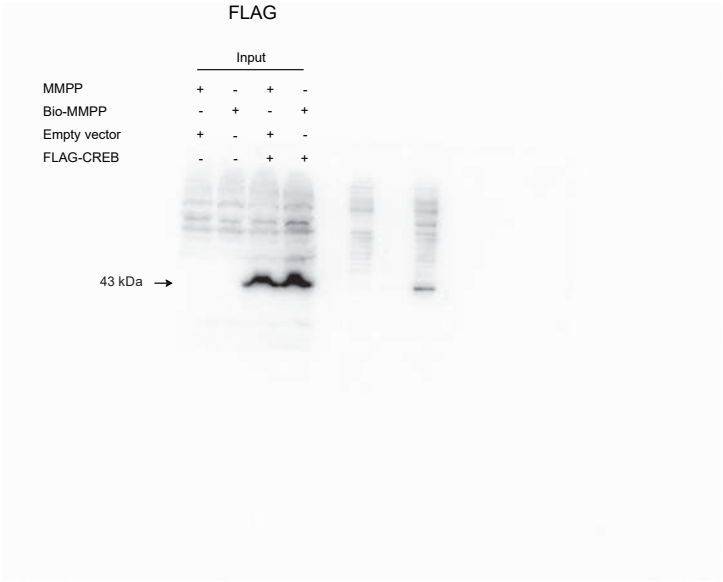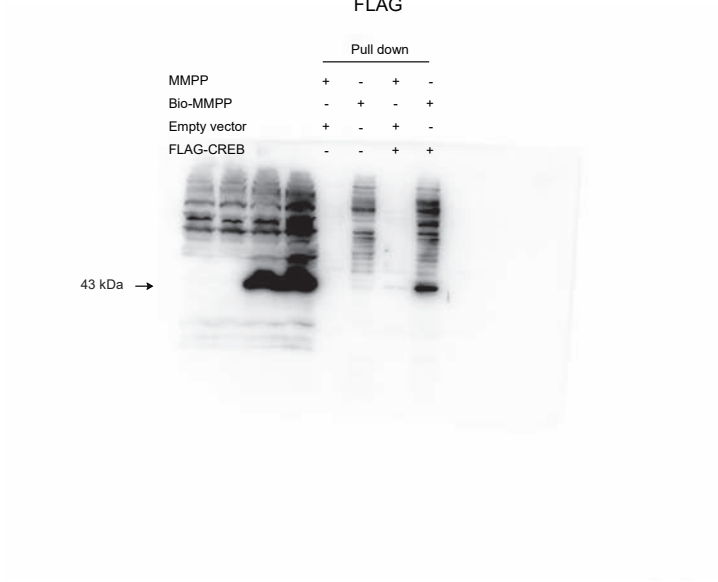

Figure 5G

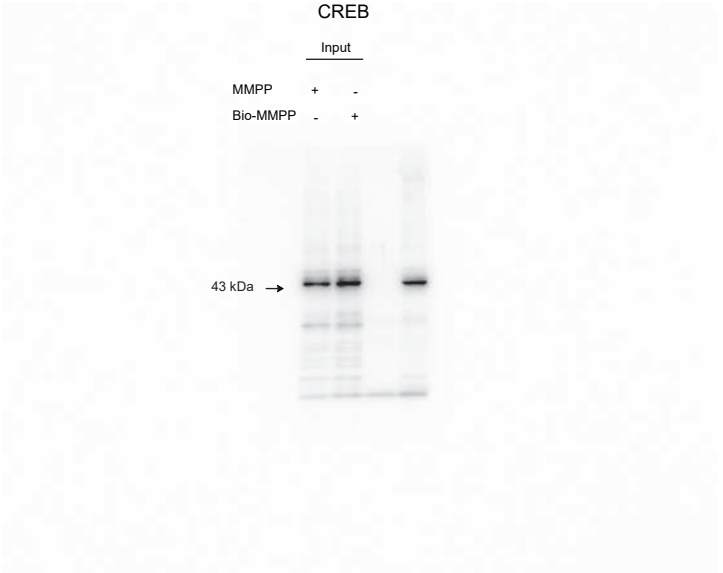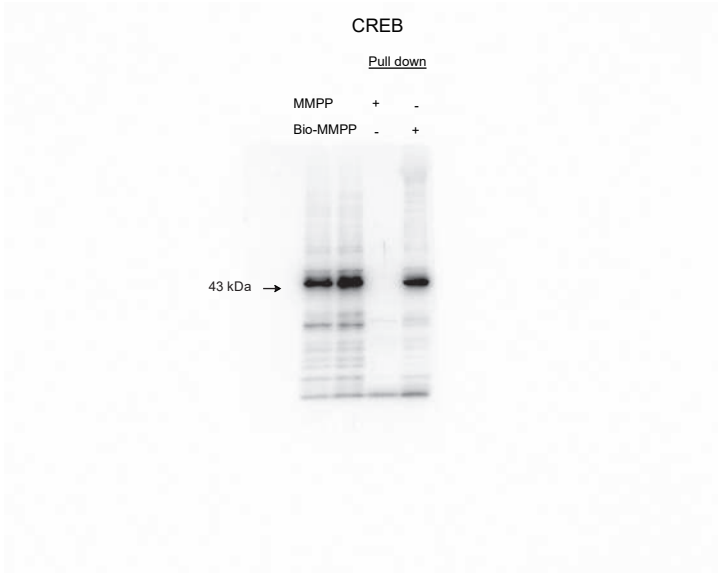

Figure 5H

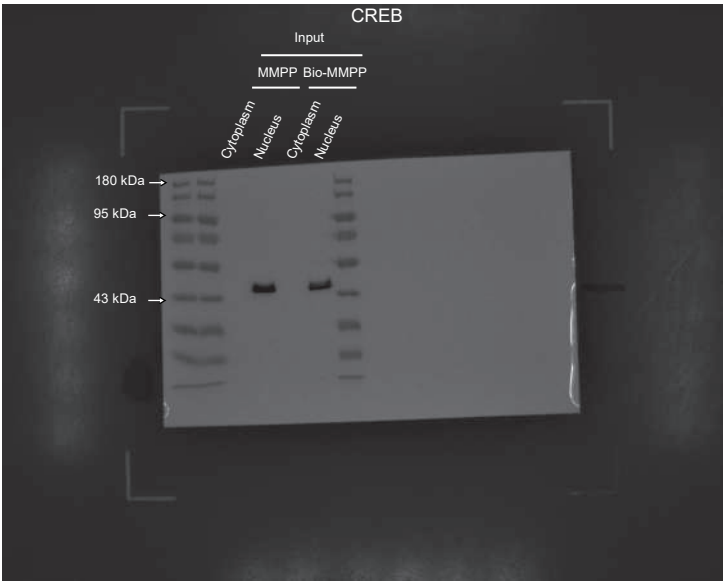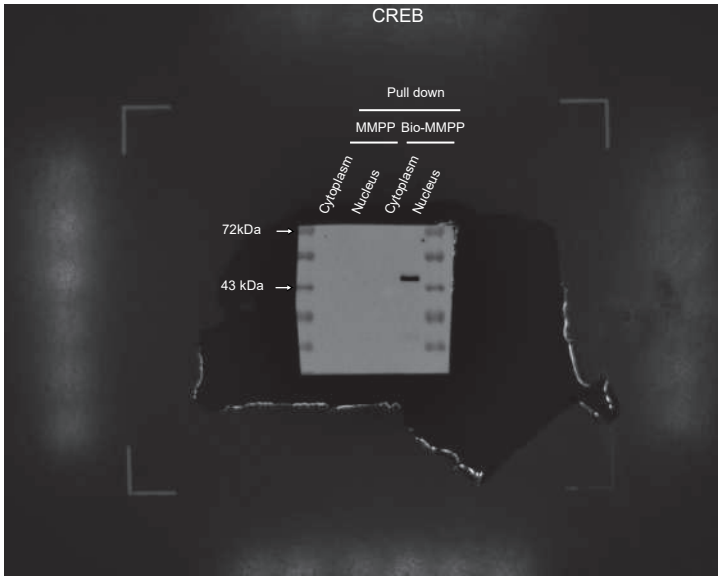

Supplement: Supplementary file 7 — Source data Fig. 5 [file 44321_2024_167_MOESM7_ESM.zip › EMM-2024-20280_Source data for Figure 5/README for gels.pdf]

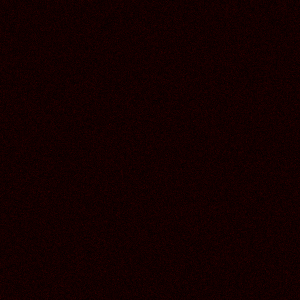

Supplement: Supplementary file 8 — Source data Fig. 6 [file 44321_2024_167_MOESM8_ESM.zip › EMM-2024-20280_Source data for Figure 6/6B/Cherry (Cherry+FITC-MMPP).tif]

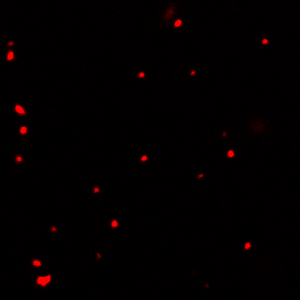

Supplement: Supplementary file 8 — Source data Fig. 6 [file 44321_2024_167_MOESM8_ESM.zip › EMM-2024-20280_Source data for Figure 6/6B/Cherry (Cherry-CREB+FITC-MMPP).tif]

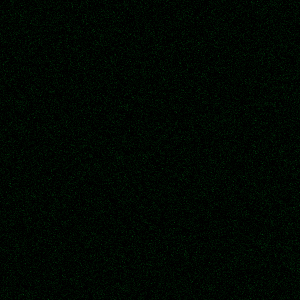

Supplement: Supplementary file 8 — Source data Fig. 6 [file 44321_2024_167_MOESM8_ESM.zip › EMM-2024-20280_Source data for Figure 6/6B/FITC (Cherry+FITC-MMPP).tif]

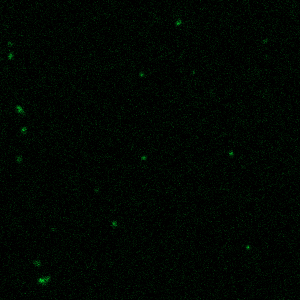

Supplement: Supplementary file 8 — Source data Fig. 6 [file 44321_2024_167_MOESM8_ESM.zip › EMM-2024-20280_Source data for Figure 6/6B/FITC (Cherry-CREB+FITC-MMPP).tif]

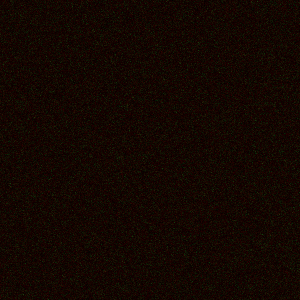

Supplement: Supplementary file 8 — Source data Fig. 6 [file 44321_2024_167_MOESM8_ESM.zip › EMM-2024-20280_Source data for Figure 6/6B/Merge (Cherry+FITC-MMPP).tif]

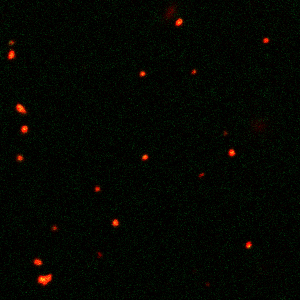

Supplement: Supplementary file 8 — Source data Fig. 6 [file 44321_2024_167_MOESM8_ESM.zip › EMM-2024-20280_Source data for Figure 6/6B/Merge (Cherry-CREB+FITC-MMPP).tif]

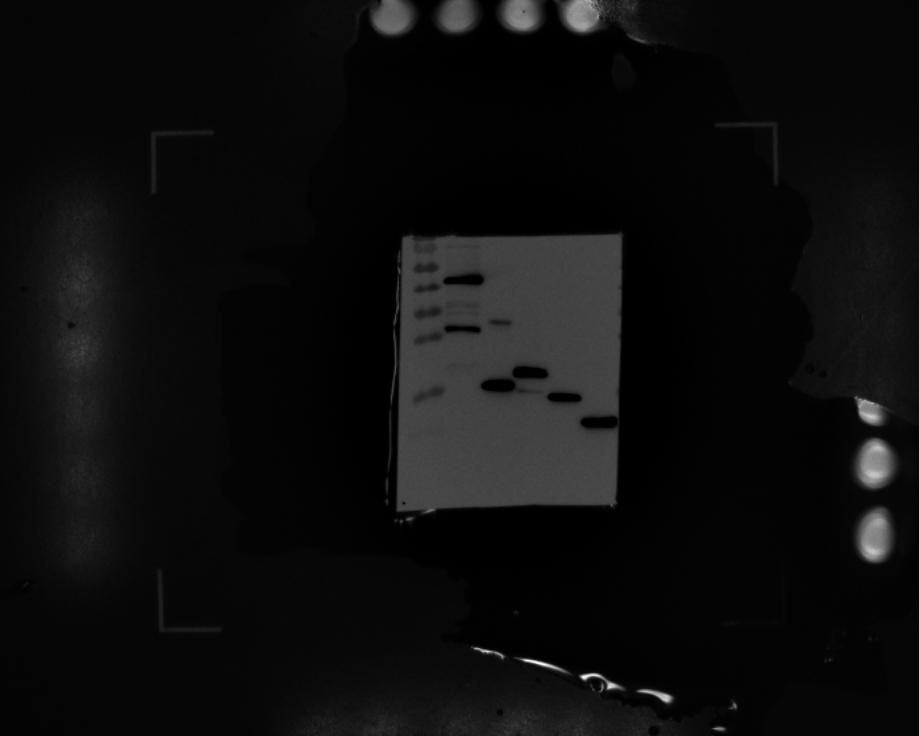

Supplement: Supplementary file 8 — Source data Fig. 6 [file 44321_2024_167_MOESM8_ESM.zip › EMM-2024-20280_Source data for Figure 6/6C/FLAG Input.tif]

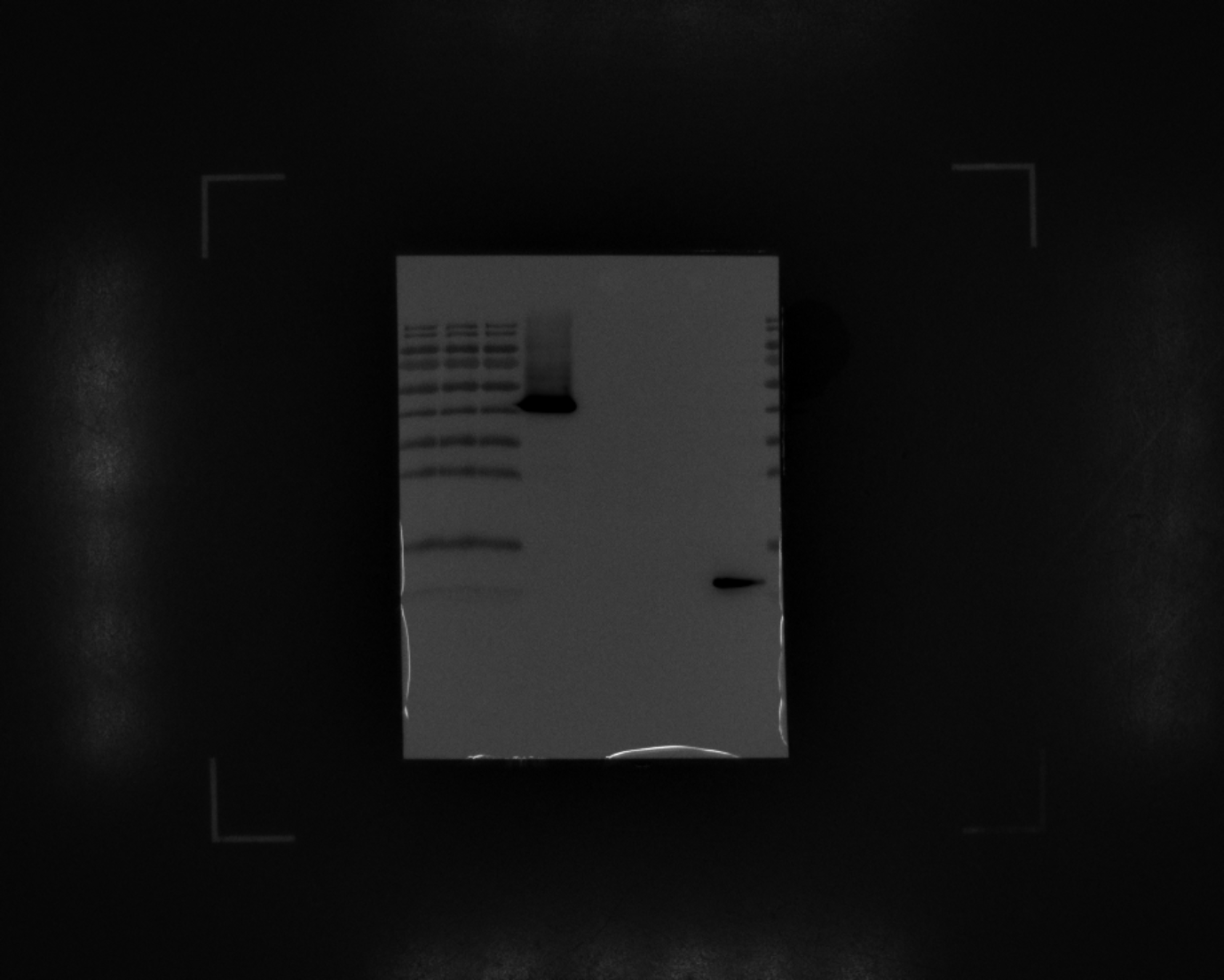

Supplement: Supplementary file 8 — Source data Fig. 6 [file 44321_2024_167_MOESM8_ESM.zip › EMM-2024-20280_Source data for Figure 6/6C/FLAG pull down.tif]

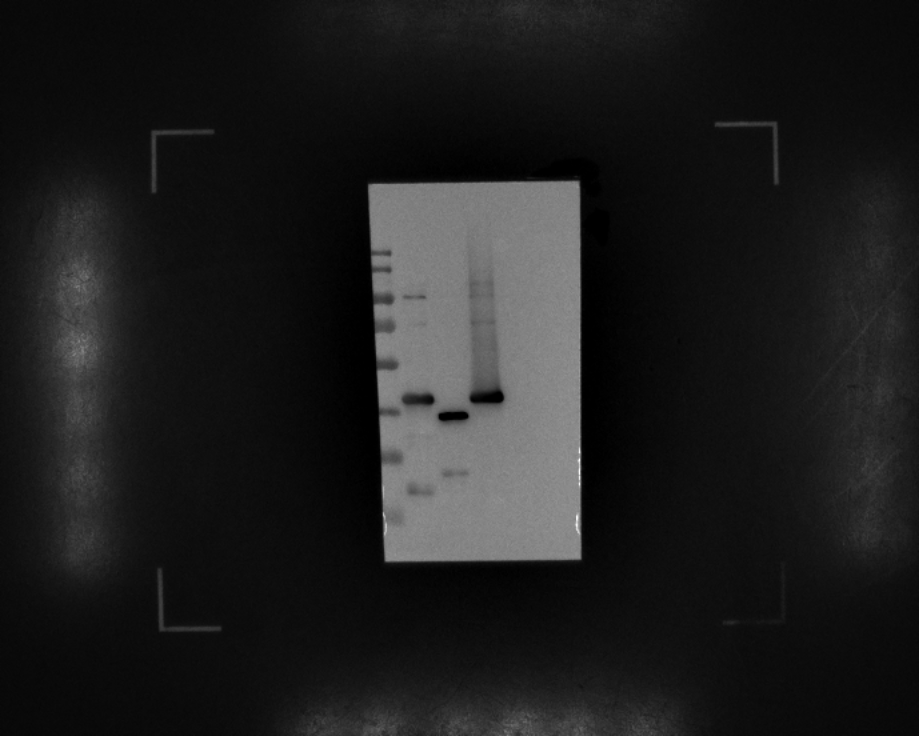

Supplement: Supplementary file 8 — Source data Fig. 6 [file 44321_2024_167_MOESM8_ESM.zip › EMM-2024-20280_Source data for Figure 6/6D/FALG.tif]

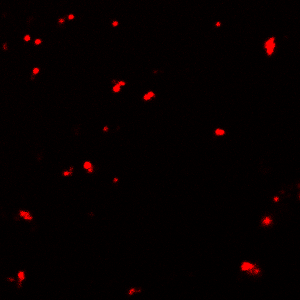

Supplement: Supplementary file 8 — Source data Fig. 6 [file 44321_2024_167_MOESM8_ESM.zip › EMM-2024-20280_Source data for Figure 6/6E/Cherry (Cherry-CREB del bZIP).tif]

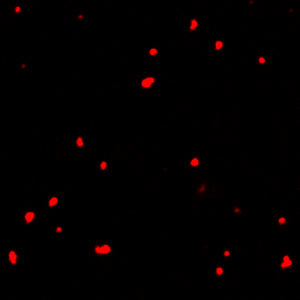

Supplement: Supplementary file 8 — Source data Fig. 6 [file 44321_2024_167_MOESM8_ESM.zip › EMM-2024-20280_Source data for Figure 6/6E/Cherry (Cherry-CREB FL+FITC-MMPP).tif]

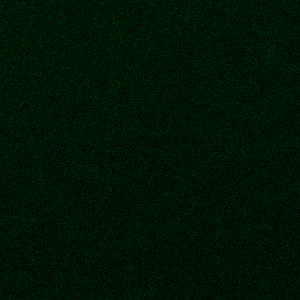

Supplement: Supplementary file 8 — Source data Fig. 6 [file 44321_2024_167_MOESM8_ESM.zip › EMM-2024-20280_Source data for Figure 6/6E/FITC (Cherry-CREB del bZIP).tif]

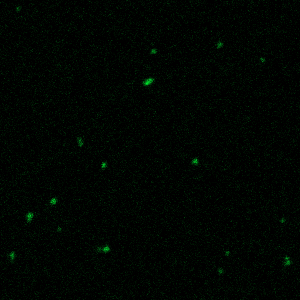

Supplement: Supplementary file 8 — Source data Fig. 6 [file 44321_2024_167_MOESM8_ESM.zip › EMM-2024-20280_Source data for Figure 6/6E/FITC (Cherry-CREB FL+FITC-MMPP).tif]

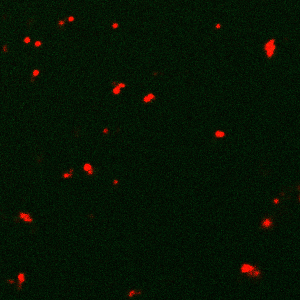

Supplement: Supplementary file 8 — Source data Fig. 6 [file 44321_2024_167_MOESM8_ESM.zip › EMM-2024-20280_Source data for Figure 6/6E/Merge (Cherry-CREB del bZIP).tif]

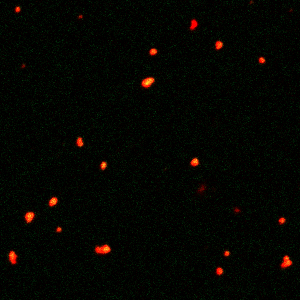

Supplement: Supplementary file 8 — Source data Fig. 6 [file 44321_2024_167_MOESM8_ESM.zip › EMM-2024-20280_Source data for Figure 6/6E/Merge (Cherry-CREB FL+FITC-MMPP).tif]

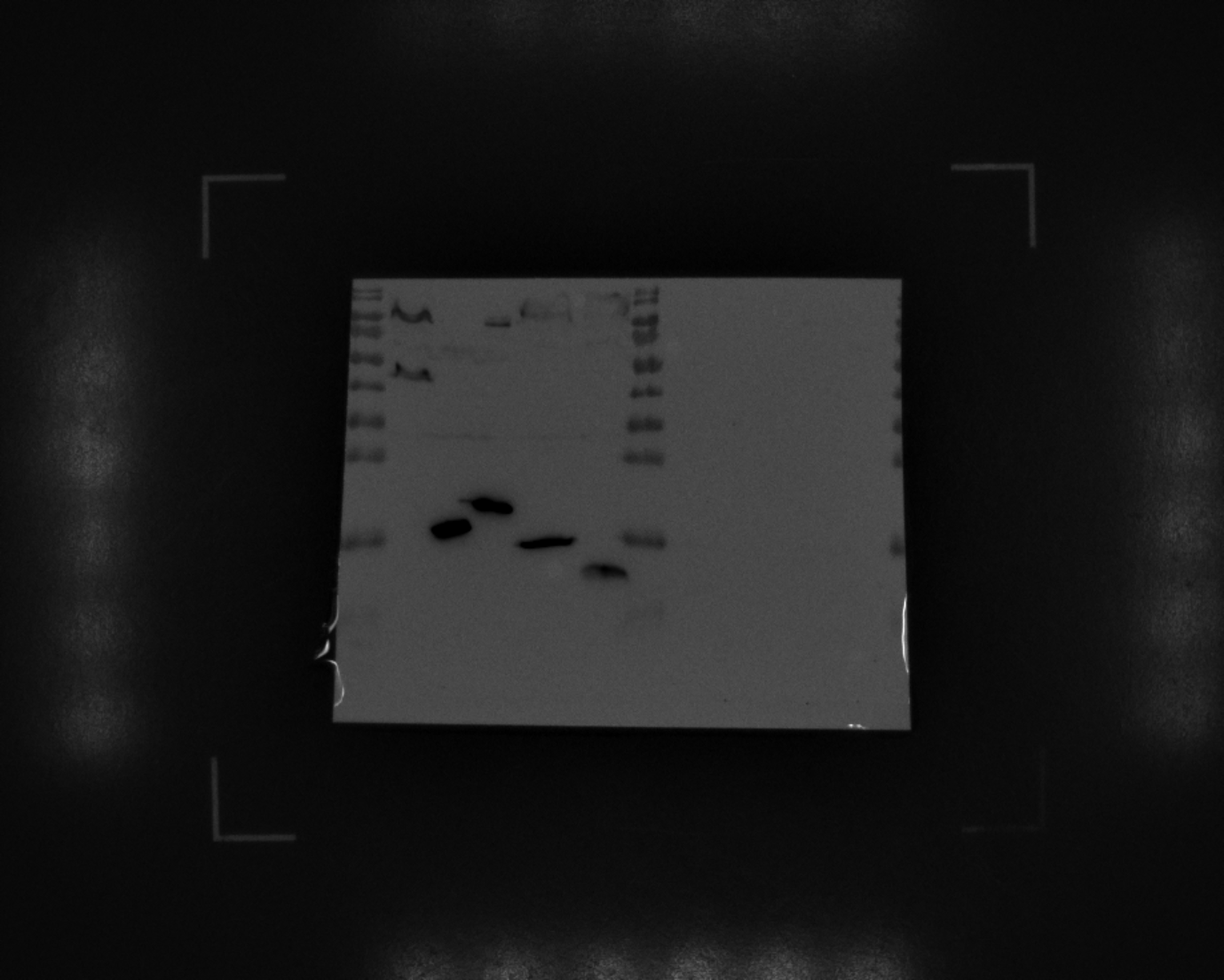

Supplement: Supplementary file 8 — Source data Fig. 6 [file 44321_2024_167_MOESM8_ESM.zip › EMM-2024-20280_Source data for Figure 6/6F/FLAG Input.tif]

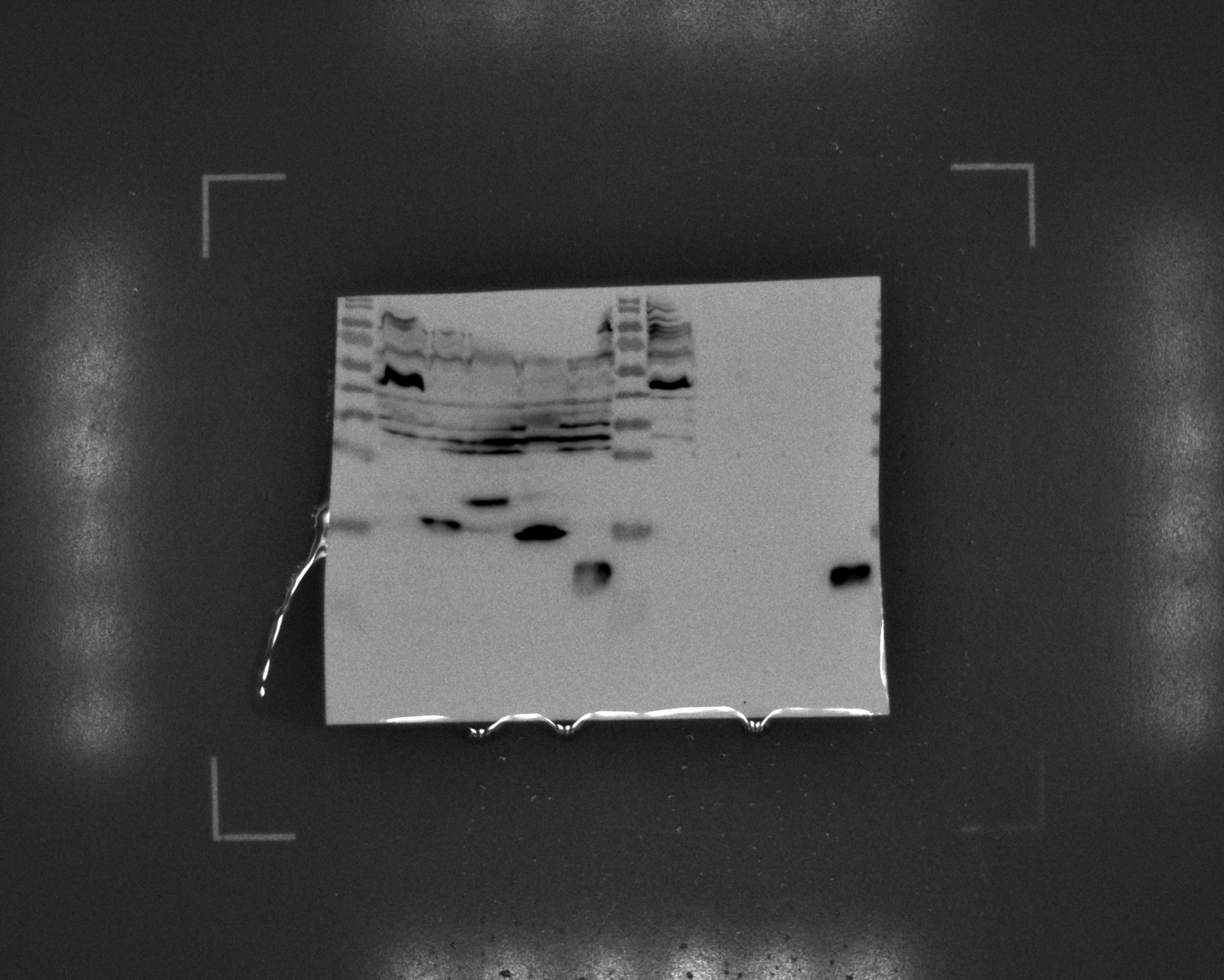

Supplement: Supplementary file 8 — Source data Fig. 6 [file 44321_2024_167_MOESM8_ESM.zip › EMM-2024-20280_Source data for Figure 6/6F/FLAG pull down.tif]

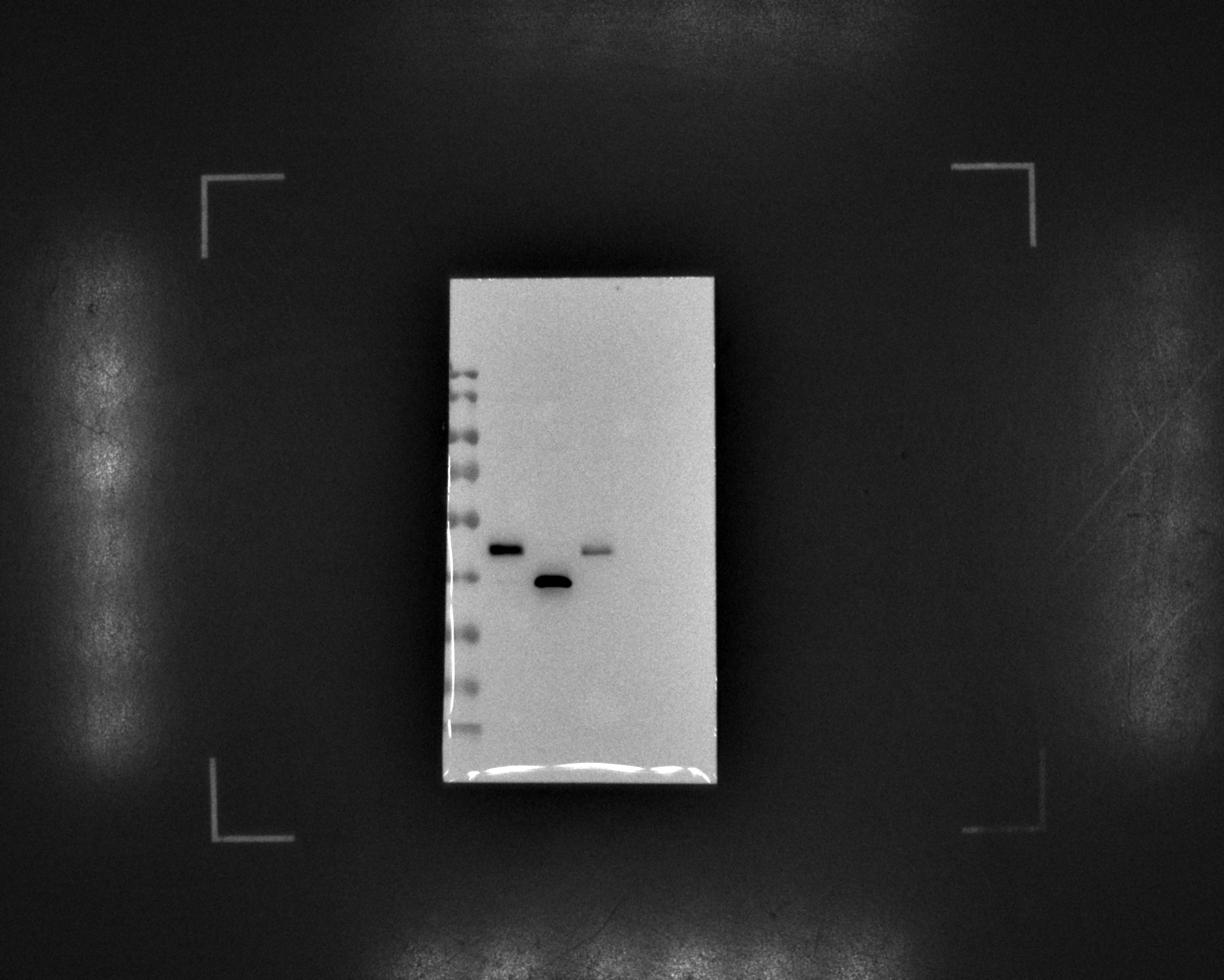

Supplement: Supplementary file 8 — Source data Fig. 6 [file 44321_2024_167_MOESM8_ESM.zip › EMM-2024-20280_Source data for Figure 6/6G/FLAG.tif]

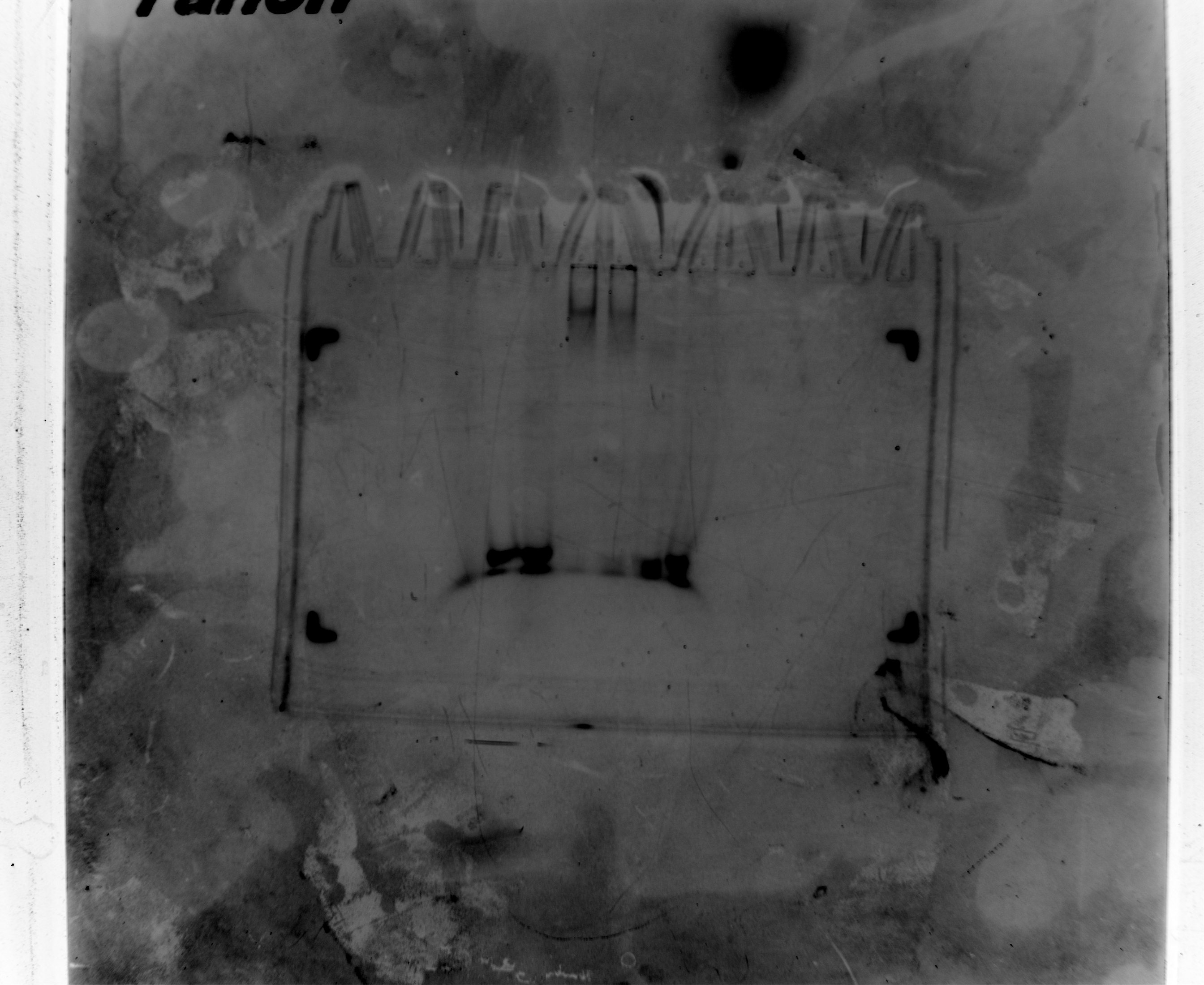

Supplement: Supplementary file 8 — Source data Fig. 6 [file 44321_2024_167_MOESM8_ESM.zip › EMM-2024-20280_Source data for Figure 6/6H/EMSA.BMP]

Figure 6C

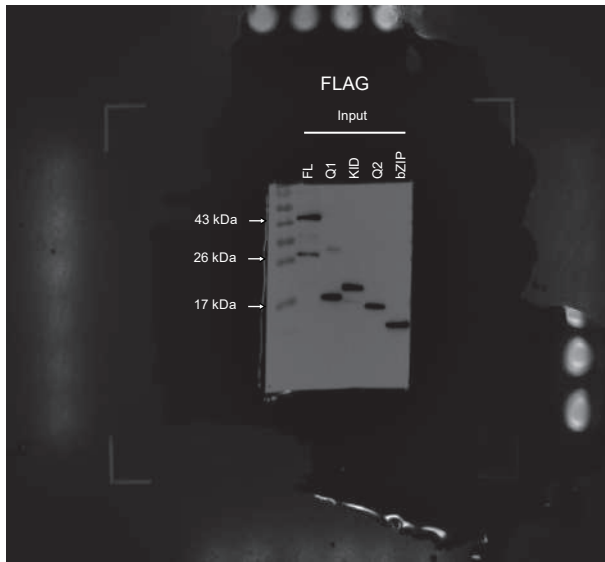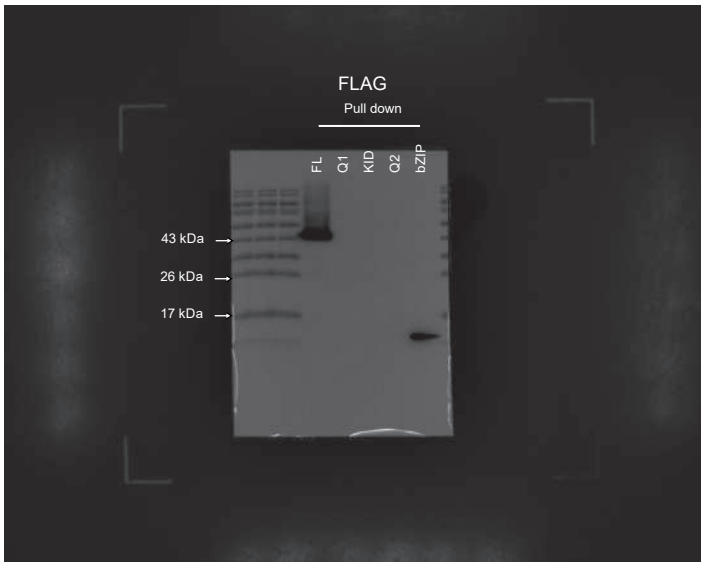

Figure 6D

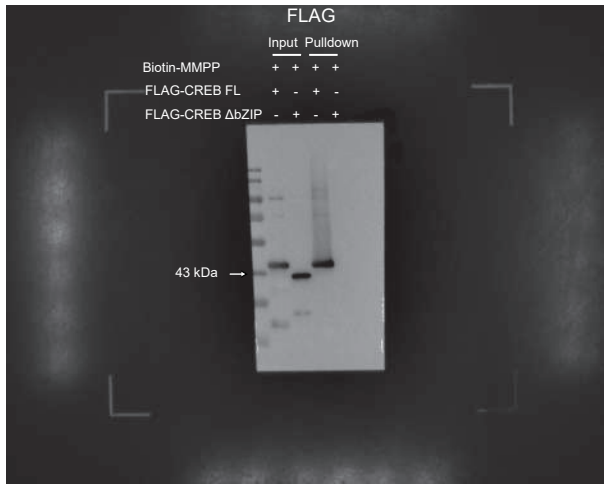

Figure 6F

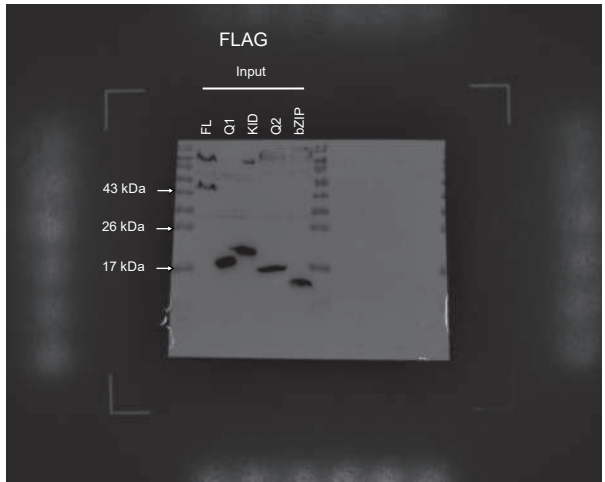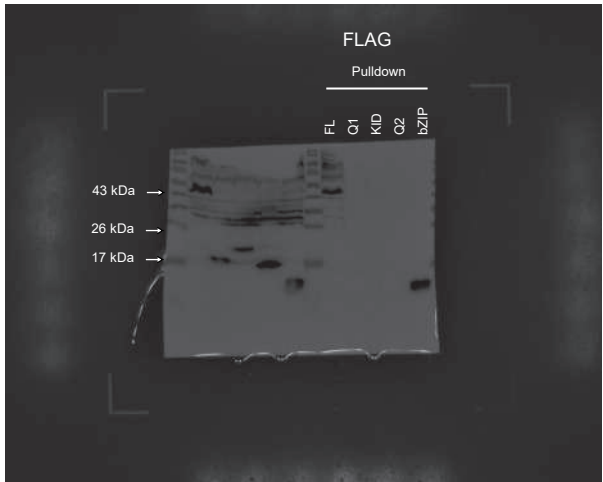

Figure 6G

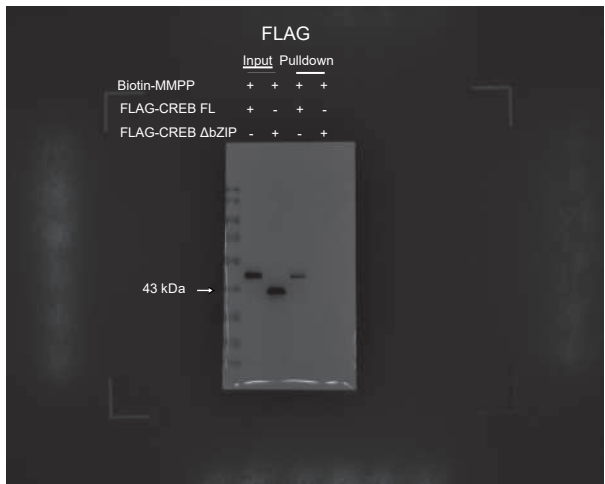

Figure 6H

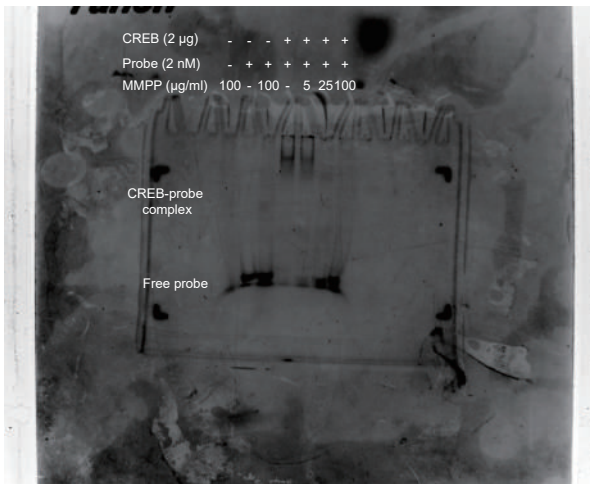

Supplement: Supplementary file 8 — Source data Fig. 6 [file 44321_2024_167_MOESM8_ESM.zip › EMM-2024-20280_Source data for Figure 6/README for gels.pdf]
