# Supplementary material for: Melanin-like nanoparticles slow cyst growth in ADPKD by dual inhibition of oxidative stress and CREB
Source: EMBO Mol Med. 2024 Nov 20;17(1):169–92. doi: 10.1038/s44321-024-00167-2 (PMC11730739; doi:10.1038/s44321-024-00167-2)
Supplement: Supplementary file 9 — Expanded View Figures [file 44321_2024_167_MOESM9_ESM.pdf]

Expanded View Figures

Figure EV1. MMPP inhibits cyst growth and scavenges ROS in in vitro and ex vivo ADPKD models.

(**A**) Representative images (left) and cyst diameter (right) of MDCK cysts treated with the indicated doses of MMPP (representative of eight independent experiments; $n = 8$ biological replicates). (**B**) DHE staining (left) and quantification (right) of cysts in each of the indicated groups (representative of five independent experiments; $n = 5$ biological replicates). (**C**) Representative images (left) of mouse embryonic kidneys treated with the indicated doses of MMPP and quantification (right) of the percentage of cyst area relative to total kidney area (representative of three independent experiments; $n = 3$ biological replicates). (**D**) DHE staining (left) and quantification (right) of embryonic kidneys on day 6 of MMPP treatment (representative of three independent experiments; $n = 3$ biological replicates). Scale bars, 100 µm (**A** and **B**) and 1 mm (**C** and **D**). Data presented as means ± SD. One-way ANOVA with LDS test or Dunnett's T3 test was used for statistical analysis in (**A**), (**B**) and (**D**), repeated measures ANOVA with LSD test was used for statistical analysis in (**C**).

 © The Author(s)

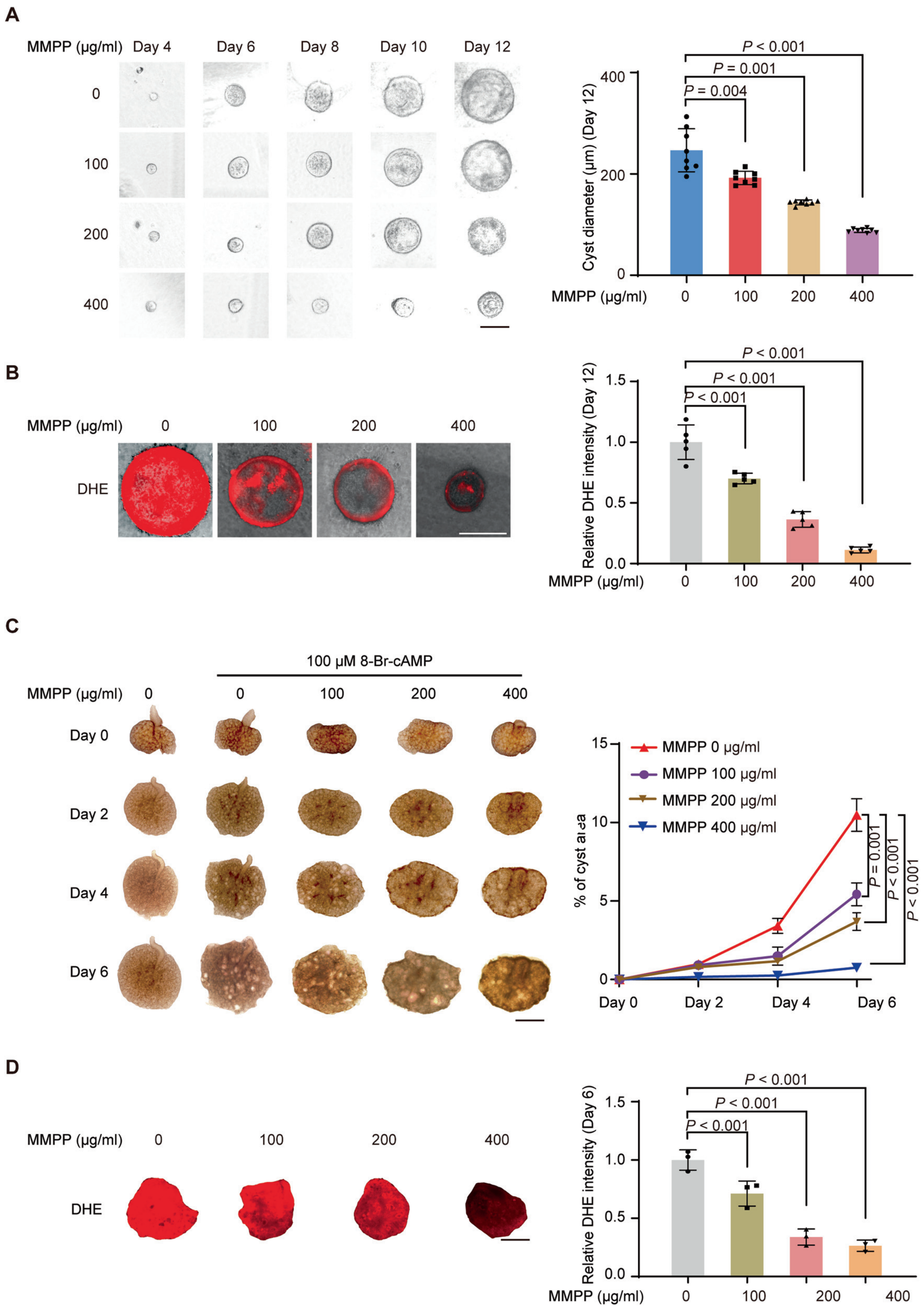

A
MMPP (μg/ml)
Day 4
Day 6
Day 8
Day 10
Day 12
0
100
200
400
Cyst diameter (μm) (Day 12)
P < 0.001
P = 0.001
P = 0.004
B
MMPP (μg/ml)
DHE
Relative DHE intensity (Day 12)
P < 0.001
P < 0.001
P < 0.001
C
100 μM 8-Br-cAMP
MMPP (μg/ml)
Day 0
Day 2
Day 4
Day 6
MMPP 0 μg/ml
MMPP 100 μg/ml
MMPP 200 μg/ml
MMPP 400 μg/ml
% of cyst area
P = 0.001
P < 0.001
P < 0.001
D
MMPP (μg/ml)
DHE
Relative DHE intensity (Day 6)
P < 0.001
P < 0.001
P < 0.001

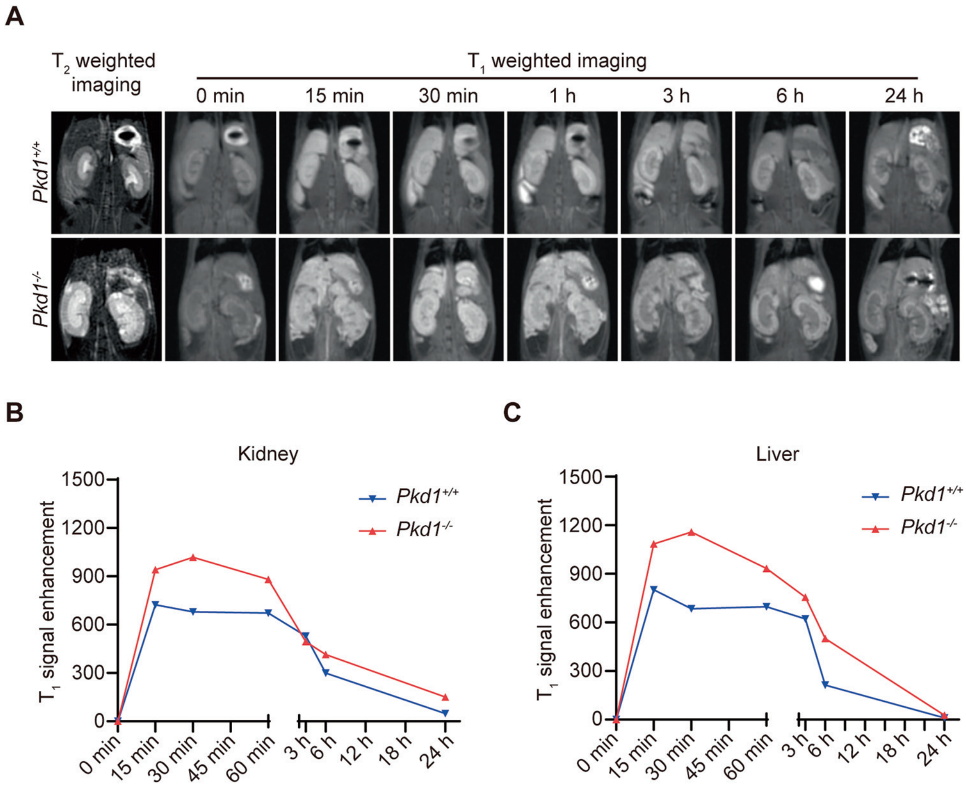

Figure EV2. MR imaging of MMPP distribution in kidneys and liver.

(**A**) MR images of MMPP in *Pkd1*$^{+/+}$ and *Pkd1*$^{-/-}$ mice (Dosage: 100 mg/kg). (**B**) Quantification of MMPP T_1 signal in kidneys. (**C**) Quantification of MMPP T_1 signal in livers.

 © The Author(s)

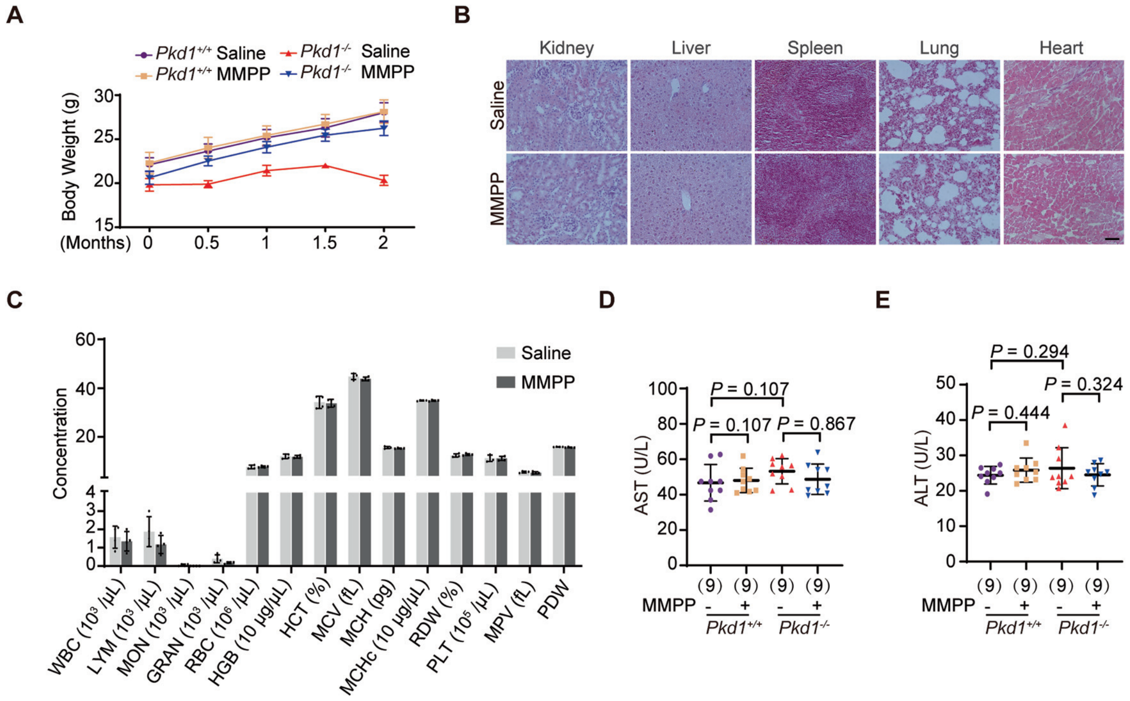

Figure EV3. Safety assessment of MMPP in vivo.

(**A**) Growth curve of mouse body weight of *Pkd1*$^{+/+}$ and *Pkd1*$^{-/-}$ mice treated with saline or MMPP ($n \geq 3$) (**B**) H&E staining of organs from mice treated with Saline or MMPP. (**C**) Hematology analysis of whole blood in MMPP-treated mice ($n = 4$). (**D**, **E**) AST (**D**) and ALT (**E**) levels in MMPP-treated mice from the indicated groups ($n = 9$). Scale bar, 50 μm. Data presented as means ± SD. Two-way ANOVA with LSD test was used for statistical analysis.

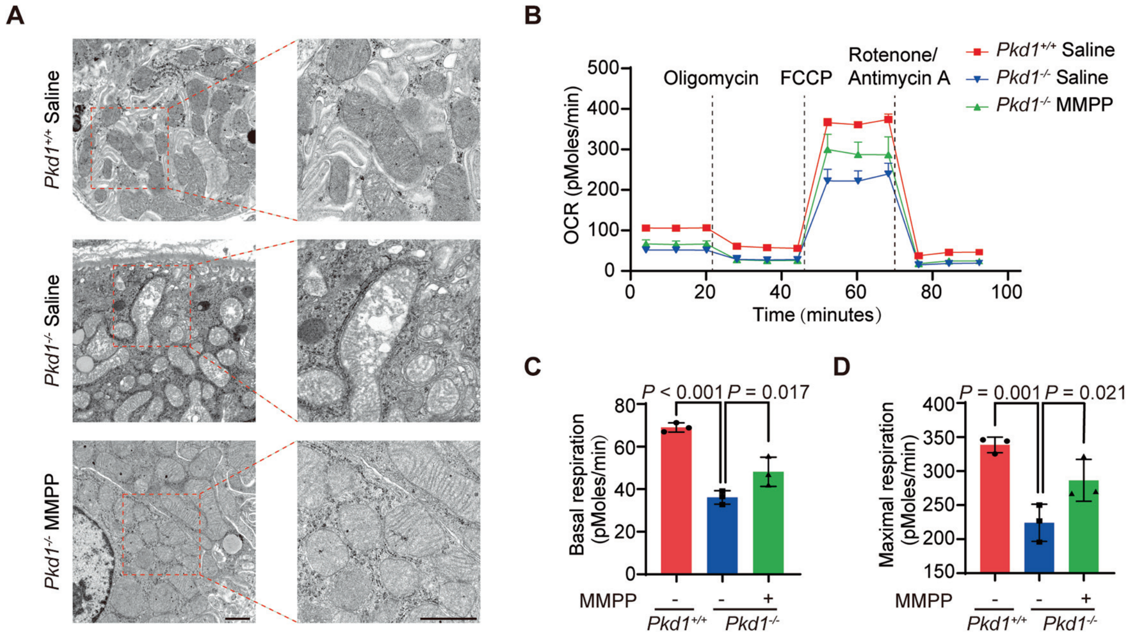

Figure EV4. MMPP treatment improves mitochondria morphology and metabolic function in ADPKD kidneys.

(**A**) TEM images of mitochondria from kidney tissues of the indicated groups. (**B**) Measurement of the mitochondrial OCR of renal primary tubule cells isolated from the indicated groups (representative of three independent experiments; $n = 3$ biological replicates). (**C**) Basal respiration of mitochondria from the indicated groups (representative of three independent experiments; $n = 3$ biological replicates). (**D**) Maximal respiration of mitochondria from the indicated groups (representative of three independent experiments; $n = 3$ biological replicates). Scale bars, 1 µm. Data presented as means ± SD. One-way ANOVA with LSD test was used for statistical analysis in (**C**) and (**D**).

 © The Author(s)
